# Integration of Maps Enables a Cytogenomics Analysis of the Complete Karyotype in *Solea senegalensis*

**DOI:** 10.3390/ijms23105353

**Published:** 2022-05-11

**Authors:** Daniel Ramírez, María Esther Rodríguez, Ismael Cross, Alberto Arias-Pérez, Manuel Alejandro Merlo, Marco Anaya, Silvia Portela-Bens, Paulino Martínez, Francisca Robles, Carmelo Ruiz-Rejón, Laureana Rebordinos

**Affiliations:** 1 Área de Genética, Facultad de Ciencias del Mar y Ambientales, INMAR, Universidad de Cádiz, 11510 Cádiz, Spain; daniel.ramirez@uca.es (D.R.); mariaesther.rodriguez@uca.es (M.E.R.); ismael.cross@uca.es (I.C.); alberto.arias@uca.es (A.A.-P.); alejandro.merlo@uca.es (M.A.M.); marco.anaya@alum.uca.es (M.A.); silvia.portela@uca.es (S.P.-B.); 2 Departamento de Zoología, Genética y Antropología Física, Universidad de Santiago de Compostela, 27002 Lugo, Spain; paulino.martinez@usc.es; 3 Departamento de Genética, Universidad de Granada, 18071 Granada, Spain; frobles@ugr.es (F.R.); carmelo@ugr.es (C.R.-R.)

**Keywords:** *Solea senegalensis*, pleuronectiformes, genetic maps, cytogenomics, chromosome evolution, karyotype, repetitive sequences, comparative genomics

## Abstract

The Pleuronectiformes order, which includes several commercially-important species, has undergone extensive chromosome evolution. One of these species is *Solea senegalensis*, a flatfish with 2*n* = 42 chromosomes. In this study, a cytogenomics approach and integration with previous maps was applied to characterize the karyotype of the species. Synteny analysis of *S. senegalensis* was carried out using two flatfish as a reference: *Cynoglossus semilaevis* and *Scophthalmus maximus*. Most *S. senegalensis* chromosomes (or chromosome arms for metacentrics and submetacentrics) showed a one-to-one macrosyntenic pattern with the other two species. In addition, we studied how repetitive sequences could have played a role in the evolution of *S. senegalensis* bi-armed (3, and 5–9) and acrocentric (11, 12 and 16) chromosomes, which showed the highest rearrangements compared with the reference species. A higher abundance of TEs (Transposable Elements) and other repeated elements was observed adjacent to telomeric regions on chromosomes 3, 7, 9 and 16. However, on chromosome 11, a greater abundance of DNA transposons was detected in interstitial BACs. This chromosome is syntenic with several chromosomes of the other two flatfish species, suggesting rearrangements during its evolution. A similar situation was also found on chromosome 16 (for microsatellites and low complexity sequences), but not for TEs (retroelements and DNA transposons). These differences in the distribution and abundance of repetitive elements in chromosomes that have undergone remodeling processes during the course of evolution also suggest a possible role for simple repeat sequences in rearranged regions.

## 1. Introduction

The study of vertebrate karyotypes has presented researchers with diverse challenges in systematics and evolution. In fish, cytogenetics has played a less significant role, partly because of the difficulty of obtaining adequate live samples (individuals, tissues or cells) of, for example, deep-sea fish to obtain their karyotypes. Even when suitable samples are available, the application of cytogenetic techniques is complex and laborious, and there is no guarantee of obtaining good chromosomal observations. However, the application of karyotype data together with morphology, genome size and sequence data can produce more robust results in the evolution and classification of organisms. In particular, the application of fluorescence in situ hybridization (FISH) and derived techniques has been very useful, for example, to study the origin and evolution of sex chromosomes in teleost species [1,2,3].

Teleosts are a group of fishes extremely diverse in their morphology, behaviour and genetics. This diversity could be related to a whole-genome duplication that took place in this group before its diversification, in addition to the two duplications that occurred during the origin of vertebrates. Different lines of evidence, such as the number of chromosomal rearrangements, the functionalisation of duplicated genes, the rate of protein evolution, and conservation of non-coding elements, show a higher rate of evolution in teleosts than in other vertebrates [4]. This group has small chromosomes and an ancestral karyotype of 48 acrocentric chromosomes [5,6].

Within the teleosts, the genome size of the order Pleuronectiformes is among the smallest of all fishes, ranging in size from 400 to 650 Mb [7,8,9]. Their chromosomes are very small [10], with diploid chromosome numbers ranging from 2*n* = 28 to 2*n* = 48 [11]; and they have undergone extensive chromosome evolution, as it is shown by the karyotype formula of the three species compared in this study: *S. senegalensis* has *n* = 21 chromosomes composed of 3 MT + 2 SMT + 4 STL + 12 TL [12]; *C. semilaevis* has *n* = 21 TL chromosomes [13]; and *S. maximus* has *n* = 22 chromosomes composed of 2 MT + 1 SMT/STL + 5 STL + 14 TL [14]. 

The phylogeny of the order Pleuronectiformes has been disputed, with some works supporting a monophyletic origin [15,16] and others a poly/paraphyletic one [17,18,19]. However, genome analysis of 11 flatfish species suggests that the suborders Pleuronectoidei and Psettodoidei originated from distinct Percoid ancestors, making the Pleuronectiformes a polyphyletic group [20].

Cytogenomics appears to be particularly useful in the study of chromosome organisation and evolution in non-model organisms [1,2,21]. Furthermore, in fish, physical mapping of genes is especially important since the size of chromosomes makes their individual identification difficult and causes a high degree of genetic interference [22].

The combination of information from different types of maps, such as linkage and physical maps, allows the creation of so-called “integrated” maps. These maps facilitate fine mapping of quantitative trait loci (QTL), positional cloning, genome sequencing and assembly, and make genome-wide comparative studies possible [23,24,25]. Among the different methodologies that enable the creation of integrated maps is the combination of FISH and bacterial artificial chromosome (BAC) genomic libraries. BAC clones carry long DNA fragments that allow the development of multiple probes per chromosome. This, together with their sequencing by Next Generation Sequencing (NGS), facilitates the anchoring of linkage and genomic data to chromosomes [22]. BAC libraries have proven to be essential for the identification of complete genomic sequences for the integration of genetic and physical maps and comparative genomic studies [24]. 

With technological advances, genome sequencing has become almost routine, even for non-model organisms. Within the Pleuronectiformes, the number of genomes available for study has increased considerably in recent years [20], including species of commercial interest such as turbot [26], Japanese flounder [27] and sole [7]. Only for a few species has a level of chromosome assembly been reached. Although the information provided by these genomes is relevant at both basic (e.g., chromosomal evolution and metamorphosis mechanisms) and applied levels (e.g., growth performance and disease resistance), further refinement is needed to make them useful for association studies, gene mapping and comparative genomics.

The Senegalese sole (*Solea senegalensis* (Kaup, 1858)) is a flatfish belonging to the order Pleuronectiformes. The species is widely distributed in the waters of the Atlantic Ocean, from the Gulf of Biscay to the northwest coast of Africa, and in the Mediterranean Sea, from the Strait of Gibraltar to Tunisia. It is a sole of great commercial value based on high demand and a reasonable price [28]. However, some problems hinder its production [28]: (1) high larval mortality, (2) sub-optimal larval weaning strategies, and (3) inadequate disease control. Therefore, in order to ensure sustainability and improved production, it is necessary to study the influence of genetics on the productive traits, physiology and immunology of the species. 

Considerable effort has been made in recent years to understand the cytogenetic and genomic aspects of this species. Molina-Luzón et al. [29] developed the first haploid genetic map of Senegalese sole using gynogenetic families and 129 microsatellites, with the consensus map consisting of 27 linkage groups (LGs). Several studies have used a BAC-FISH approach to locate and map genes on the chromosomes, as well as to study aspects such as histone evolution [30], sex determination and differentiation [31,32,33], repetitive DNA families [34], and chromosomal evolution [35]. This has made it possible to improve the integrated genetic map [36] and to propose that the evolution of several Senegalese sole chromosomes (e.g., the largest metacentric chromosome, i.e., chromosome 1, and chromosomes 2 and 4) is due to Robertsonian fusions, pericentric inversions, and other chromosomal rearrangements by transposable elements. Guerrero-Cózar et al. [37] analysed a draft genome to identify microsatellites. By combining the microsatellites identified in that work with microsatellites from Molina-Luzón et al. [29] it was possible to construct an integrated map containing 21 linkage groups, which matches the chromosomal number of the species. Subsequently, a chromosome-level assembly was presented [7] for the genome of a male *S. senegalensis*, obtained by de novo assembly and construction of a linkage map with ddRAD markers. However, the linkage groups identified in these latter two works have not been related to chromosomes.

Hence, the purpose of this work is to obtain new information, integrate it with previous maps and the genome sequence of *S. senegalensis*, and relate these results to the knowledge at the chromosomal level, thus advancing the study of the complete karyotype of the species and its evolution. 

## 2. Results

### 2.1. Cytogenetic Map

**Figure 1:**
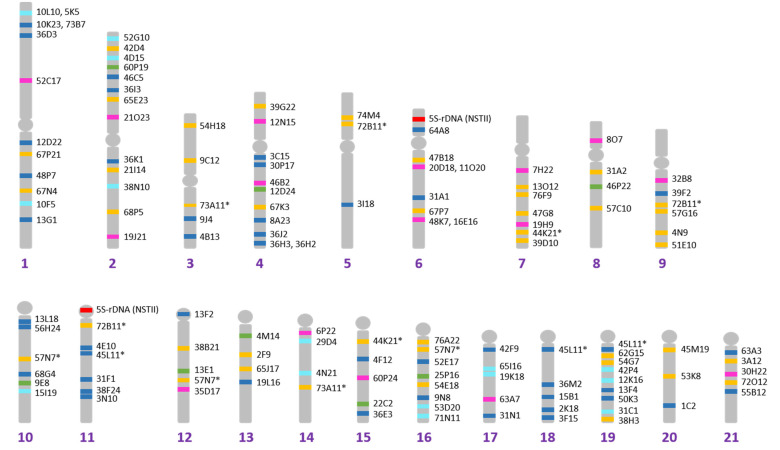
Cytogenetic map of *Solea senegalensis* obtained with BAC probes: Yellow color indicates BACs containing microsatellites; pink color indicates BACs with genes related to sexual reproduction/differentiation; green color indicates BACs with genes related to metamorphosis; clear blue color indicates BACs containing genes related to the immune system; and dark blue color indicates BACs with genes belonging to other categories. Short arms are *p* and long are *q* (upper is *p* and lower is *q* in metacentrics). * BACs found on more than one chromosome.

A total of 126 BAC clones were mapped to the Senegalese sole chromosome complement (Table S1 [38,39,40,41], Figure 1 and Figure S1). Sixteen of these clones contain candidate genes related to the immune system, eighteen related to sex determination or reproduction, eight related to metamorphosis, forty-six were randomly taken from the BAC library (anonymous), and thirty-eight contain microsatellite markers used by Molina-Luzón et al. [29] to generate the genetic map (Table S2). Five BAC clones were located on two or three chromosomes, and discarding these, an average of 4.7 BAC clones were positioned per chromosome arm (considering metacentric and submetacentric ones as bi-armed). However, some arms presented a higher number of BAC clones, such as the p arm of chromosome 2, the q arm of chromosome 4, and chromosomes 6, 16 and 19. However, for one of the largest chromosomes in the *S. senegalensis* karyotype, chromosome 5, only one BAC clone was found in each arm. A Megablast search in the chromosome level-scaffolding of *S. senegalensis* male [7] allowed a precise mapping of the BACs and the correction of previous locations of some BACs (Table S3).

### 2.2. Sequencing and Gene Annotation

All BAC clones had been sequenced in previous studies, except those containing microsatellite markers, which were sequenced for the present study. To summarise, a total of 6,726,594 bp have been sequenced; the N50 value ranged from 327 to 124,351 bp, and the L50 ranged from 1 to 31 (Table S4). Up to 279 genes were annotated from the thirty-eight new BAC clones (Table S5).

### 2.3. Integration of Maps

The genetic and physical maps obtained by Molina-Luzón et al. [29] and Guerrero-Cózar et al. [7], respectively, were integrated with the cytogenetic map (Table 1). The 21 scaffolds obtained by Guerrero-Cózar et al. [7] were correlated with the 21 chromosomes of the cytogenetic map.

FISH analysis of BAC clones allowed correlation of 23 out of the 27 Linkage Groups (LGs) obtained by Molina-Luzón et al. [29] with the exception of LG5, LG17, LG23 and LG26. These LGs were located on 19 chromosomes as no BAC clones containing microsatellite markers hybridized on chromosomes 17 and 18. Four chromosomes correlated one-to-one with four LGs (chromosomes 8, 13, 19 and 21); the remaining fifteen chromosomes had one, two or three LGs that are repeated on one or more additional chromosomes.

### 2.4. Synteny Analysis

The analysis was performed for each chromosome of *S. senegalensis* in comparison with the orthologous regions of *C. semilaevis* and *S. maximus* (Figures S2–S37). This allowed us to produce a comparative map among the three species (Figure 2 and Tables S6 and S7). In this map, the BAC clones of each chromosome and each chromosome arm (for metacentrics and submetacentrics) of *S. senegalensis* have their own colour to more easily distinguish between them and to trace more easily the syntenic positions in the other species. One of the main results is that, for most of the *S. senegalensis* chromosomes (or chromosome arms, for metacentrics and submetacentrics), there is a corresponding orthologous chromosome in the other two species.

**Figure 2:**
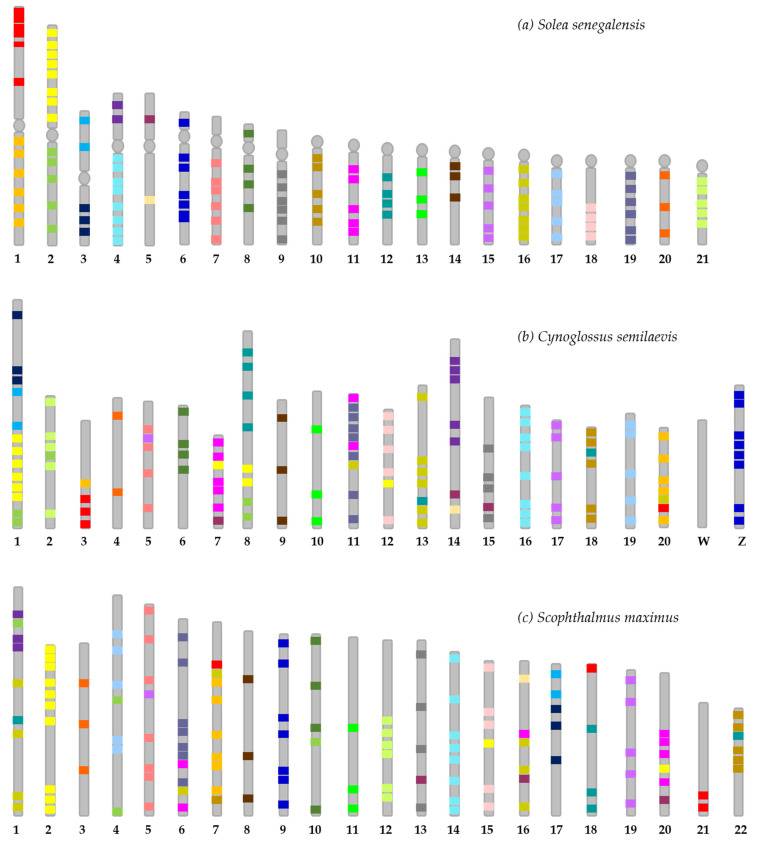
Comparative mapping of BAC clones localized in (**a**) *Solea senegalensis* with (**b**) *Cynoglossus semilaevis*, and (**c**) *Scophthalmus maximus*. BAC clones located in the same chromosome arm (considering metacentric and submetacentric chromosomes as bi-armed) of *S. senegalensis* are represented with the same color.

The first metacentric pair of *S. senegalensis* has two orthologue chromosomes in *C. semilaevis* (3 and 20) and three in *S. maximus* (7, 18 and 21), and most of the arm-specific BAC clones are on one chromosome of the other two species, with the exception of only two BAC clones. In the second metacentric pair, its BAC clones are more dispersed throughout the chromosome complement of both *C. semilaevis* and *S. maximus*. However, those of the p arm (yellow blocks in Figure 2) appear to be more conserved at a major orthologous locus (chromosome 1 in *C. semilaevis* and chromosome 2 in *S. maximus*), although some BACs are partially located on other chromosomes. The BAC clones of the q arm (green blocks in Figure 2) do not have a major orthologous locus; instead, BAC clones are more widely distributed. The BAC clones of the third metacentric pair are on a single orthologous chromosome in both *C. semilaevis* (chromosome 1) and *S. maximus* (chromosome 17).

The BAC clones found on each arm of submetacentric pair 4 are present on two different chromosomes in the other species. Specifically, the BAC clones of the p arm are found on chromosome 14 of *C. semilaevis* and on chromosome 1 of *S. maximus*, whereas those on the q arm are found on chromosomes 16 and 14 of *C. semilaevis* and *S. maximus*, respectively. The situation is different in the case of submetacentric pair 5 of *S. senegalensis*. First, this is the chromosome with the lowest number of BAC clones found (only two, one on each arm), and second, they are located on only one chromosome in the other species, i.e., chromosome 14 in *C. semilaevis* and chromosome 16 in *S. maximus*. However, the relative position of these two BAC clones differs among the three species; in *S. senegalensis* and *S. maximus* the two BAC clones far apart, but in *C. semilaevis* they are closer.

As for the four subtelocentric pairs (from pair 6 to 9), all of them show that the BAC clones of each chromosome are located on an orthologous chromosome in each of the other two species. Specifically, subtelocentric pair 6 corresponds to chromosomes Z and 9 of *C. semilaevis* and *S. maximus*, respectively; subtelocentric pair 7 to chromosomes 5 and 5; subtelocentric pair 8 to 6 and 10; and subtelocentric pair 9 to 15 and 13. Strikingly, the BAC clones of subtelocentric chromosome 6 localize to the Z chromosome of *C. semilaevis*, but not to the W chromosome, apart from a few genes.

The telocentric chromosomes of *S. senegalensis* can be divided between those in which its BAC clones are scattered on different chromosomes of both *C. semilaevis* and *S. maximus*, and those in which the BAC clones are all together on one orthologous chromosome. Figures S38 and S39 of the supplementary material show the distribution of BAC clones on each telocentric chromosome among the chromosome complement of the other two species.

### 2.5. Study of Repeated Sequence

To gain further insights into how repetitive sequences might have played a role in the evolution of bi-armed chromosomes (chromosomes 1–9) in *S. senegalensis*, the abundance and coverage of transposons, satellites, simple repeats, small RNA and low complexity sequences on chromosomes were analysed (Figure S40.1–13; Tables S8 and S9). The repetitive elements on chromosomes 1, 2 and 4 were analysed previously [33,34,35]; therefore, the remaining bi-armed chromosomes (3, 5–9) are analysed in the present work. In addition, chromosomes 11 and 16 were also analysed to determine the repetitive elements involved in chromosome translocations and rearrangements and their evolution. Finally, *S. senegalensis* presents an XX/XY determination system but lacks heteromorphic sex chromosomes. The presence of a recent putative sex-determining gene described on chromosome 12 [7] makes this chromosome of interest to study the possible abundance of repetitive elements, as is observed in sex chromosomes [42,43].

**Figure 3:**
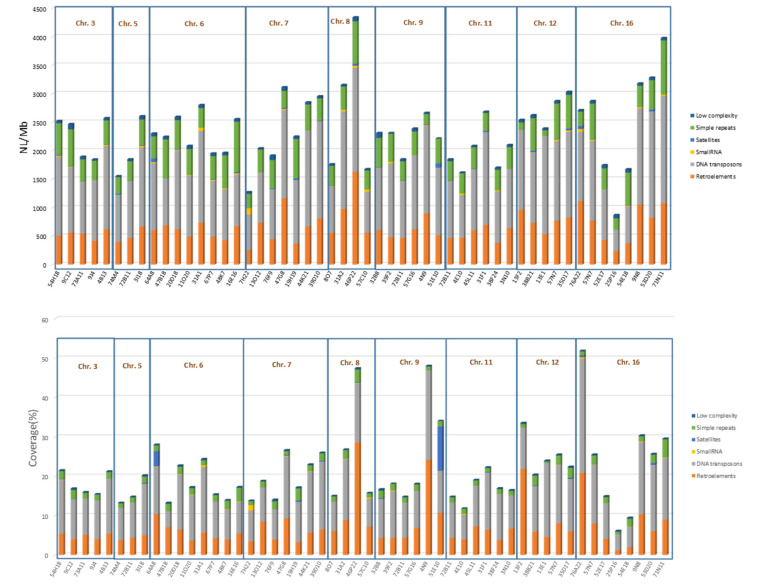
Number of loci per Mb (NL/Mb) and coverage (%) of repeat elements in BACs from chromosomes 3, 5–9, 11, 12 and 16 of *Solea senegalensis*: DNA transposons, retroelements, small RNA, satellites, simple repeats, low complexity.

It can be estimated that the coverage of the six repetitive elements analysed in this study is 20% and the number of loci per Mb is 2500 in *S. senegalensis* chromosomes (Figure 3 and Figure S40; Tables S8 and S9).

A summary of the most relevant results obtained after the analysis of repetitive elements in the BACs mapped along the eight chromosomes is shown in Table S10.

Considering the BACs as genomic samples of the chromosomes, and measuring the mean values of the number of loci per chromosome, it can be seen that chromosome 12 has the highest mean number of DNA transposons of all chromosomes analysed, and also has low variance (Figure S40.12). The highest mean number of retroelements is found on chromosome 8, followed by chromosomes 12 and 16. At the family level, the highest mean numbers of hobo-Activator and Tc1-IS630-Pogo are found on chromosome 12. In relation to repetitive elements coverage, the highest mean number of DNA transposons is found on chromosomes 16 and 12. Retroelements have, on average, the highest coverage on chromosomes 8, 9 and 12. Chromosome 12 has the highest mean coverage values for hobo-Activator, L2/C1/Rex and Tc1-IS630-Pogo elements. The most abundant LINEs, on average, are on 8 and 12, and LTRs are on 8 and 9 (Figure S40.13).

## 3. Discussion

The density of the cytogenetic map of *S. senegalensis* has been increased by 33 BAC clones with respect to that obtained by Merlo et al. [36], and consequently, markers are available for all chromosomes of the complement. In addition, BLAST analysis of BACs on the chromosome-level scaffolding allowed precise mapping onto chromosomes. Finding BAC clones that hybridize on two or more chromosomes (such as BAC clones 44K21, 45L11, 57N7, 72B11 and 73A11) is indicative of the round of ancestral duplication that occurred in the teleost lineage. Due to whole genome duplication (WGD) of an organism, as could be the ancestor of teleosts, the chromosomal complement doubles (polyploidization), and rediploidization could occur when the duplicated chromosomes diverge from each other, but the rediploidized genome may leave traces of the ancestral polyploid arrangement, as evidenced in extant teleosts by the presence of many paralogs [44], making them a paleopolyploid group. The presence of many paralogues makes it more difficult to elucidate the chromosome arrangements that have taken place among the species studied. In addition, partially duplicated sequences were found within the same chromosome of the main BAC clone locus (such as BAC clones 10K23, 3I18, 64A8, 68G4, 45L11, 38B21 for chromosomes 1, 5, 6, 10, 11 and 12, respectively). Sequence duplication has been considered an important mechanism of adaptive evolution through transcriptional modulation [45].

Synteny studies showed a large orthology between the chromosomes of the three species compared, as *S. senegalensis* shares up to 15 syntenic chromosomes with *C. semilaevis* and 14 with *S. maximus*, and is in agreement with what has been previously reported in closely related species [46,47,48].

Metacentric chromosome 1 has its BAC clones distributed among chromosomes 3 and 20 of *C. semilaevis* and mainly among chromosomes 7 and 21 of *S. maximus*, which is consistent with previous studies and reinforces the view that this chromosome originates from a Robertsonian fusion event [7,30,31,32,33]. Similar origins have been postulated for chromosomes 2 (metacentric) and 4 (submetacentric) [7,35]. Our data suggest a rather dispersed distribution of BAC clones on chromosome 2, but when the analysis is performed in relation to the number of annotated genes, they are mainly found in chromosomes 1 and 8 of *C. semilaevis* and in chromosomes 2 and 4 of *S. maximus*. The *S. senegalensis* lineage arose from three Robertsonian fusions that led to chromosomes 1, 2 and 4, thus justifying the reduction from the proposed ancestral karyotype of Pleuronectiformes (*n* = 24) [11] to *n* = 21. Moreover, it has been proposed that chromosomal fusions facilitate a lineage-specific diversification, since they can generate and accumulate genetic incompatibilities [48].

The centric fusions that occurred in *S. senegalensis* account for three of the nine bi-armed chromosomes (considering subtelocentrics as bi-armed), so pericentric inversions have played a central role in the evolution of the Senegalese sole karyotype. Inversions are important rearrangements that lead to polymorphism maintenance events in populations through balancing selection and are also important in the speciation process [49,50]. In addition, there is much evidence to support the view that inversions are involved in the environmental adaptation of species and that inversion polymorphisms within a species are related geographically [51,52].

In this sense, Pleuronectiformes are fishes that have had to adapt to the heterogeneous benthic environment; therefore, each species could have adapted to local habitats through species-specific chromosome rearrangements. However, there must be a mechanism that triggers such chromosome rearrangements. Transposable Elements (TE) are considered a key mechanism for chromosomal rearrangements [21,35]. In particular, Rex retrotransposons have previously been described as a major player in the rearrangement of the largest metacentric pair of *S. senegalensis* [34]. TEs are usually suppressed by a complex epigenetic silencing pathway, but if organisms are faced with a new challenging environmental situation (such as adaptation to a new benthic lifestyle), a burst of TE reactivation would occur, leading to the generation of genomic structural variations [53], as observed in the order Pleuronectiformes.

TE activity could also explain the distribution of chromosome-specific BAC clones of specific chromosomes (or chromosome arms) of *S. senegalensis* between two or more chromosomes of the other two species. These BAC clones are those on the p arm of metacentric 1 (only with *S. maximus*), both arms of metacentric 2, and telocentric 10 (only with *S. maximus*), 11, 12 and 16. Translocations and micro-rearrangements have also been considered to be caused by TEs [35,54,55,56].

The results showed that on chromosomes 3, 7, 9 and 16, a higher abundance of TEs and other repeated elements (coverage and NL/Mb) has been observed next to the telomeric regions. This pattern has also been observed on chromosomes 1, 2 and 4 of *S. senegalensis* in a previous analysis [33,35]. However, on chromosome 11, analysis of repeated elements in mapped BACs shows a higher abundance of DNA transposons in interstitial BACs. This chromosome shows evidence of evolution from two or more chromosomes of the other two flatfish species. As described, TEs may be involved in large structural genomic variations, including translocations and others [57,58]; consequently, TEs such as DNA transposons (hobo-Activators with the highest number in the interstitial BACs) and retroelements (LINEs and L2/C1/Rex), with the highest number in this central region of the chromosome, could have caused rearrangements during the evolution of this chromosome in the flatfish group. This situation also occurs on chromosome 16 for simple repeats and low complexity sequences, but not for TEs (retroelements and DNA transposons). These differences in the distribution and abundance of repeat elements in chromosomes that have undergone remodeling processes in the course of evolution suggest a possible role of simple repeat sequences also in rearranged regions [59]. The mean number of repeat elements per chromosome shows that chromosome 12 has the highest mean number of DNA transposons.

Recently, a *S. senegalensis* linkage group (termed SseLG18) has been highlighted as a nascent sex chromosome system [7]. The follicle-stimulating hormone receptor (*fshr*), a candidate gene for sex determination, is located on SseLG18. This LG coincides with our telocentric pair 12, one of the chromosomes implicated in possible TE-mediated translocations. Transposable elements and other repetitive sequences, such as simple repeats, accumulate on the emerging sex chromosome. Suppression of recombination on sex chromosomes explains not only the expansion of repetitive DNA and the accumulation of TEs, but also the accumulation of deleterious mutations in functional genes [42]. In the BACs analysed in this work, the accumulation of repeated elements on chromosome 12 was observed, to some extent, in all elements, especially in DNA transposons, but not in large numbers. This could indicate that the process of pseudogenization and accumulation of repeated elements on this chromosome 12 represents a primitive stage of the structural changes associated with evolution as a sex chromosome.

The homologous regions of all BAC clones present on chromosome 6 are located on the Z chromosome of *C. semilaevis*, but not on the W chromosome, with the exception of very few genes. This finding reveals that a large pseudogenization process has taken place on the W chromosome of *C. semilaevis.*

## 4. Materials and Methods

### 4.1. PCR Screening, Isolation of Bac Clones and Map Integration

A total of 126 clones BACs were used in this study; they are shown in Table S1. Out of 126, 38 were BACs containing microsatellite markers (Simple Sequence Repeats, SSRs). To select these microsatellites, we initially selected 74 out of the 129 microsatellites belonging to 27 linkage groups (LGs) described by Molina-Luzón et al. [29]. The 74 microsatellites were used to screen a *S. senegalensis* library (made form larvae), which consisted of 76 plates with 29,184 clones distributed in 384-well plates. After the screening, using the 4D PCR method as described by García-Cegarra et al. [22], 38 BACs were located in the library.

The specific primers used for the screening are described in Table S2. These microsatellites were associated with the 27 LGs, except for LG5, LG17, LG23 and LG26. Out of the 74 microsatellites, 36 could not be located in the library (data not shown). Next, the positive BAC clones were isolated using the Qiagen Large-Construct Kit (Quiagen, Hilden, Germany), following the manufacturer’s recommendations. Each BAC clone was named using the BAC library coordinates according to the plate (1–76), row (A-P) and column (1–24).

BAC sequences were mapped in the chromosome-level scaffolding of *S. senegalensis* [7] by a Megablast search tool from blast algorithm [60] using the following parameters: Evalue < E−20; max_hsps = 10; sequence overlap > 5 kb. To visualise and analyse the alignments, the Integrative Genomics Viewer (IGV) program [61] and manual exploration were used.

### 4.2. BAC Sequencing and Annotation

DNA-BACs were sequenced and annotated by Lifesequencing™ (Lifesequencing; Valencia, Spain). For the sequencing, the NovaSeq6000 Illumina platform (251 cycles of paired end reads), or alternatively, the MiSeq Illumina sequencing platform (301 cycles of paired end reads), was used. The parameters related to sequencing are shown in Table S4. Reads were assembled de novo using the SPAdes Genome Assembler v3.13.0. [62]. Gene prediction and functional annotation was carried out using several software suites including Augustus v.3.3.3, Geneious 2020.1 and Blast2GO 5.2, by comparison with other fish species, such as *Danio rerio.*

### 4.3. Double and Multicolour FISH

The localization of the BACs with microsatellites in each chromosome was obtained by fluorescence in situ hybridization (FISH), in combination with BACs previously located in a cytogenetic map of *S. sengalensis* [36] used as markers. Chromosome preparations were carried out using pre-treated *S. senegalensis* larvae (from 1 to 3 days after hatching) as described in previous works [22,33]. In order to prepare the probes, DNA-BAC purification was performed using the Plasmid Midi Kit (Quiagen, Hilden, Germany). For double FISH, the DNA was labelled using Roche^®^ Biotin and Digoxigenin Nick Translation Mix, following the manufacturer’s instructions. Hybridization was carried out at 37 °C in a solution containing 10% dextran sulfate, 50% formamide, 2 × SSC, 15% SDS, and 100 ng of the labeled probe. After incubation at 37 °C, slides were washed at 43 °C with 50% formamide and 0.1 × SSC, followed by two washes in 2 × SSC. The immuno-cyto-chemistry detection was then performed using antibodies as described by Rodriguez et al. [33]. For multicolour FISH, the DNA was labelled first by DOP-PCR, and second with a conventional PCR, using different fluorochromes: Diethylaminocoumarin (DEAC) (Vysis, Downers Grove, IL, USA), Fluorescein isothiocyanate (FITC), Spectrum Orange (SO) (Abbott Molecular/ENZO, IL, USA) and Texas Red (TR) (Life Technologies, Carlsbad, California, USA) as described by Liehr et al. [63]. For chromosome staining, DAPI-Vectashield (Anti-fade Mounting Medium) was applied. FISH-BAC images were captured and examined with a Zeiss PALM MicroBeam Laser Microdissection System and a fluorescence microscope equipped with a ZEISS Axiocam 512 Microscope Camera 12 mp.

### 4.4. Comparative Genomics

For synteny analyses, two species of flatfish were used as a reference, *Cynoglossus semilaevis* (2*n* = 42) and *Scophthalmus maximus* (2*n* = 44). The BLAST tool of the Ensembl database was used for comparing the gene sequences of *S. sengalensis* with the reference species.

### 4.5. Analysis of Repeated Sequences

Following the synteny analysis performed in *S. senegalensis*, a repeat element analysis of BACs located on bi-armed chromosomes (1–9), acrocentrics 11 and 16, and chromosome 12, was carried out. Chromosomes 11 and 16 are chromosomes that have arisen during evolution by rearrangements of two or more chromosomes of other flatfish species, with a possible role of repeated sequences. Chromosome 12 is carrier of a sex-determining gene, as reported in a recently published paper [7].

The software Repeat Masker v.4.1.2. [64] run with rmblastn version 2.10.0+ and database CONS-Dfam_3.4, was used. The general repeat elements analysed were: simple repeats, satellite sequences, low complexity elements, small RNA and transposable elements (retroelements and DNA transposons). Of these transposable elements (Class I and II), subclasses, superfamilies and families were analysed. The parameters used to measure the abundance of repeated elements of each BAC were the number of loci per Mb (NL/Mb) and their coverage, measured as percentage of length occupied by repeated elements per BAC analysed (%) [34,35].

## 5. Conclusions

An extensive integrated map of *S. senegalensis* and its integration with previous maps and sequencing data at the chromosome level is presented. Syntenic studies with two flatfish species and analysis of repetitive sequences indicate differences in the abundance and distribution of repetitive elements on chromosomes that have undergone remodelling processes in the course of evolution. These results suggest a possible role of simple repetitive sequences in the rearranged regions.

## Figures and Tables

**Table 1:** Integration of the cytogenetic map of *Solea senegalensis* with published linkage map and physical map.

Chromosome of *Solea senegalensis*	Linkage Group *	Chromosome Level-Scaffolding **
1	21, 27	1
2	7, 12, 15	3
3	1, 4, 15	16
4	18, 25	2
5	9, 11	21
6	4, 27	5
7	3, 4, 14	7
8	6	10
9	1, 11, 13	12
10	19	20
11	11	17
12	19	18
13	10	11
14	1	6
15	14	19
16	16, 19, 20	9
17	-	13
18	-	15
19	2	4
20	22, 24	8
21	8	14

* Linkage map described by Molina-Luzón et al. [29]. ** Physical map described by Guerrero-Cózar et al. [7].

## Data Availability

https://www.ncbi.nlm.nih.gov/genbank/, Accessed on 7 February 2022.

## Supplementary Materials

The following supporting information can be downloaded at: https://www.mdpi.com/article/10.3390/ijms23105353/s1.
