# Supplementary material for: Integration of Maps Enables a Cytogenomics Analysis of the Complete Karyotype in Solea senegalensis
Source: Int J Mol Sci. 2022 May 11;23(10):5353. doi: 10.3390/ijms23105353 (PMC9140517; doi:10.3390/ijms23105353)
Supplement: Supplementary file 1 [file ijms-23-05353-s001.zip › Figure S40.pdf]

Figure S40.1

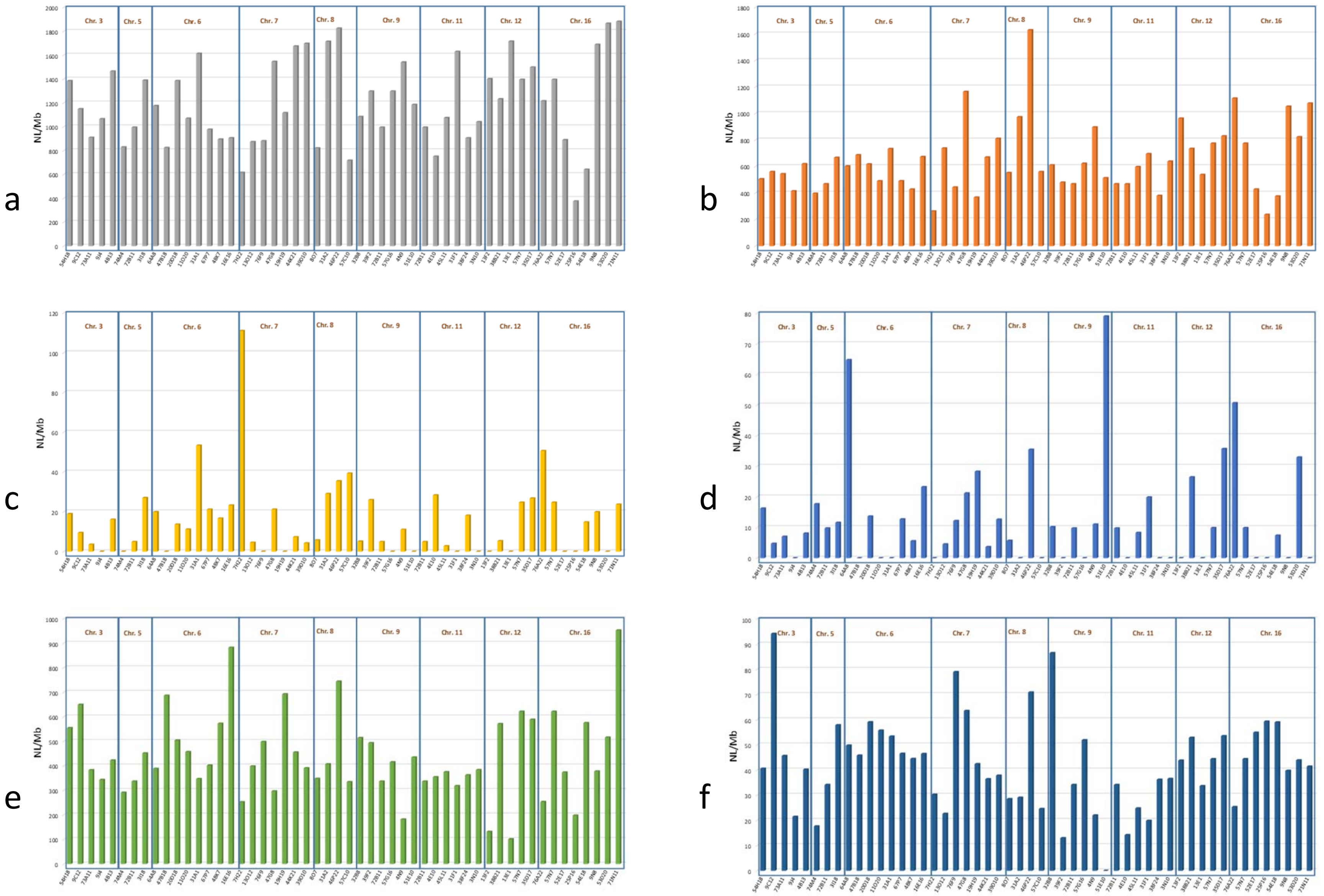

Figure S40.1: Number of *loci* per Mb (NL/Mb) of repeat elements in BACs from chromosomes 3,5-9, 11, 12 and 16 of *Solea senegalensis*. (**a**) DNA transposons, (**b**) retroelements, (**c**) small RNA, (d) satellites, (**e**) simple repeats, (**f**) low complexity.

Figure S40.2

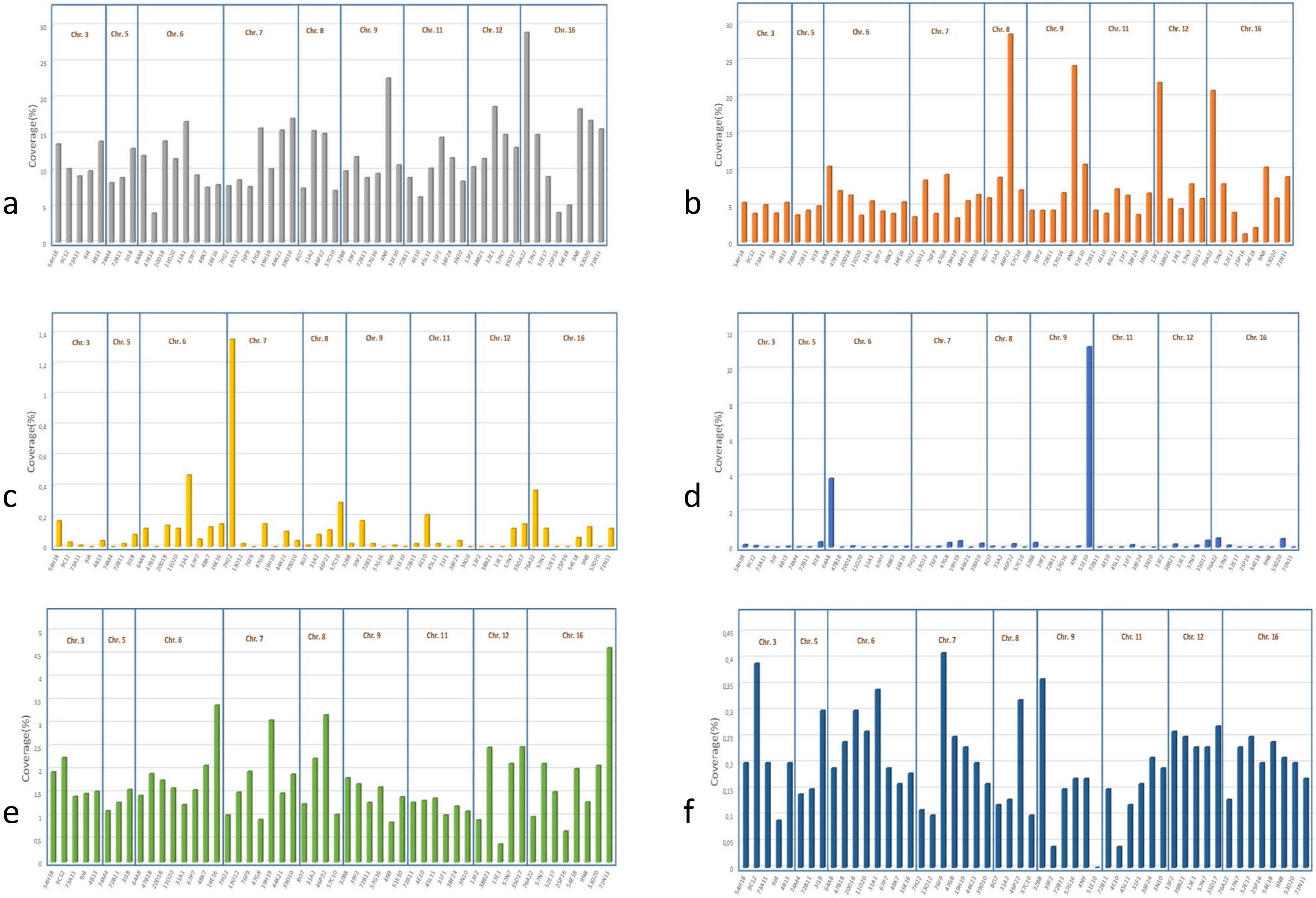

Figure S40.2. Coverage (%) of repeat elements in BACs from chromosomes 3,5-9, 11, 12 and 16 of *Solea senegalensis*. (**a**) DNA transposons, (**b**) retroelements, (**c**) small RNA, (d) satellites, (**e**) simple repeats, (**f**) low complexity.

Figure S40.3

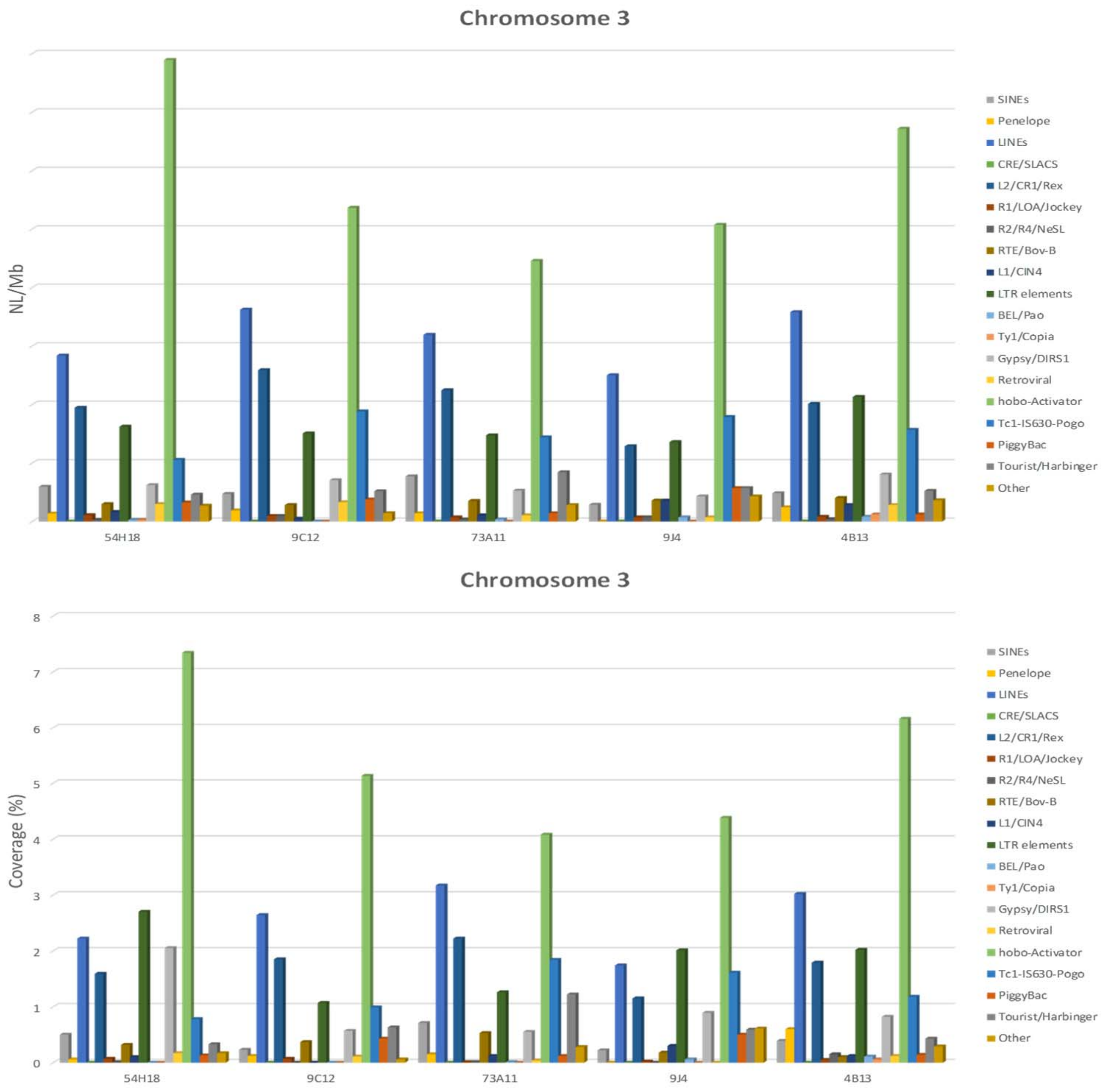

Figure S40.3. Abundance (measured as NL/Mb and Coverage) of TE families in BACs from chromosome 3 of *Solea senegalensis*.

Figure S40.4

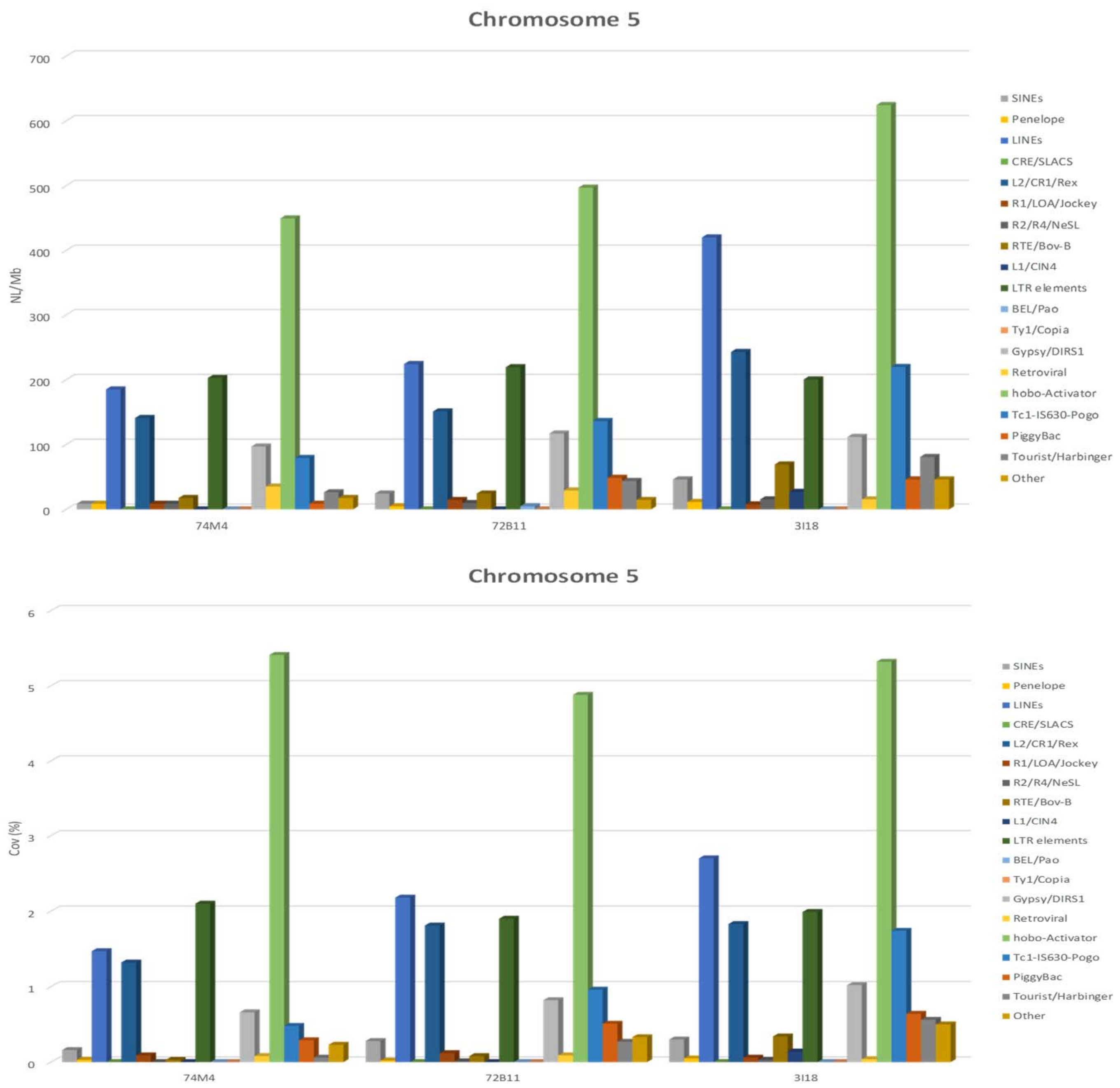

Figure S40.4. Abundance (measured as NL/Mb and Coverage) of TE families in BACs from chromosome 5 of *Solea senegalensis*.

Figure S40.5

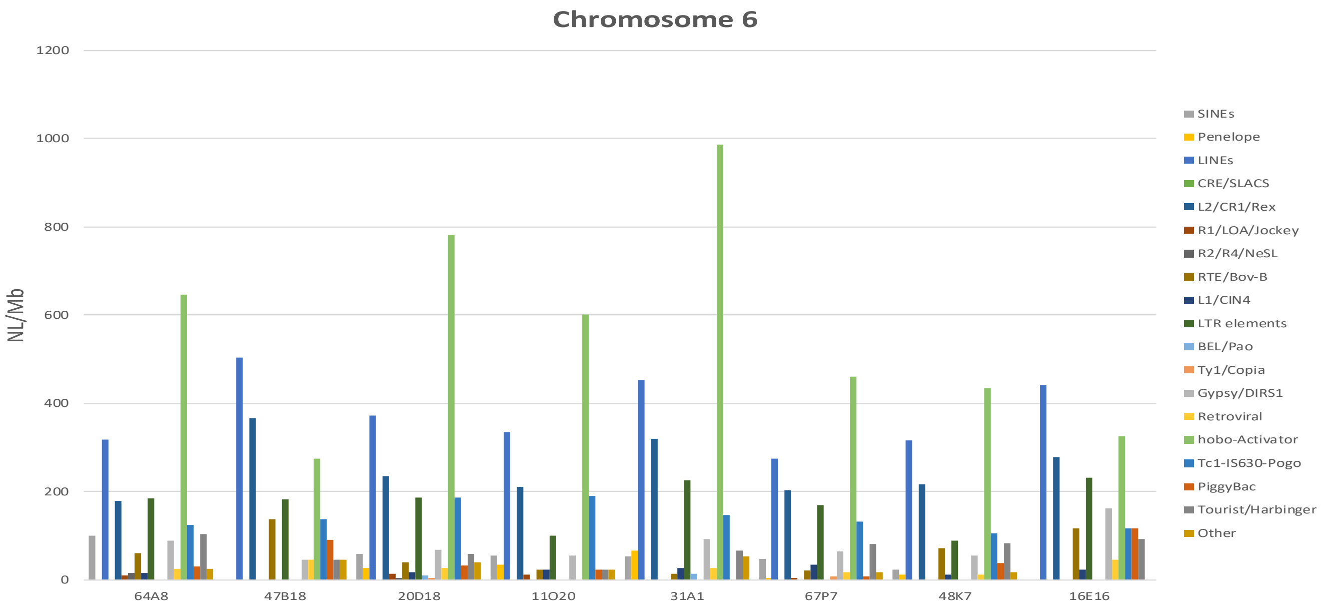

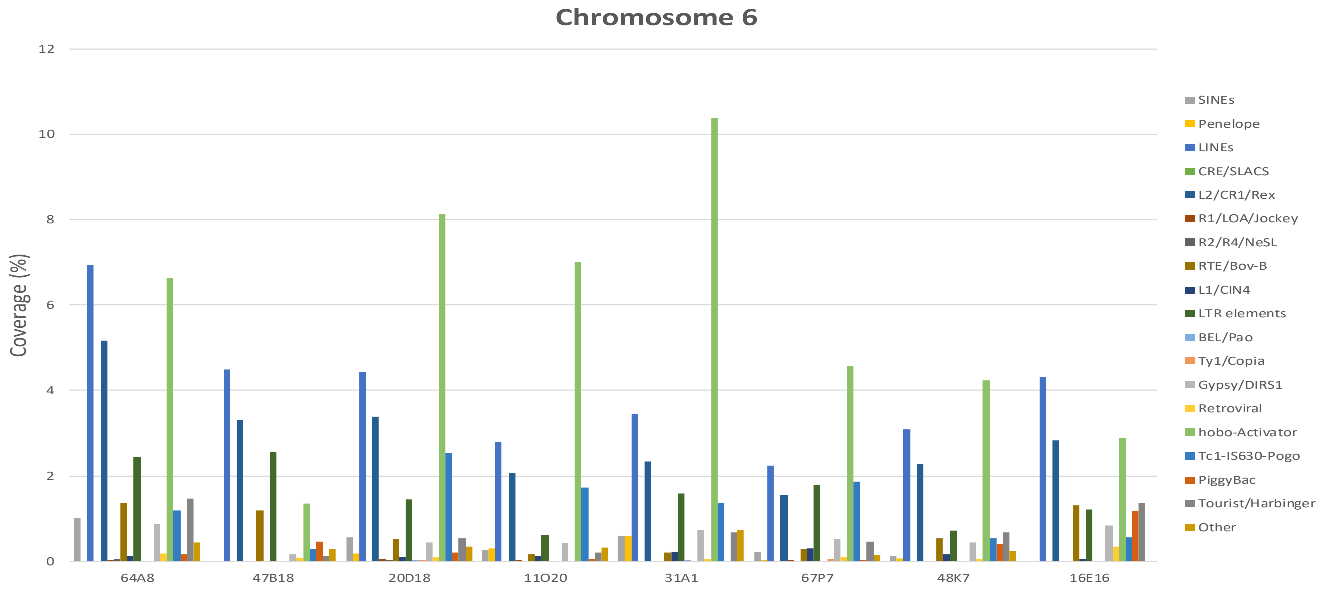

Figure S40.5. Abundance (measured as NL/Mb and Coverage) of TE families in BACs from chromosome 6 of *Solea senegalensis*.

Figure S40.6

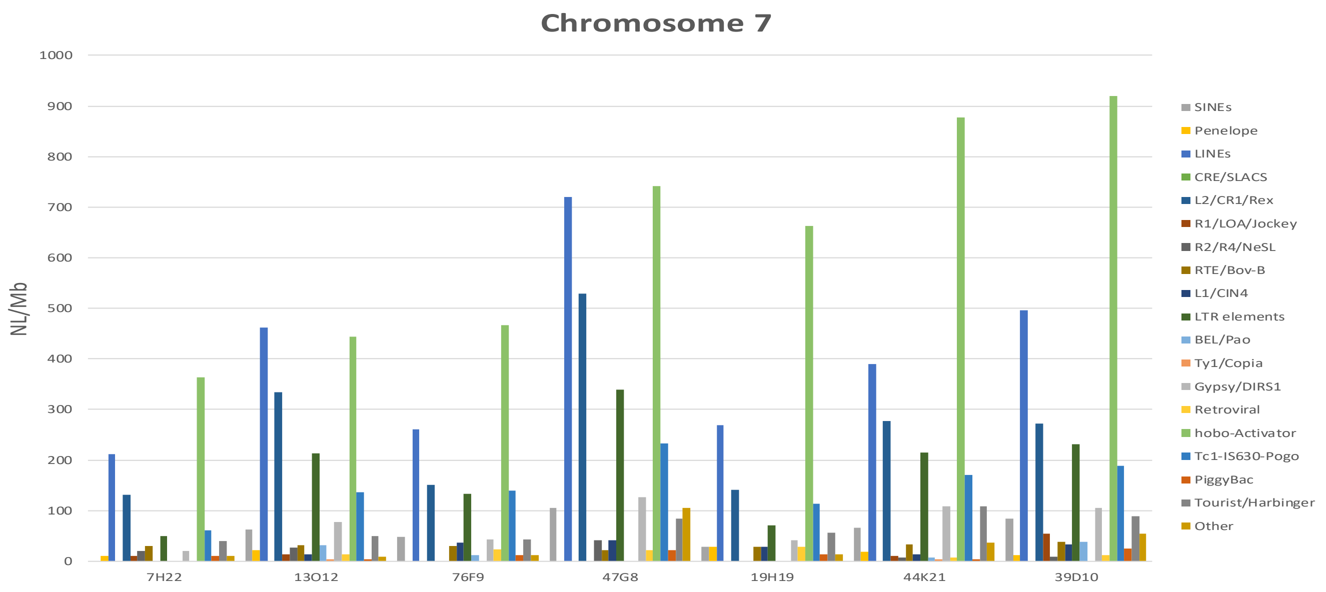

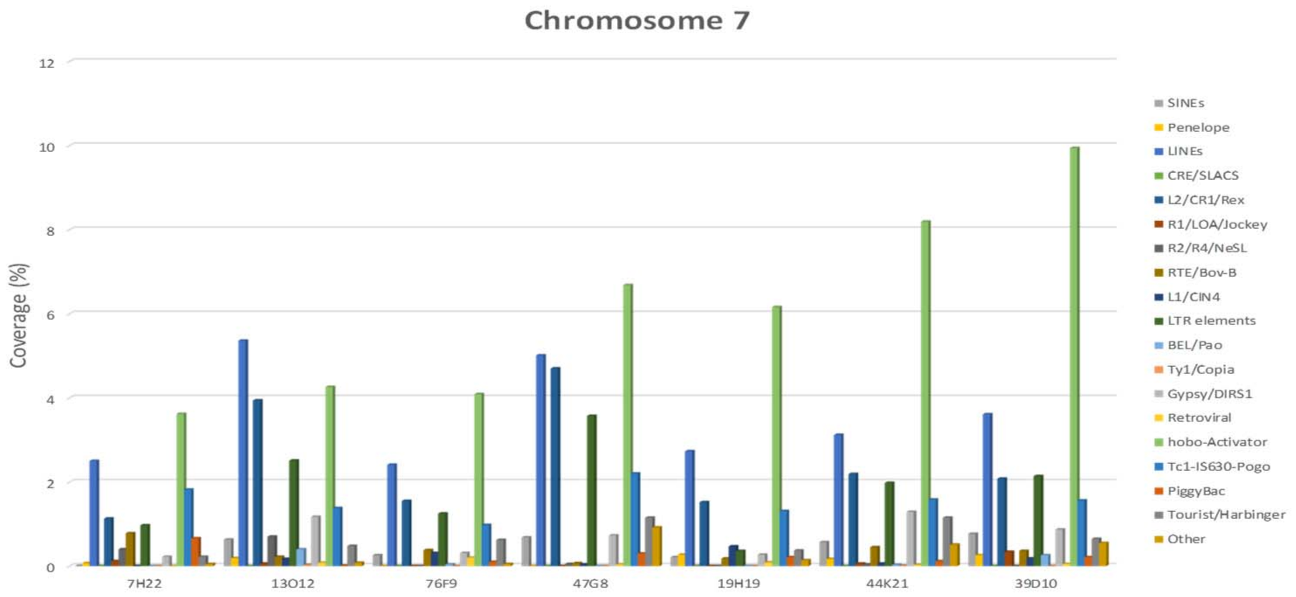

Figure S40.6. Abundance (measured as NL/Mb and Coverage) of TE families in BACs from chromosome 7 of *Solea senegalensis*.

Figure S40.7

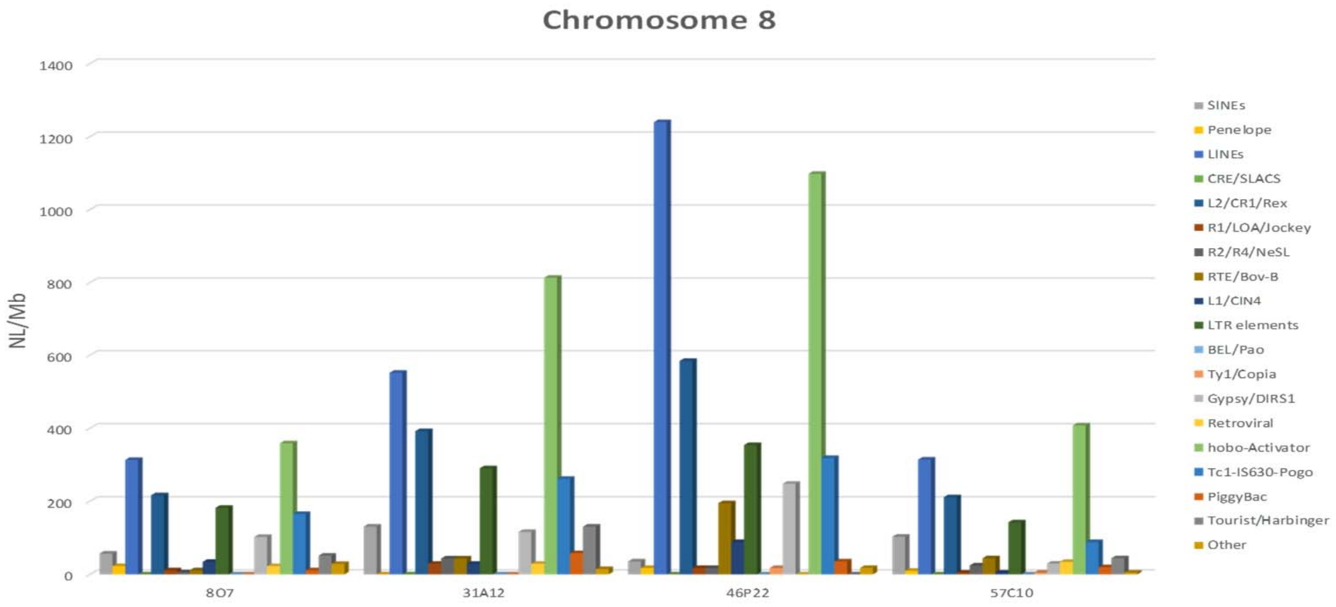

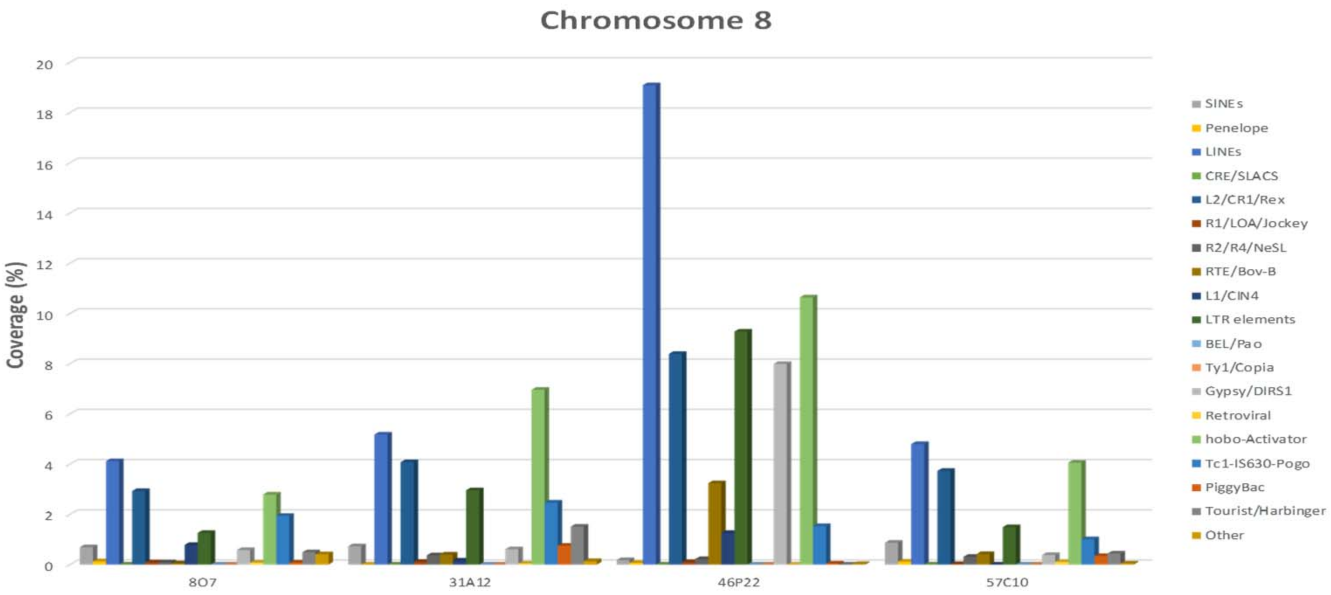

Figure S40.7. Abundance (measured as NL/Mb and Coverage) of TE families in BACs from chromosome 8 of *Solea senegalensis*.

Figure S40.8

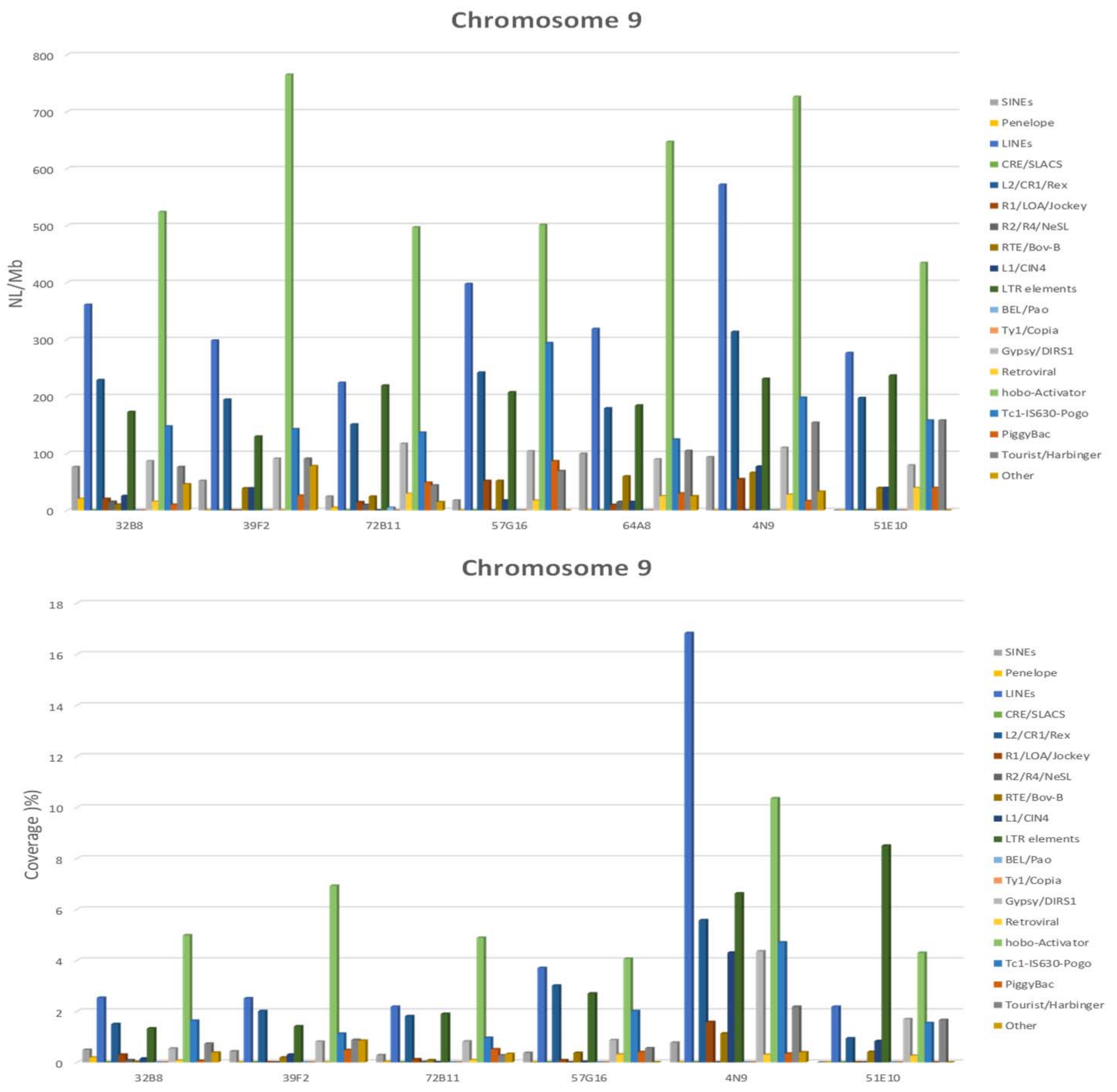

Figure S40.8. Abundance (measured as NL/Mb and Coverage) of TE families in BACs from chromosome 9 of *Solea senegalensis*.

Figure S40.9

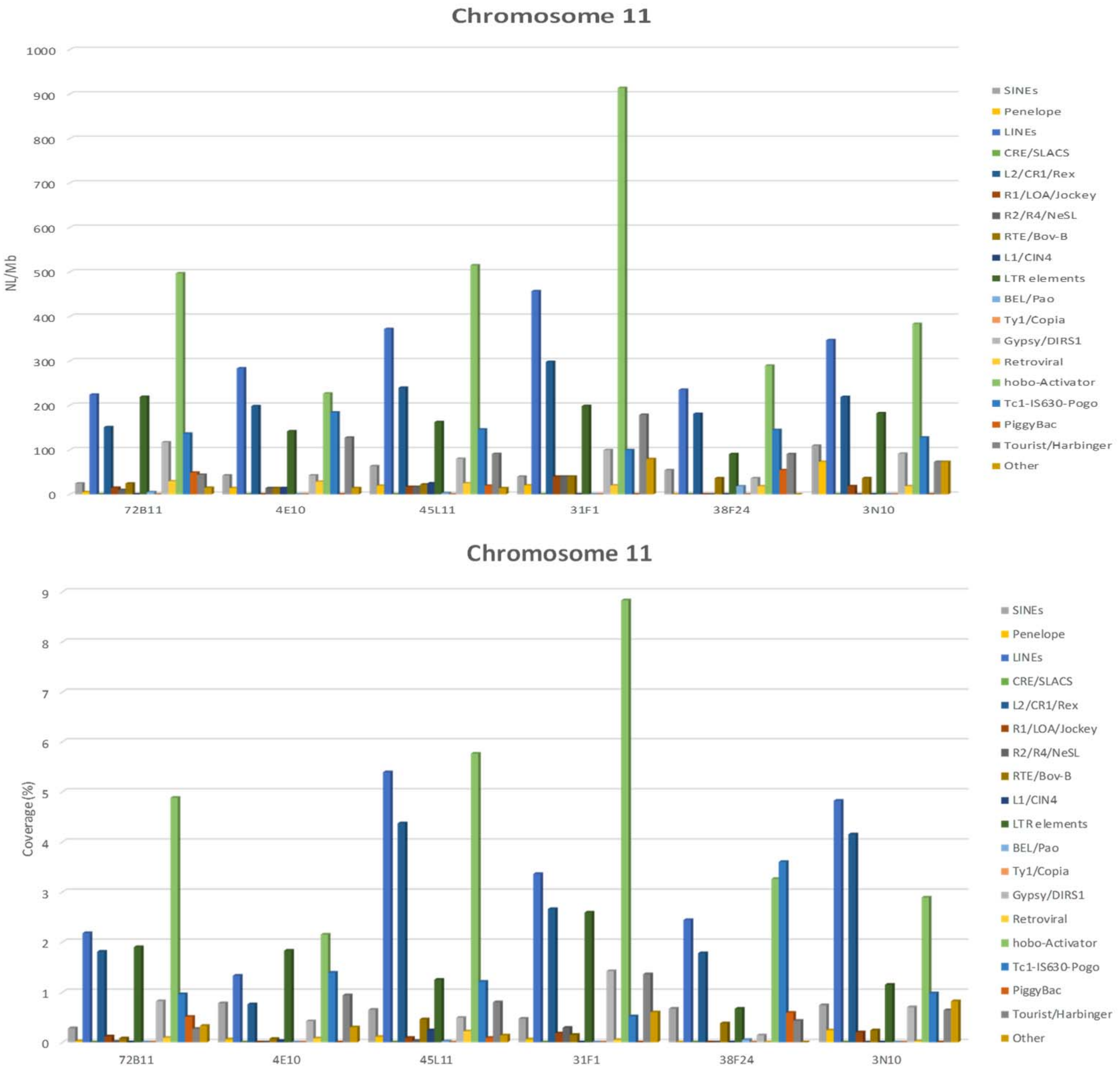

Figure S40.9. Abundance (measured as NL/Mb and Coverage) of TE families in BACs from chromosome 11 of *Solea senegalensis*.

Figure S40.10

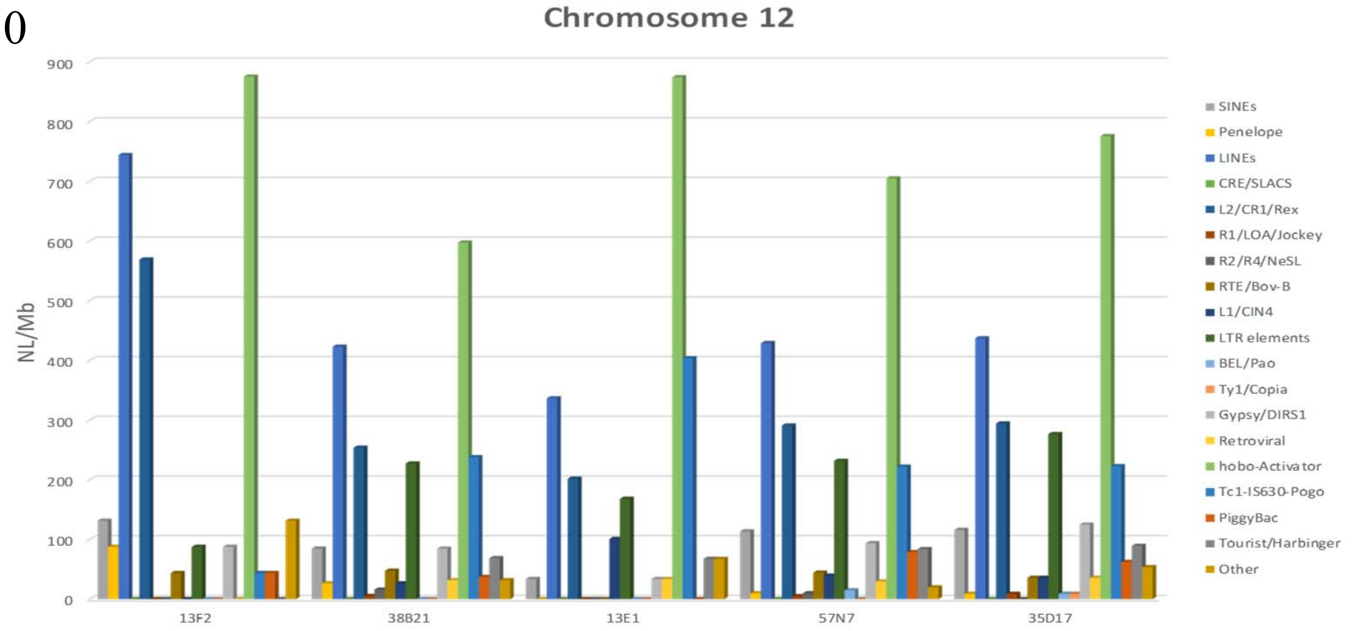

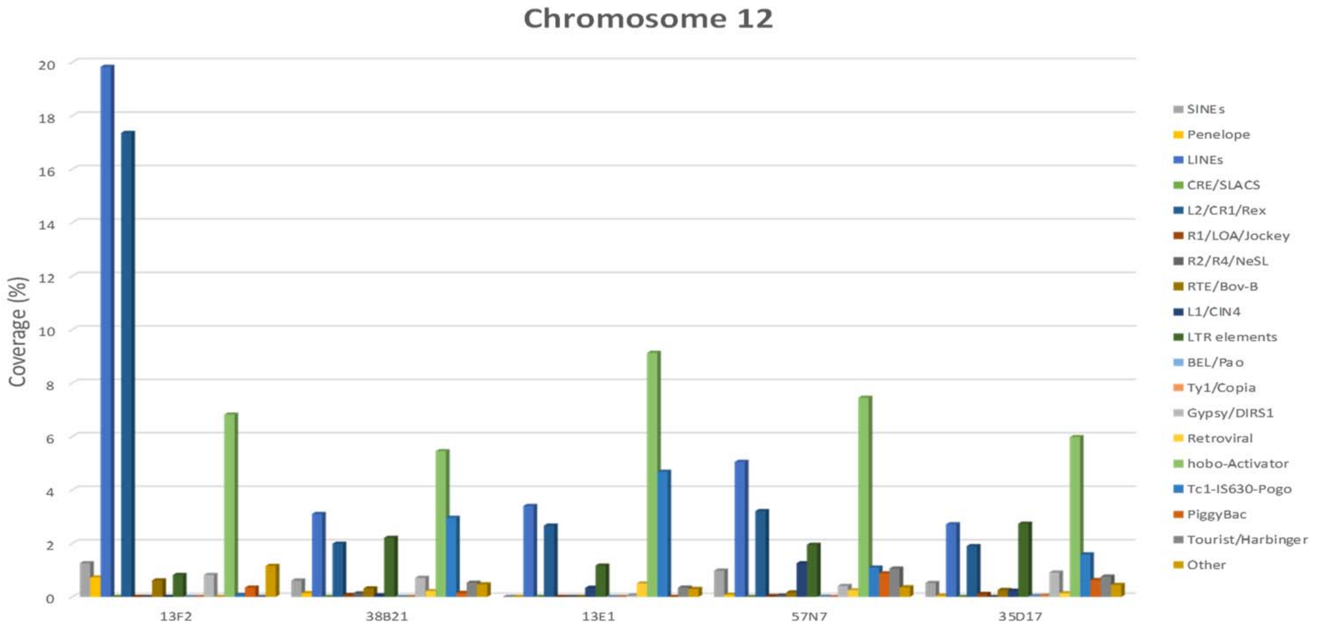

Figure S40.10. Abundance (measured as NL/Mb and Coverage) of TE families in BACs from chromosome 12 of *Solea senegalensis*.

Figure S40.11

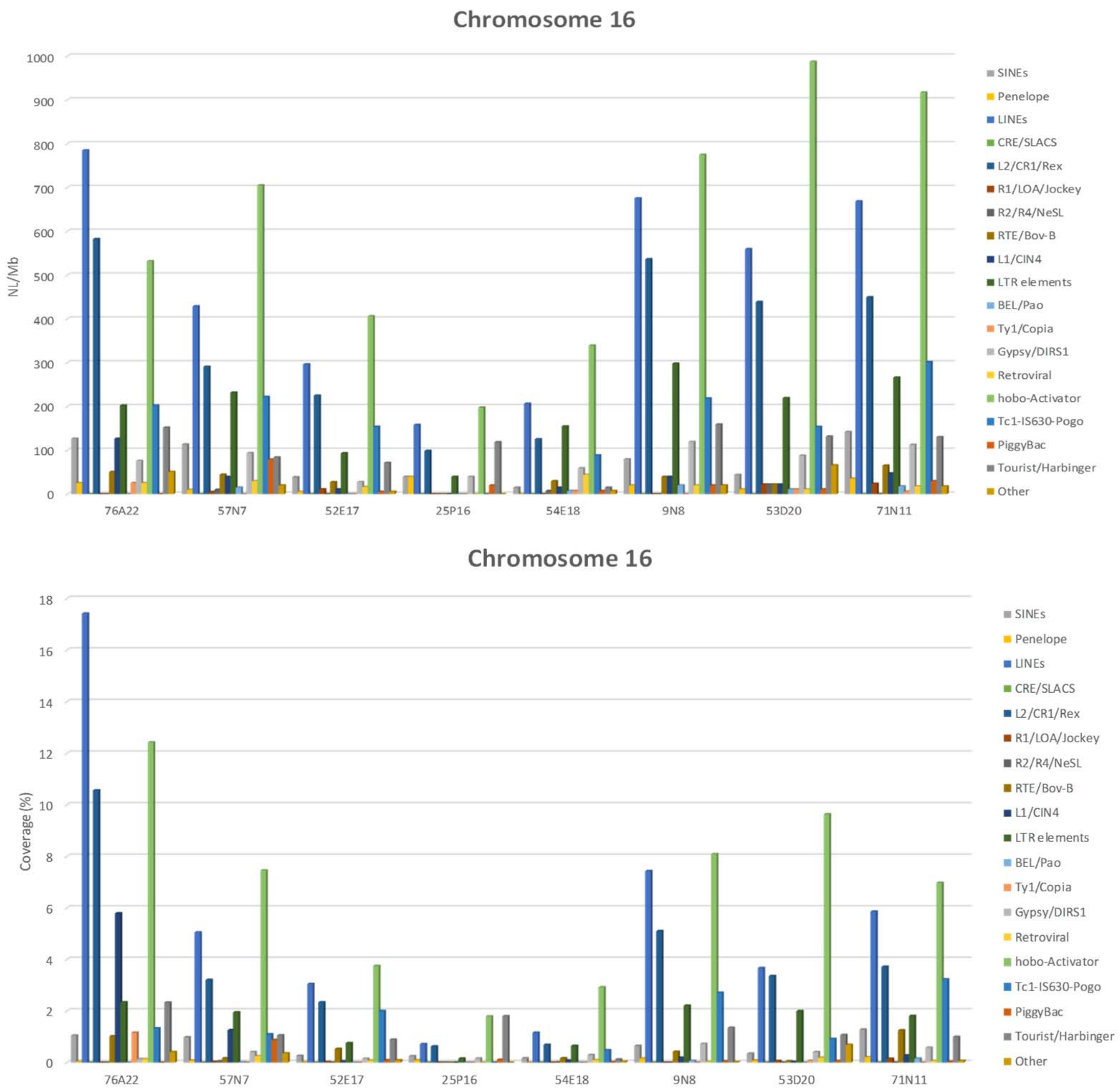

Figure S40.11. Abundance (measured as NL/Mb and Coverage) of TE families in BACs from chromosome 16 of *Solea senegalensis*.

Figure S40.12

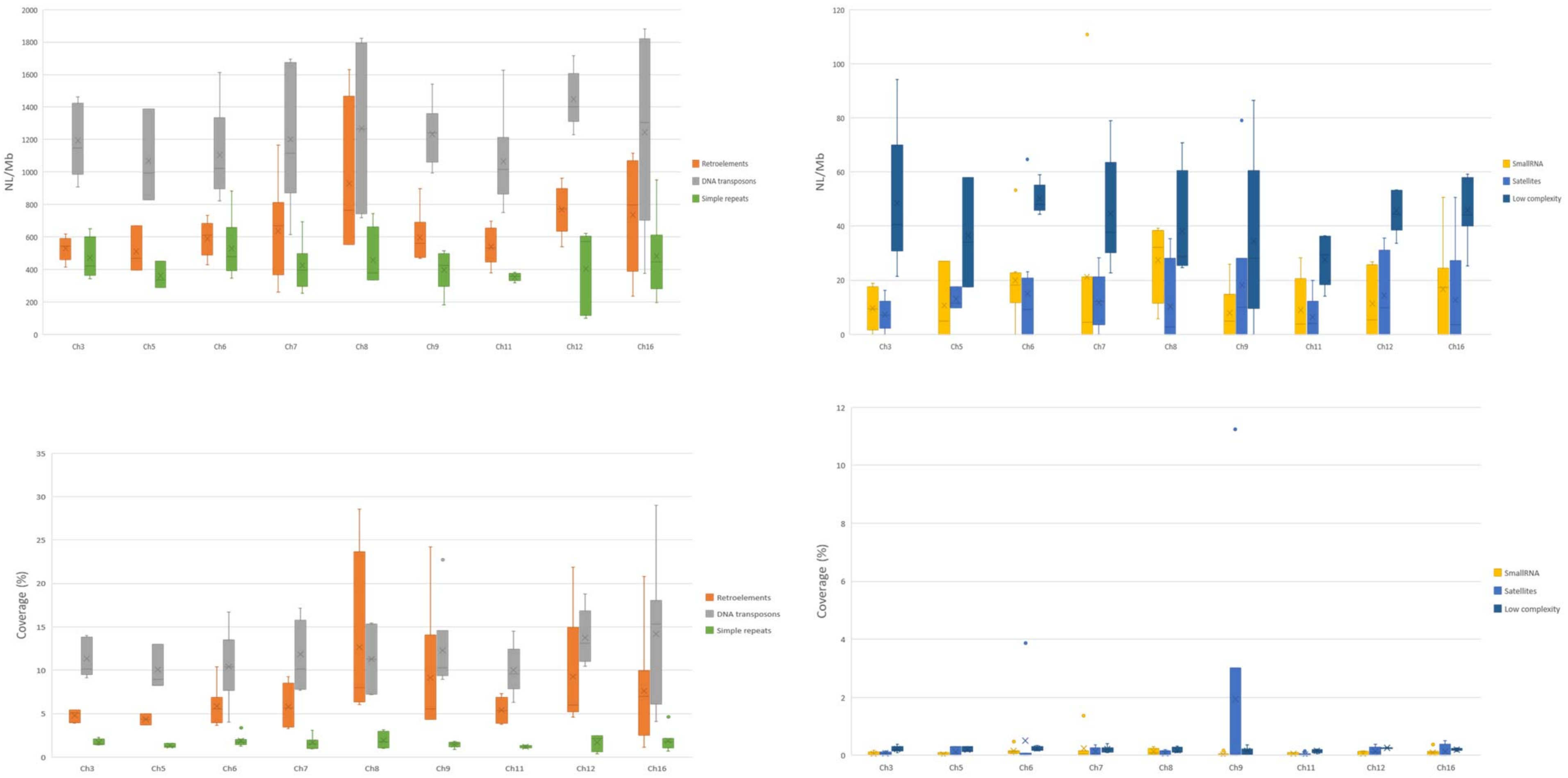

Figure S40.12. Boxplot summarizing the number of loci per Mb (NL/Mb) and coverage (%) of repeat elements in chromosomes 3,5-9, 11, 12 and 16 of *Solea senegalensis* inferred from clon BACs analysis.

Figure S40.13

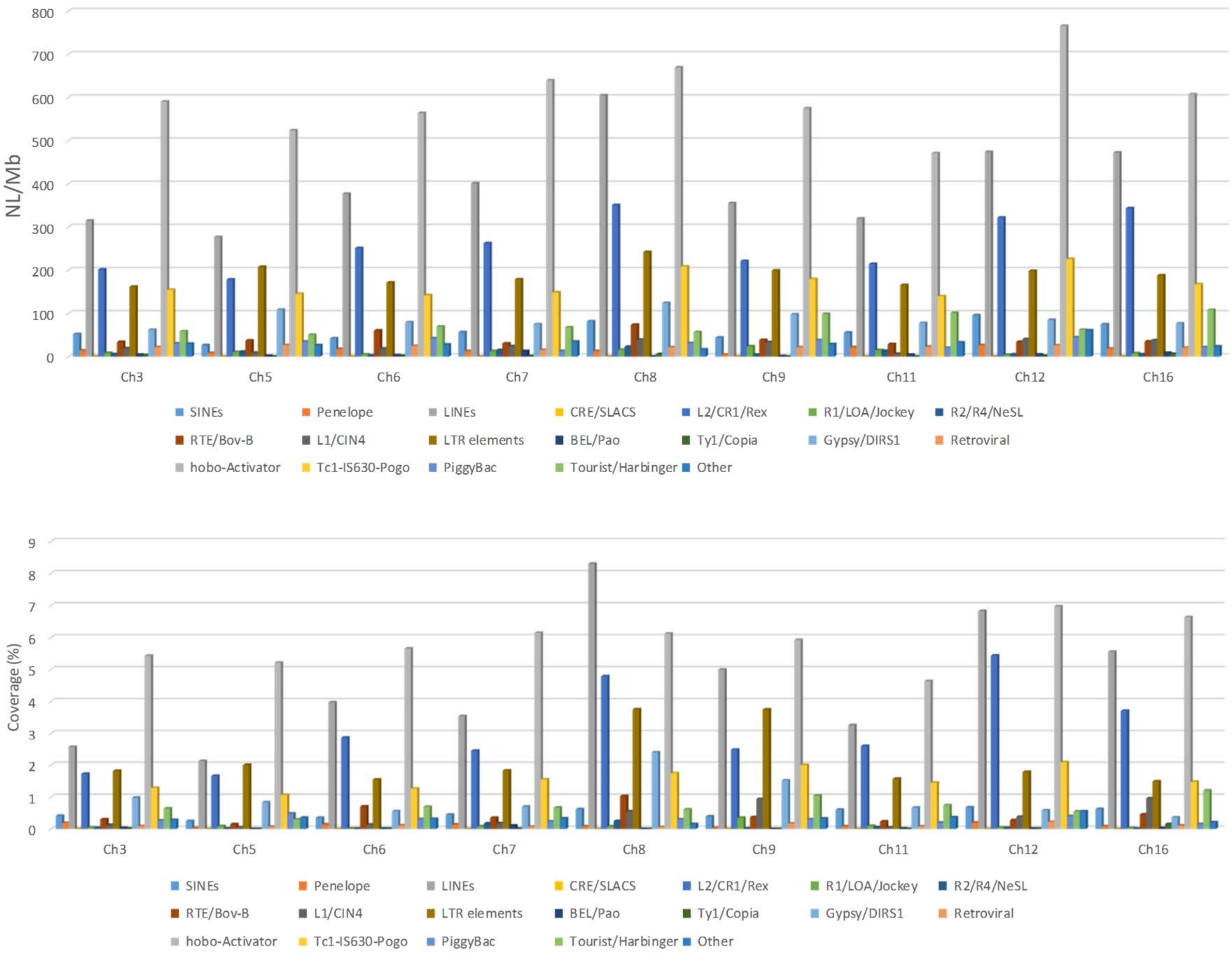

Figure S40.13. Average abundance (measured as NL/Mb and Coverage) of TE families in chromosomes 3, 5-9, 11-12 and 16 of *Solea senegalensis* inferred from BAC analysis.
