# Supplementary material for: Integration of Maps Enables a Cytogenomics Analysis of the Complete Karyotype in Solea senegalensis
Source: Int J Mol Sci. 2022 May 11;23(10):5353. doi: 10.3390/ijms23105353 (PMC9140517; doi:10.3390/ijms23105353)
Supplement: Supplementary file 1 [file ijms-23-05353-s001.zip › Supplementary Figures S1-39.pdf]

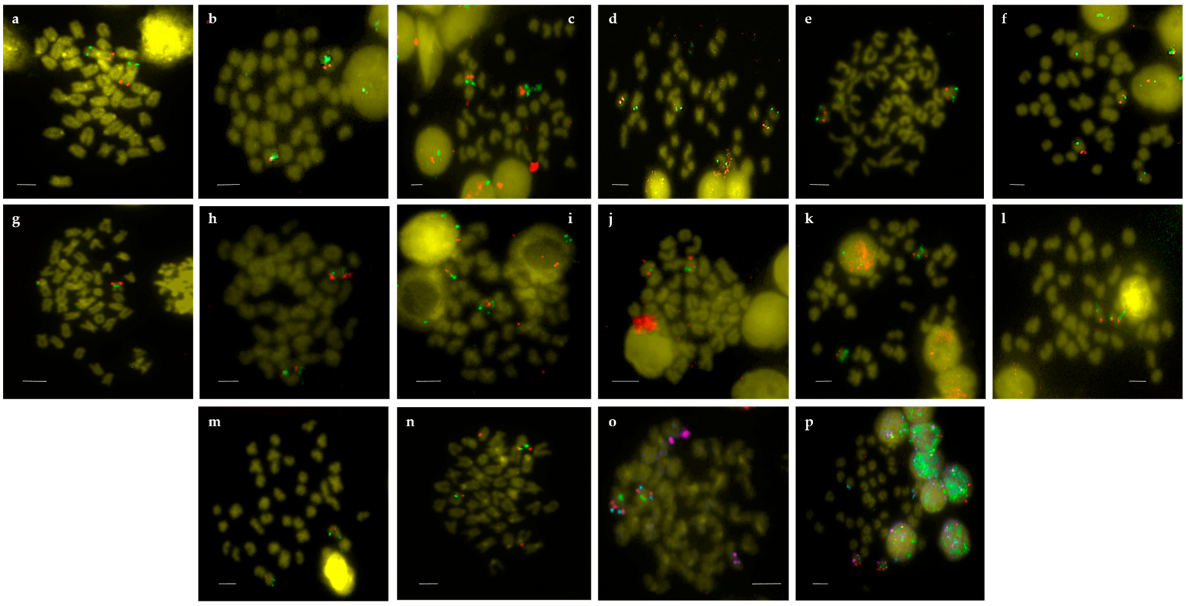

Figure S1. Results of double (a – n) and multicolor FISH (o and p) with BACs of *Solea senegalensis*. (a)10L10 (red)/67N4 (green) for chromosome 1, (b) 21I14 (red)/38N10 (green) for chromosome 2, (c) 67K3 (red)/12N15 (green) for chromosome 4, (d) 16E16 (green)/67P7 (red) for chromosome 6, (e) 7H22 (red)/47G8 (green) for chromosome 7, (f) 31A2 (red)/46P22 (green) for chromosome 8, (g) 15I19 (red)/57N7 (green) for chromosome 10, (h) 72B11 (red)/31F1 (green) for chromosome 11, (i) 35D17 (green)/38B21 (red) for chromosome 12, (j) 29D4 (green)/73A11 (red) for chromosome 14, (k) 36E3 (red)/44K21 (green) for chromosome 15, (l) 71N11 (green)/54E18 (red) for chromosome 16, (m) 12K16 (red)/38H3 (green) for chromosome 19, (n) 55B12 (red)/72O12 (green) for chromosome 21, (o) 42D4 (blue)/65E23 (red)/21I14 (green) for chromosome 2 and 54E18 (pink) located in chromosome 16, (p) 67K3 (green)/39G22 (pink)/36J2 (red) located in chromosome 4 and 54H18 (blue) for chromosome 3. Bar = 2 μm (refered to large metacentric chromosome 1). 6N4, 21I14, 67K3, 67P7, 47G8, 31A2, 57N7, 72B11, 38B21, 73A11, 44K21, 54E18, 38H3, 72O12, 42D4, 65E23, 39G22 and 54H18 are BACs containing microsatellites of the Molina-Luzón [29] linkage map.

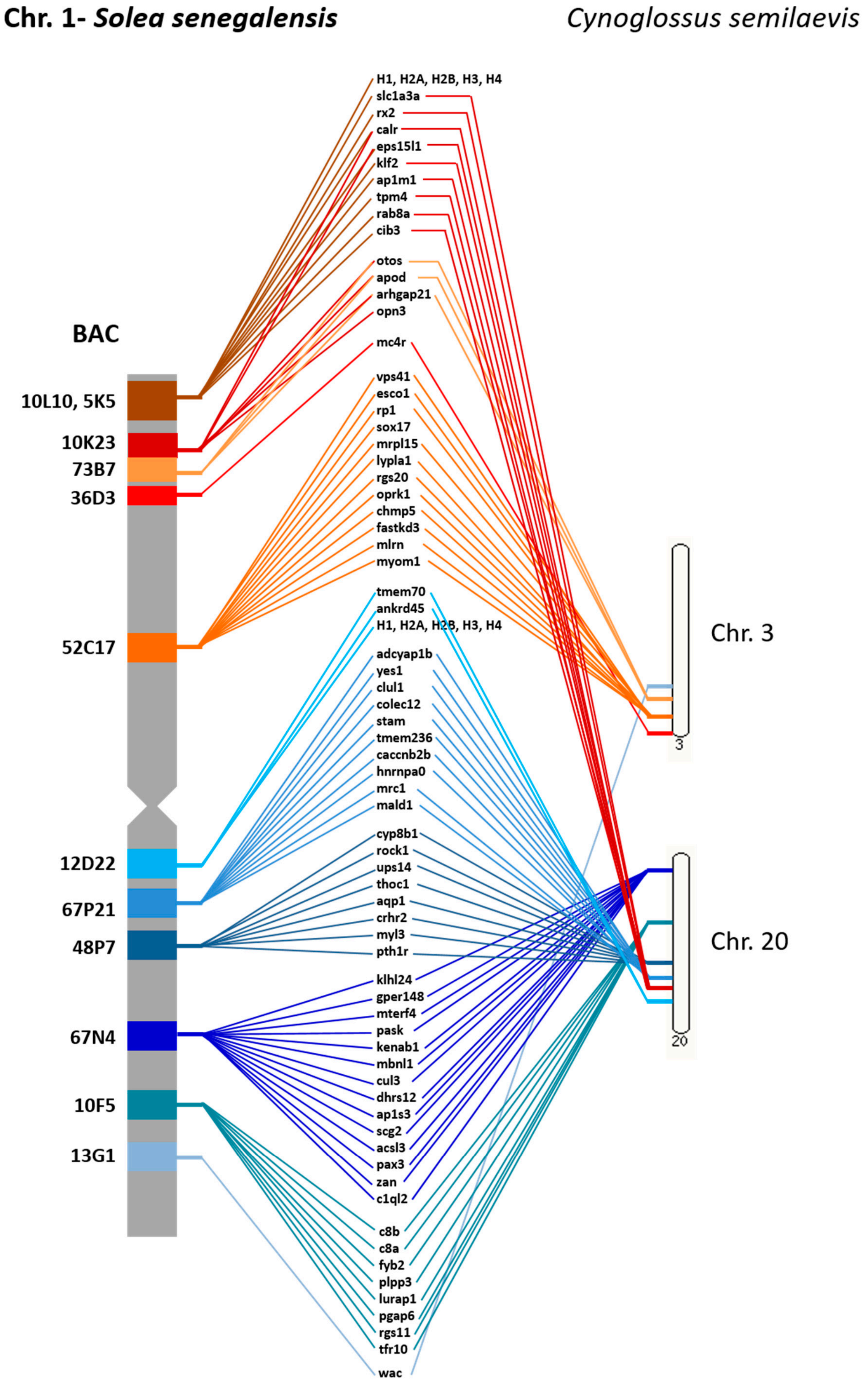

Chr. 1- Solea senegalensis
Cynoglossus semilaevis
BAC
10L10, 5K5
10K23
73B7
36D3
52C17
12D22
67P21
48P7
67N4
10F5
13G1
H1, H2A, H2B, H3, H4
slc1a3a
rx2
calr
eps15l1
klf2
ap1m1
tpm4
rab8a
cib3
otos
apod
arhgap21
opn3
mc4r
vps41
esco1
rp1
sox17
mrpl15
lypla1
rgs20
oprk1
chmp5
fastkd3
mlrn
myom1
tmem70
ankrd45
H1, H2A, H2B, H3, H4
adcyap1b
yes1
clul1
colec12
stam
tmem236
caccnb2b
hnrnpa0
mrc1
mald1
cyp8b1
rock1
ups14
thoc1
aqp1
crhr2
myl3
pth1r
klhl24
gper148
mterf4
pask
kenab1
mbnl1
cul3
dhrs12
ap1s3
scg2
acsl3
pax3
zan
c1ql2
c8b
c8a
fyb2
plpp3
lurap1
pgap6
rgs11
tfr10
wac
Chr. 3
3
Chr. 20
20

Figure S2. Syntenic map of the metacentric chromosome 1 of *Solea senegalensis* with respect to *Cynoglossus semilaevis*.

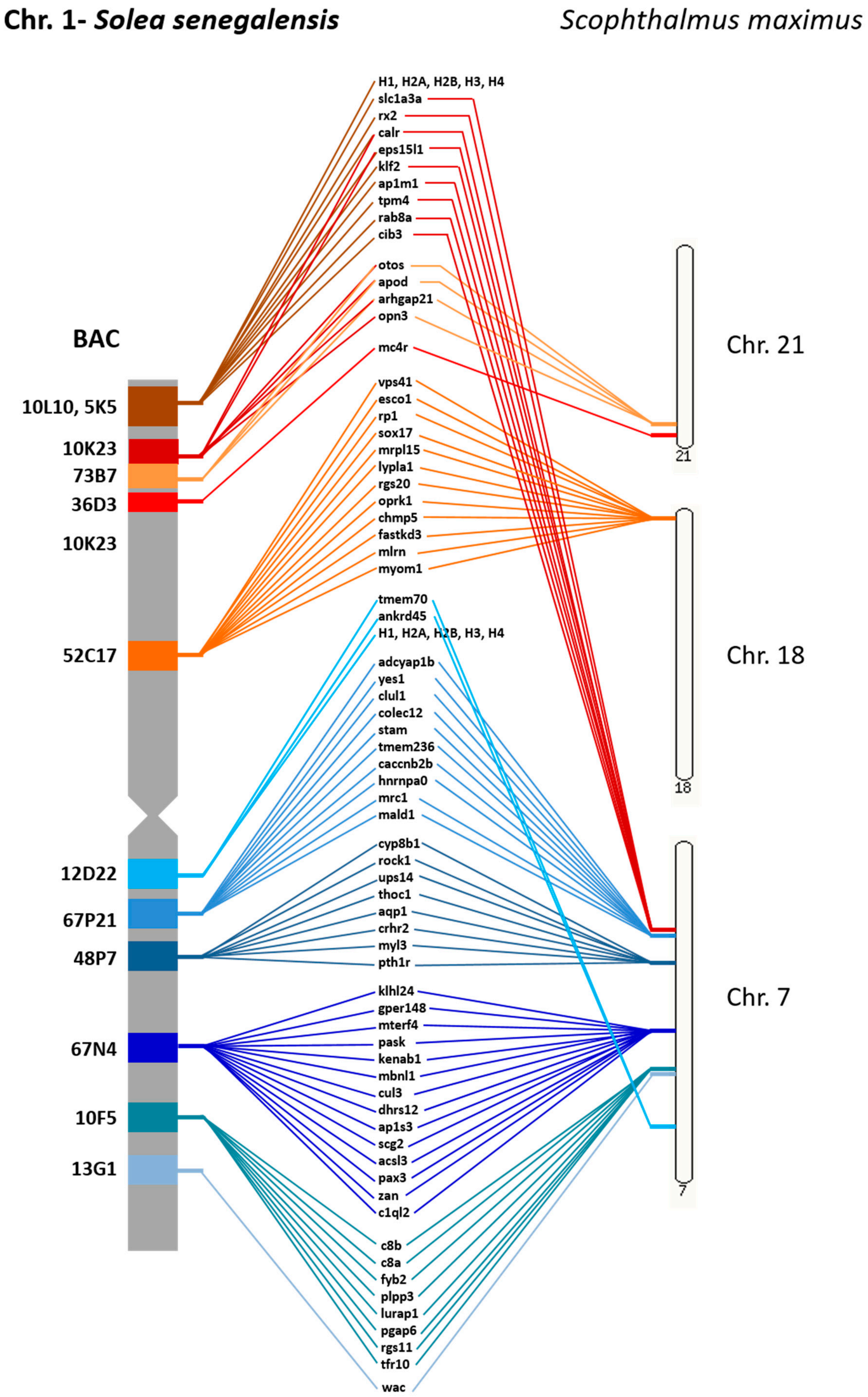

Chr. 1- *Solea senegalensis*
Scophthalmus maximus
BAC
10L10, 5K5
10K23
73B7
36D3
10K23
52C17
12D22
67P21
48P7
67N4
10F5
13G1
H1, H2A, H2B, H3, H4
slc1a3a
rx2
calr
eps15l1
klf2
ap1m1
tpm4
rab8a
cib3
otos
apod
arhgap21
opn3
mc4r
vps41
esco1
rp1
sox17
mrpl15
lypla1
rgs20
oprk1
chmp5
fastkd3
mlrn
myom1
tmem70
ankrd45
H1, H2A, H2B, H3, H4
adcyap1b
yes1
clul1
colec12
stam
tmem236
caccnb2b
hnrnpa0
mrc1
mald1
cyp8b1
rock1
ups14
thoc1
aqp1
crhr2
myl3
pth1r
klhl24
gper148
mterf4
pask
kenab1
mbnl1
cul3
dhrs12
ap1s3
scg2
acsl3
pax3
zan
c1ql2
c8b
c8a
fyb2
plpp3
lurap1
pgap6
rgs11
tfr10
wac
Chr. 21
21
Chr. 18
18
Chr. 7
7

Figure S3. Syntenic map of the metacentric chromosome 1 of *Solea senegalensis* with respect to *Scophthalmus maximus*.

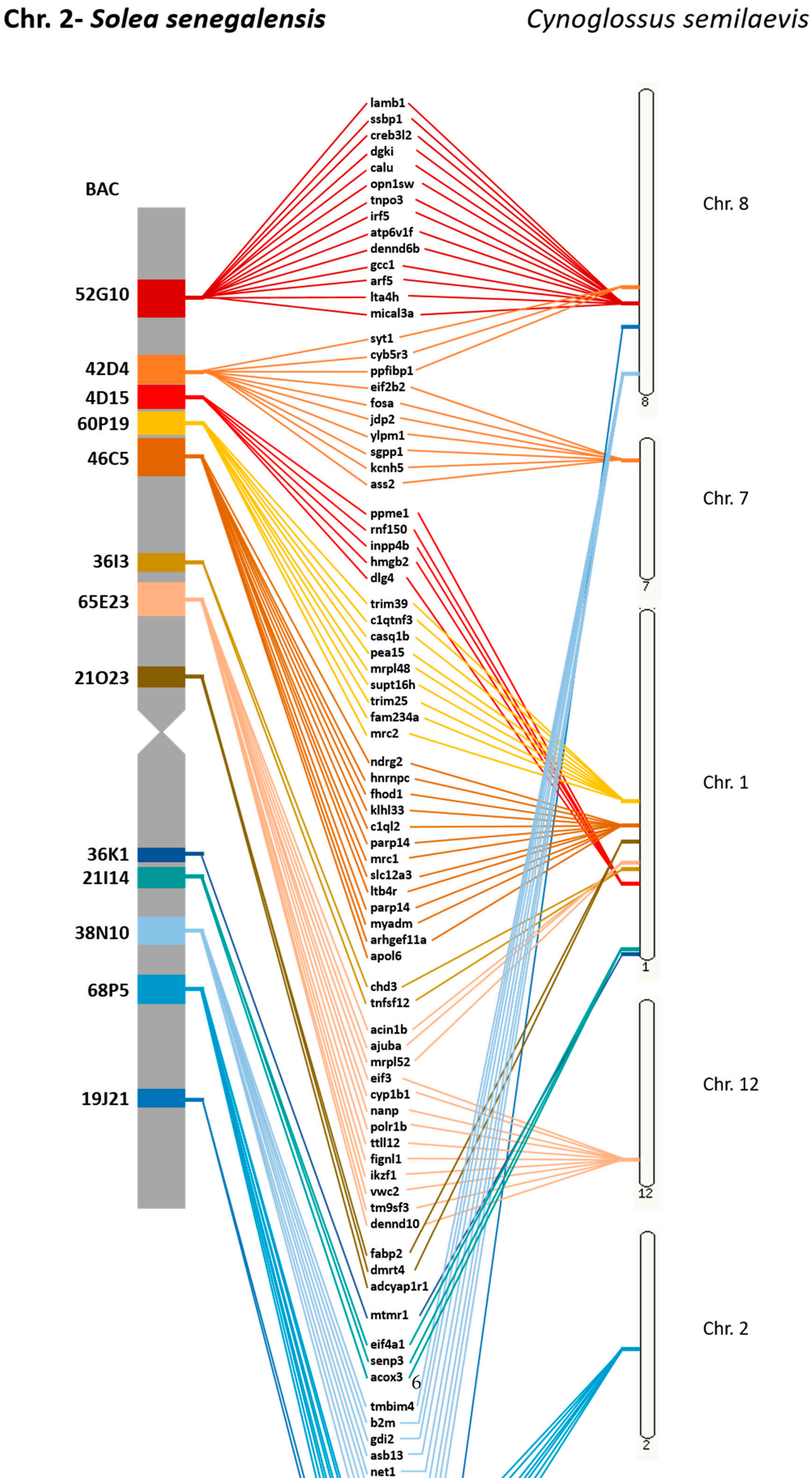
Chr. 2- Solea senegalensis
Cynoglossus semilaevis
BAC
52G10
42D4
4D15
60P19
46C5
36I3
65E23
21O23
36K1
21I14
38N10
68P5
19J21
lamb1
ssbp1
creb3l2
dgki
calu
opn1sw
tnpo3
irf5
atp6v1f
dennd6b
gcc1
arf5
lta4h
mical3a
syt1
cyb5r3
ppfibp1
eif2b2
fosa
jdp2
ylpm1
sgpp1
kcnh5
ass2
ppme1
rnf150
inpp4b
hmgb2
dlg4
trim39
c1qtnf3
casq1b
pea15
mrpl48
supt16h
trim25
fam234a
mrc2
ndrg2
hnrnpc
fhod1
klhl33
c1ql2
parp14
mrc1
slc12a3
ltb4r
parp14
myadm
arhgef11a
apol6
chd3
tnfsf12
acin1b
ajuba
mrpl52
eif3
cyp1b1
nanp
polr1b
ttll12
fignl1
ikzf1
vwc2
tm9sf3
dennd10
fabp2
dmrt4
adcyap1r1
mtmr1
eif4a1
senp3
acox3
tmbim4
b2m
gdi2
asb13
net1
Chr. 8
8
Chr. 7
7
Chr. 1
1
Chr. 12
12
Chr. 2
2

Figure S4. Syntenic map of the metacentric chromosome 2 of *Solea senegalensis* with respect to *Cynoglossus semilaevis*.

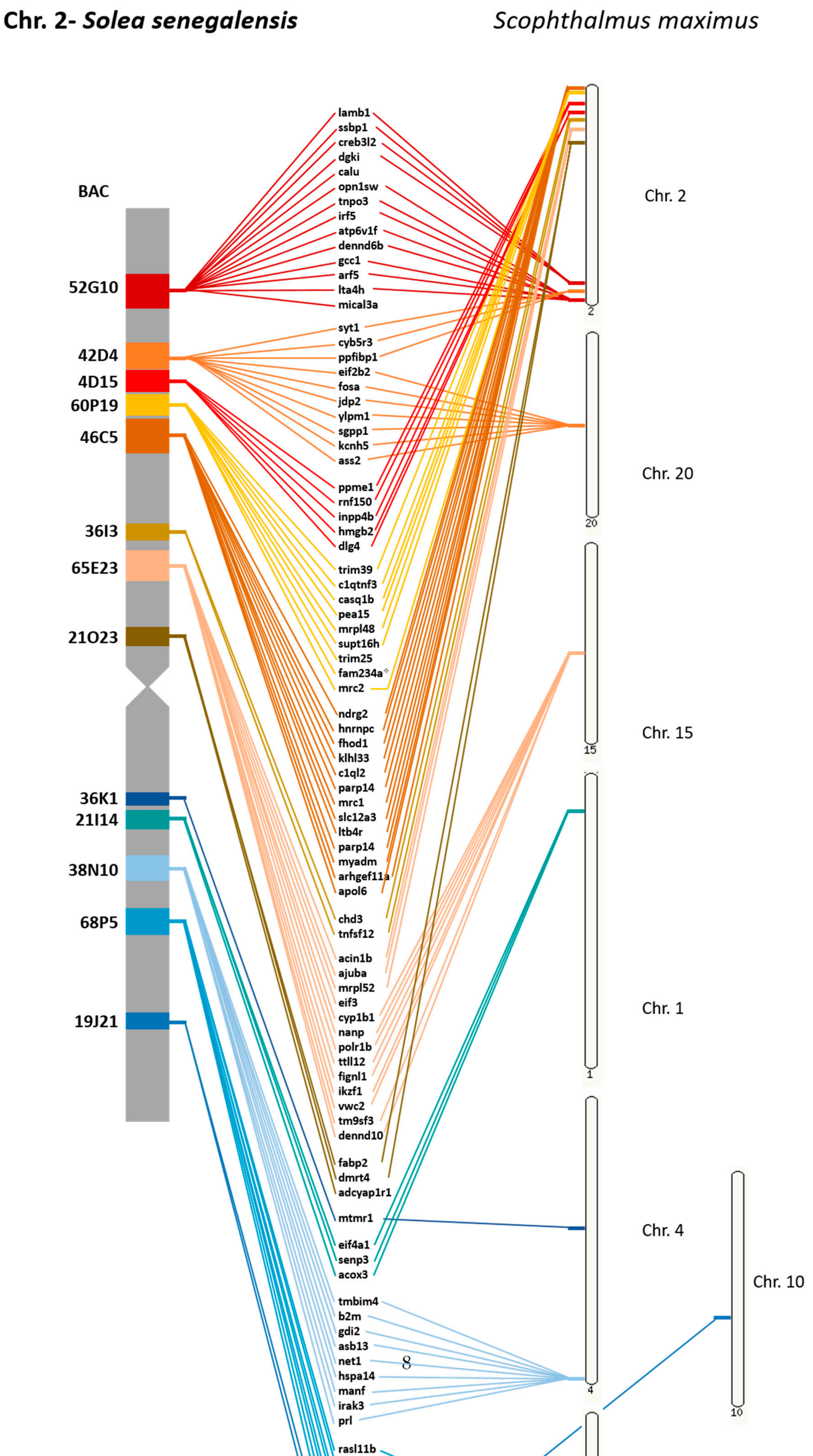
Chr. 2- Solea senegalensis
Scophthalmus maximus
BAC
52G10
42D4
4D15
60P19
46C5
36I3
65E23
21O23
36K1
21I14
38N10
68P5
19J21
lamb1
ssbp1
creb3l2
dgki
calu
opn1sw
tnpo3
irf5
atp6v1f
dennd6b
gcc1
arf5
lta4h
mical3a
syt1
cyb5r3
ppfibp1
eif2b2
fosa
jdp2
ylpm1
sgpp1
kcnh5
ass2
ppme1
rnf150
inpp4b
hmgb2
dlg4
trim39
c1qtnf3
casq1b
pea15
mrpl48
supt16h
trim25
fam234a
mrc2
ndrg2
hnrnpc
fhod1
klhl33
c1ql2
parp14
mrc1
slc12a3
ltb4r
parp14
myadm
arhgef11a
apol6
chd3
tnfsf12
acin1b
ajuba
mrpl52
eif3
cyp1b1
nanp
polr1b
ttll12
fignl1
ikzf1
vwc2
tm9sf3
dennd10
fabp2
dmrt4
adcyap1r1
mtmr1
eif4a1
senp3
acox3
tmbim4
b2m
gdi2
asb13
net1
hspa14
manf
irak3
prl
rasl11b
Chr. 2
Chr. 20
Chr. 15
Chr. 1
Chr. 4
Chr. 10
2
20
15
1
4
10

Figure S5. Syntenic map of the metacentric chromosome 2 of *Solea senegalensis* with respect to *Scophthalmus maximus*. Legend symbols: ✧Gene found in chromosome 18 of *S. maximus*. †Gene found in chromosome 2 of *S. maximus*. ✠Gene found in chromosome 19 of *S. maximus*.

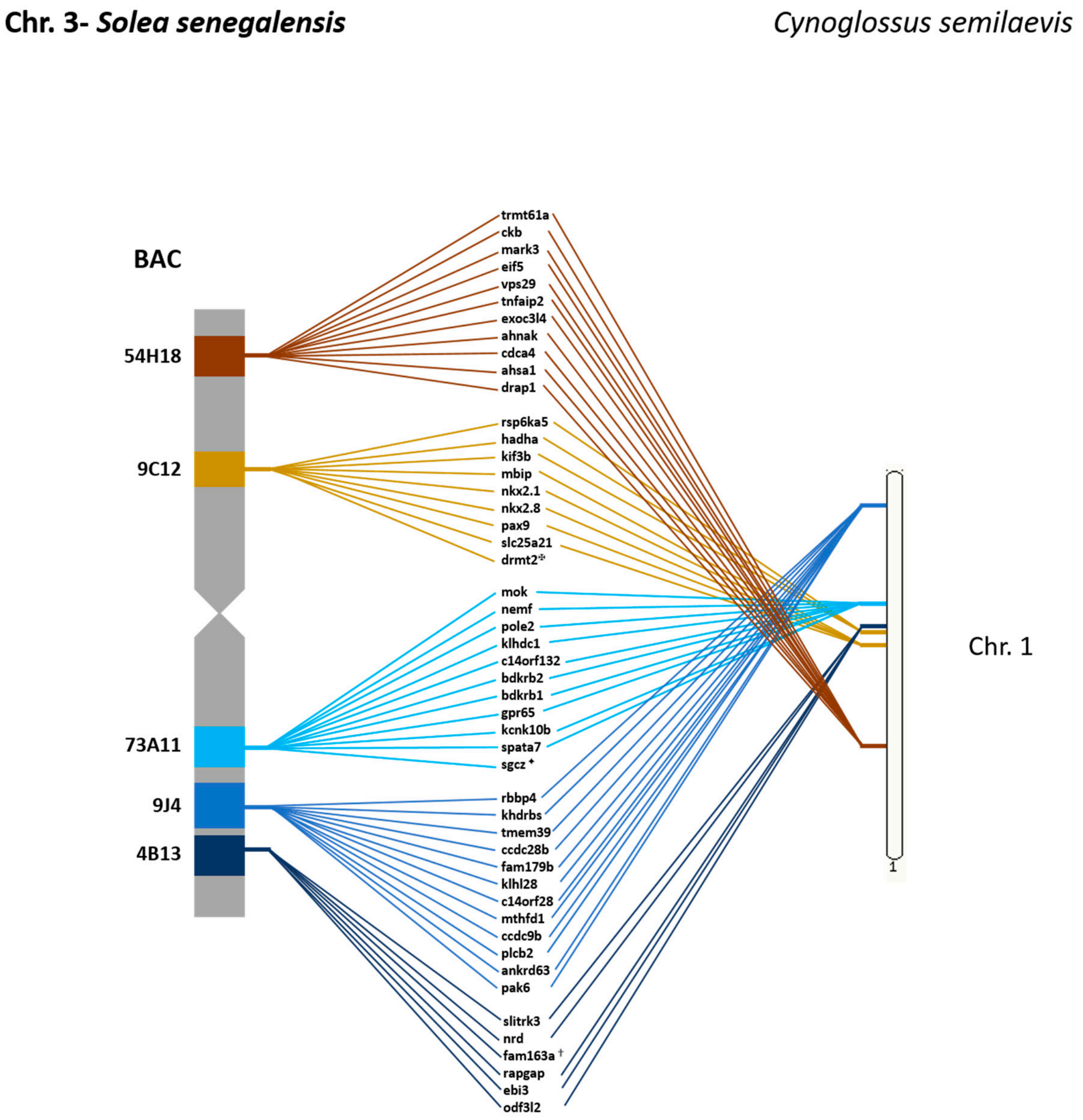

Figure S6. Syntenic map of the metacentric chromosome 3 of *Solea senegalensis* with respect to *Cynoglossus semilaevis*. Legend symbols: ✠Gene found in chromosome Z of *C. semilaevis*. ✦Gene found in chromosome 9 of *C. semilaevis* †Gene found in chromosome 2 of *C. semilaevis*.

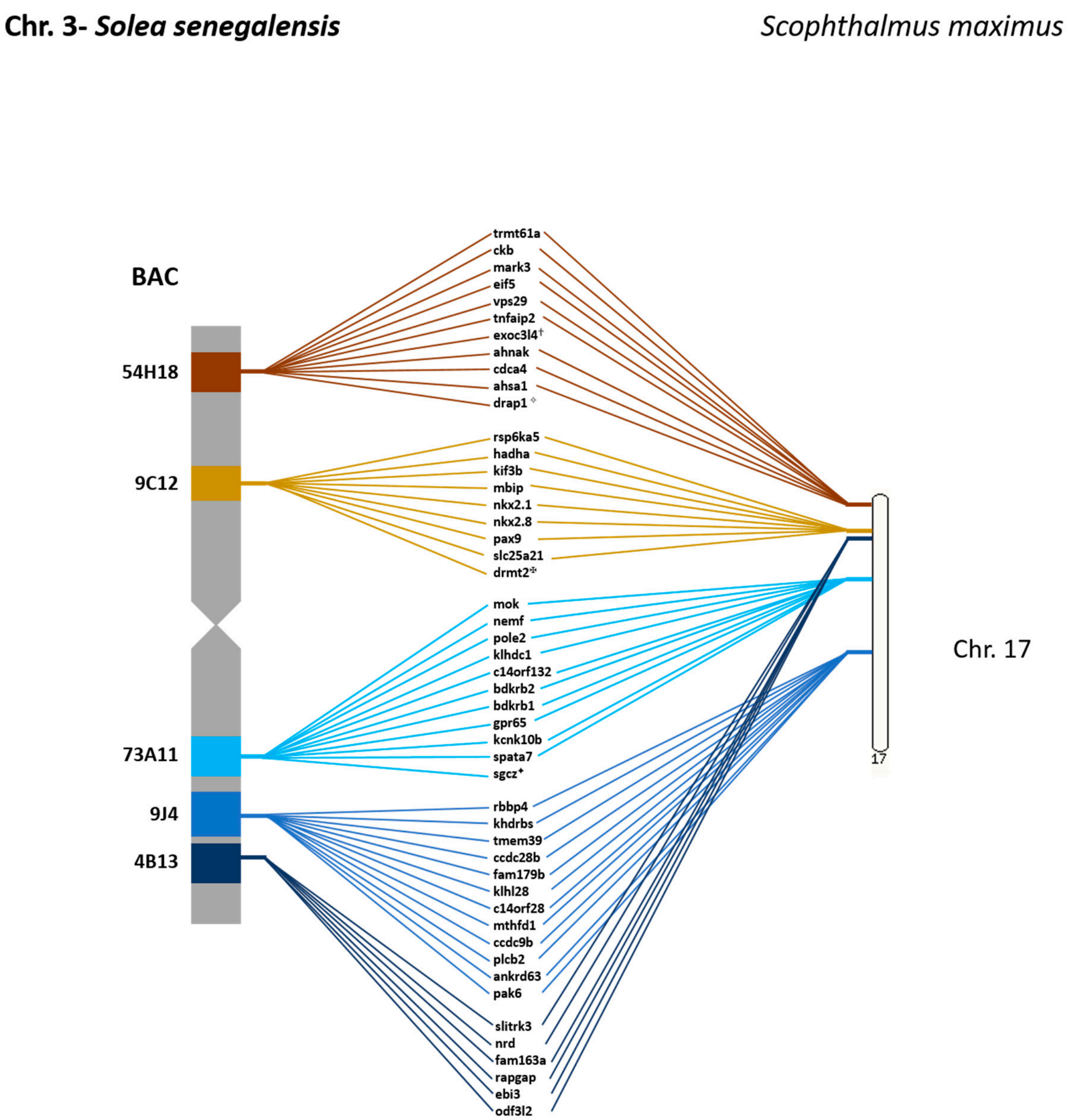

Figure S7. Syntenic map of the metacentric chromosome 3 of *Solea senegalensis* with respect to *Scophthalmus maximus*. Legend symbols: †Gene found in chromosome 3 of *S. maximus* in different position. ✧Gene found in chromosome 4 of *S. maximus*. ✠Gene found in chromosome 9 of *S. maximus*. ✦Genes found in chromosome 8 of *S. maximus*.

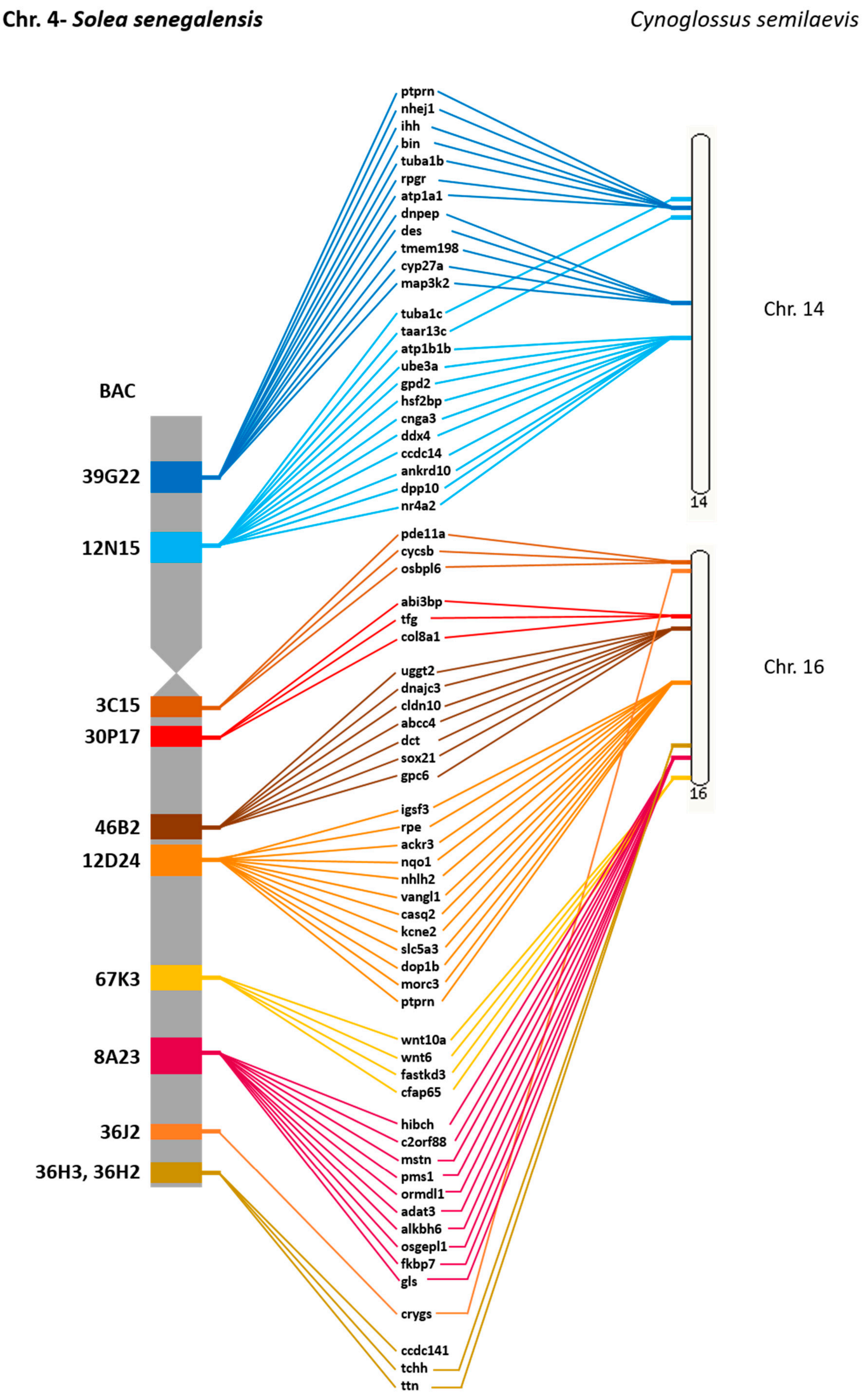
Chr. 4- Solea senegalensis
Cynoglossus semilaevis
BAC
39G22
12N15
3C15
30P17
46B2
12D24
67K3
8A23
36J2
36H3, 36H2
ptprn
nhej1
ihh
bin
tuba1b
rpgr
atp1a1
dnpep
des
tmem198
cyp27a
map3k2
tuba1c
taar13c
atp1b1b
ube3a
gpd2
hsf2bp
cnga3
ddx4
ccdc14
ankrd10
dpp10
nr4a2
pde11a
cycsb
osbpl6
abi3bp
tfg
col8a1
uggt2
dnajc3
cldn10
abcc4
dct
sox21
gpc6
igsf3
rpe
ackr3
nqo1
nhlh2
vangl1
casq2
kcne2
slc5a3
dop1b
morc3
ptprn
wnt10a
wnt6
fastkd3
cfap65
hibch
c2orf88
mstn
pms1
ormdl1
adat3
alkbh6
osgepl1
fkbp7
gls
crygs
ccdc141
tchh
ttn
14
Chr. 14
16
Chr. 16

Figure S8. Syntenic map of the submetacentric chromosome 4 of *Solea senegalensis* with respect to *Cynoglossus semilaevis*.

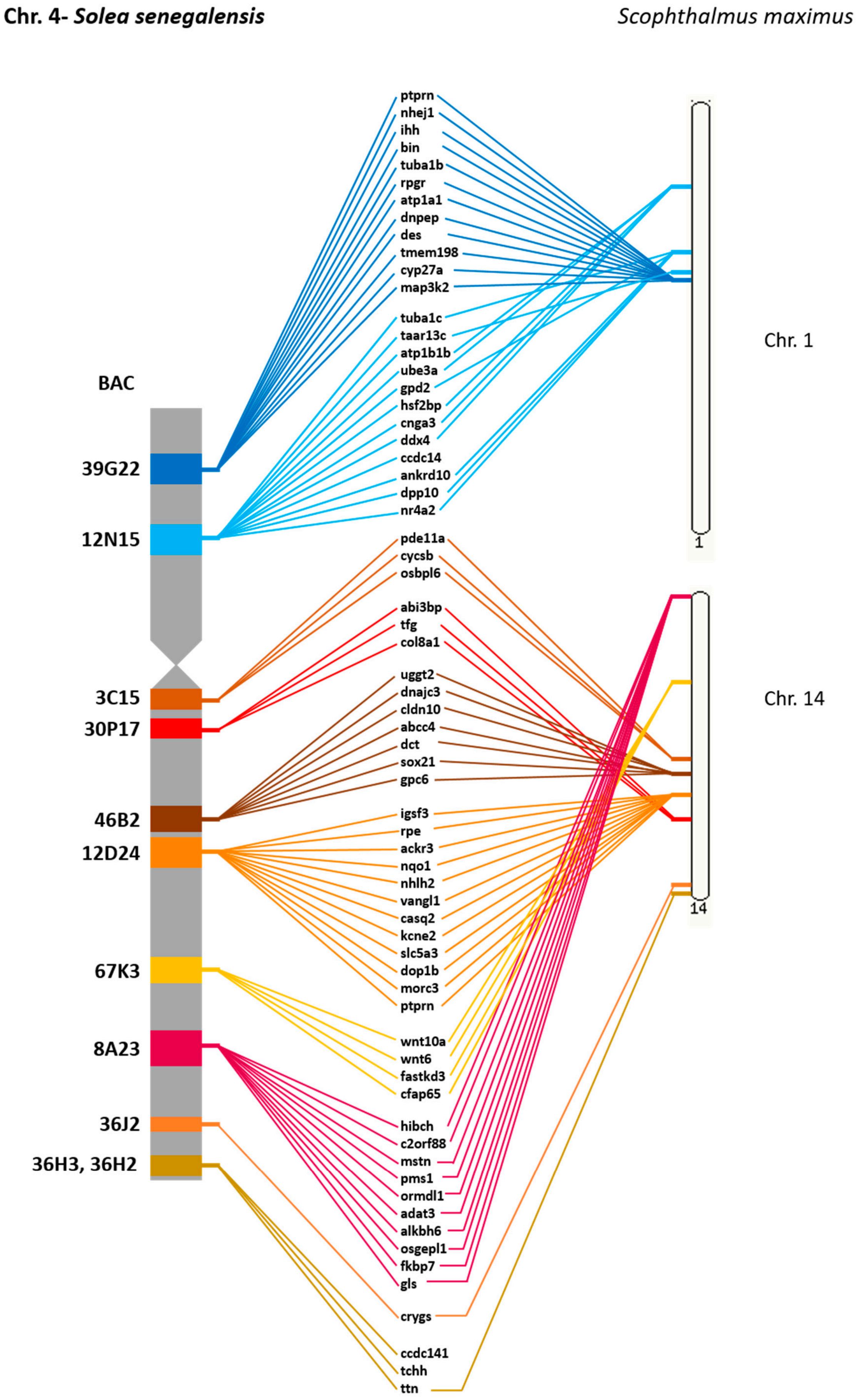

Chr. 4- Solea senegalensis
Scophthalmus maximus
BAC
39G22
12N15
3C15
30P17
46B2
12D24
67K3
8A23
36J2
36H3, 36H2
ptprn
nhej1
ihh
bin
tuba1b
rpgr
atp1a1
dnpep
des
tmem198
cyp27a
map3k2
tuba1c
taar13c
atp1b1b
ube3a
gpd2
hsf2bp
cnga3
ddx4
ccdc14
ankrd10
dpp10
nr4a2
pde11a
cycsb
osbpl6
abi3bp
tfg
col8a1
uggt2
dnajc3
cldn10
abcc4
dct
sox21
gpc6
igsf3
rpe
ackr3
nqo1
nhlh2
vangl1
casq2
kcne2
slc5a3
dop1b
morc3
ptprn
wnt10a
wnt6
fastkd3
cfap65
hibch
c2orf88
mstn
pms1
ormdl1
adat3
alkbh6
osgepl1
fkbp7
gls
crygs
ccdc141
tchh
ttn
Chr. 1
1
Chr. 14
14

Figure S9. Syntenic map of the submetacentric chromosome 4 of *Solea senegalensis* with respect to *Scophthalmus maximus*.

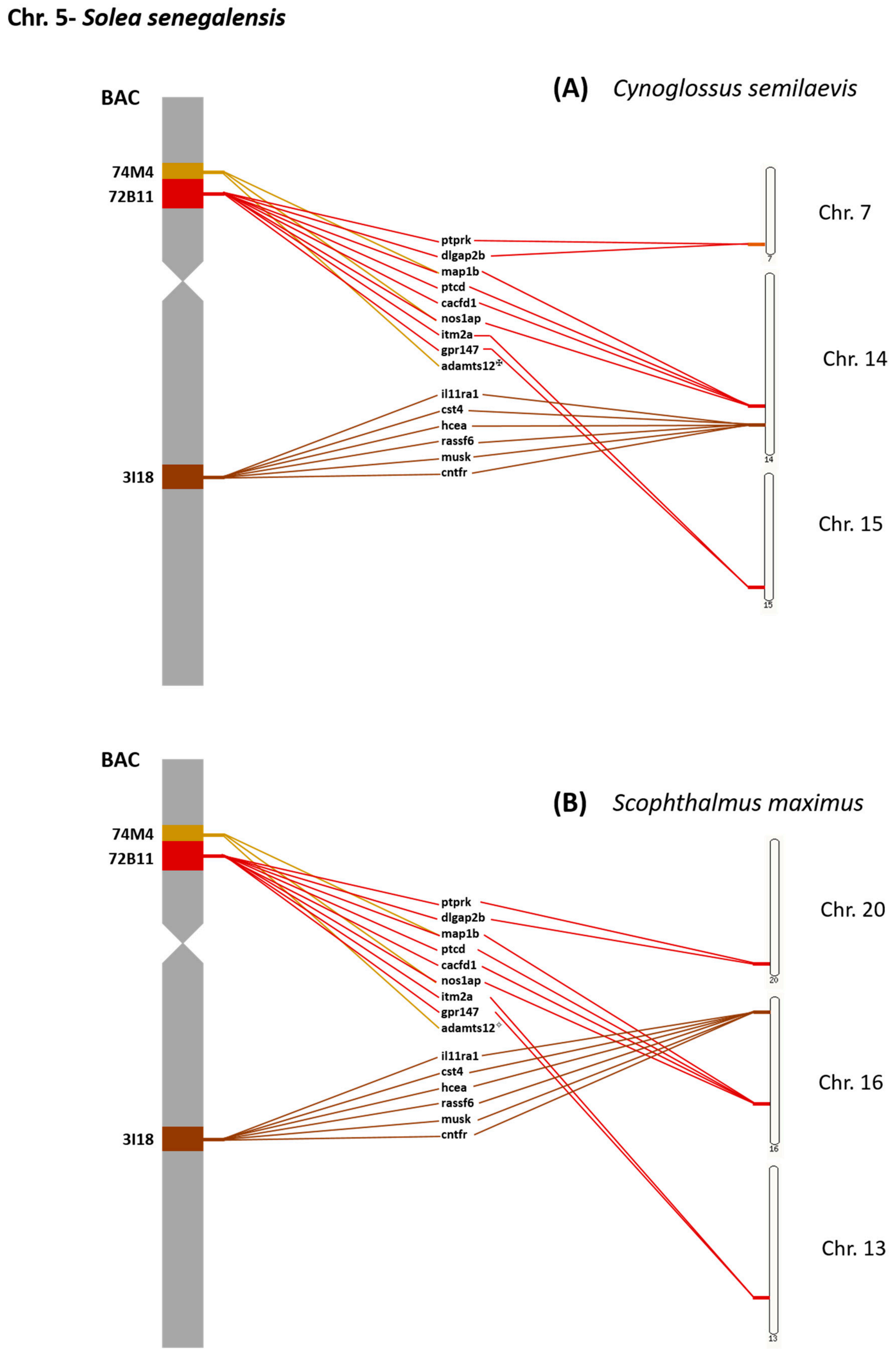

Figure S10. Syntenic map of the submetacentric chromosome 5 of *Solea senegalensis* with respect to (A) *Cynoglossus semilaevis* and (B) *Scophthalmus maximus*. Legend symbols: ✠Gene found in chromosome Z of *C. semilaevis*. ✧Gene found in chromosome 9 of *S. maximus*.

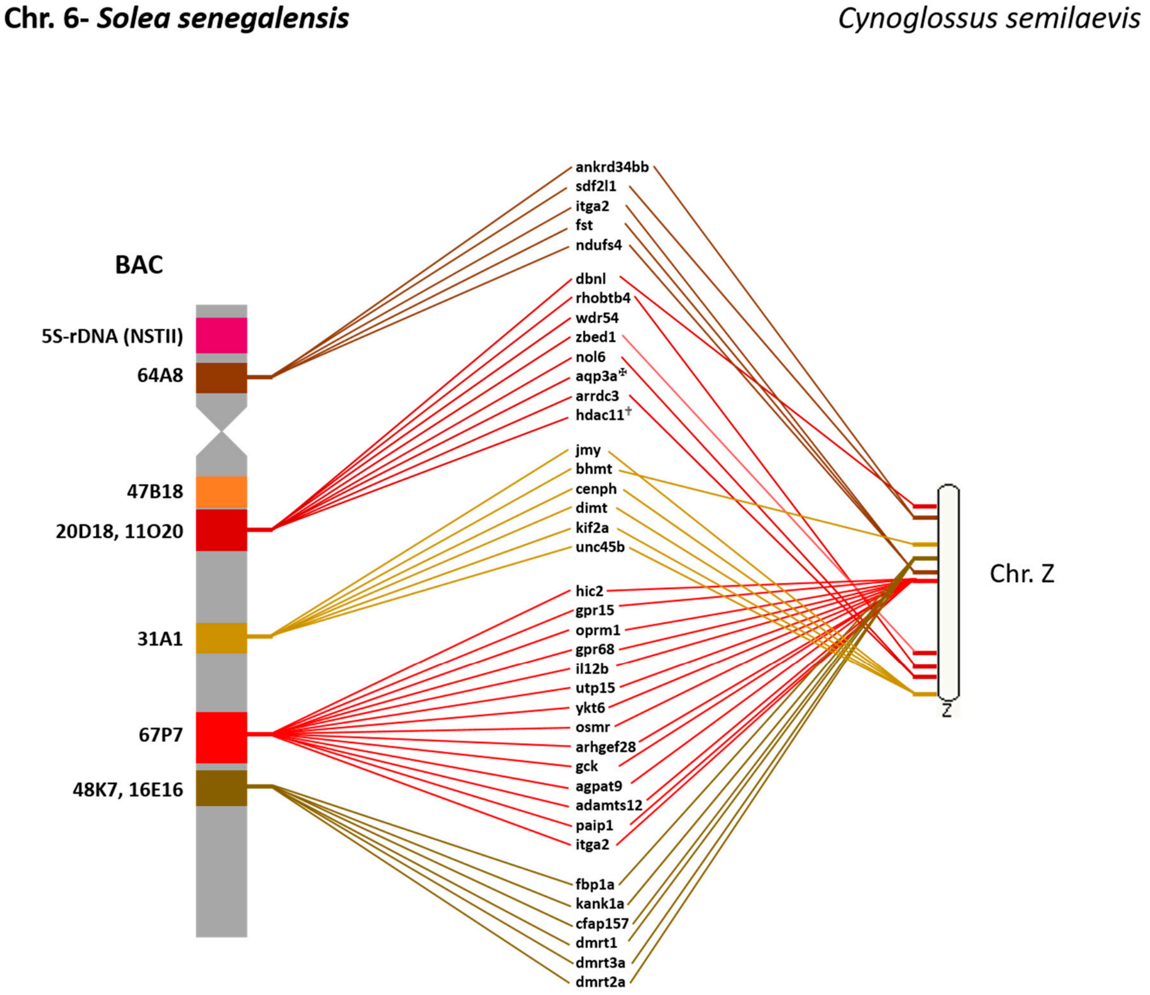

Figure S11. Syntenic map of the subtelocentric chromosome 6 of *Solea senegalensis* with respect to *Cynoglossus semilaevis*. Legend symbols: ✠Gene found in chromosome 14 of *C. semilaevis*. †Gene found in chromosome 11 of *C. semilaevis*.

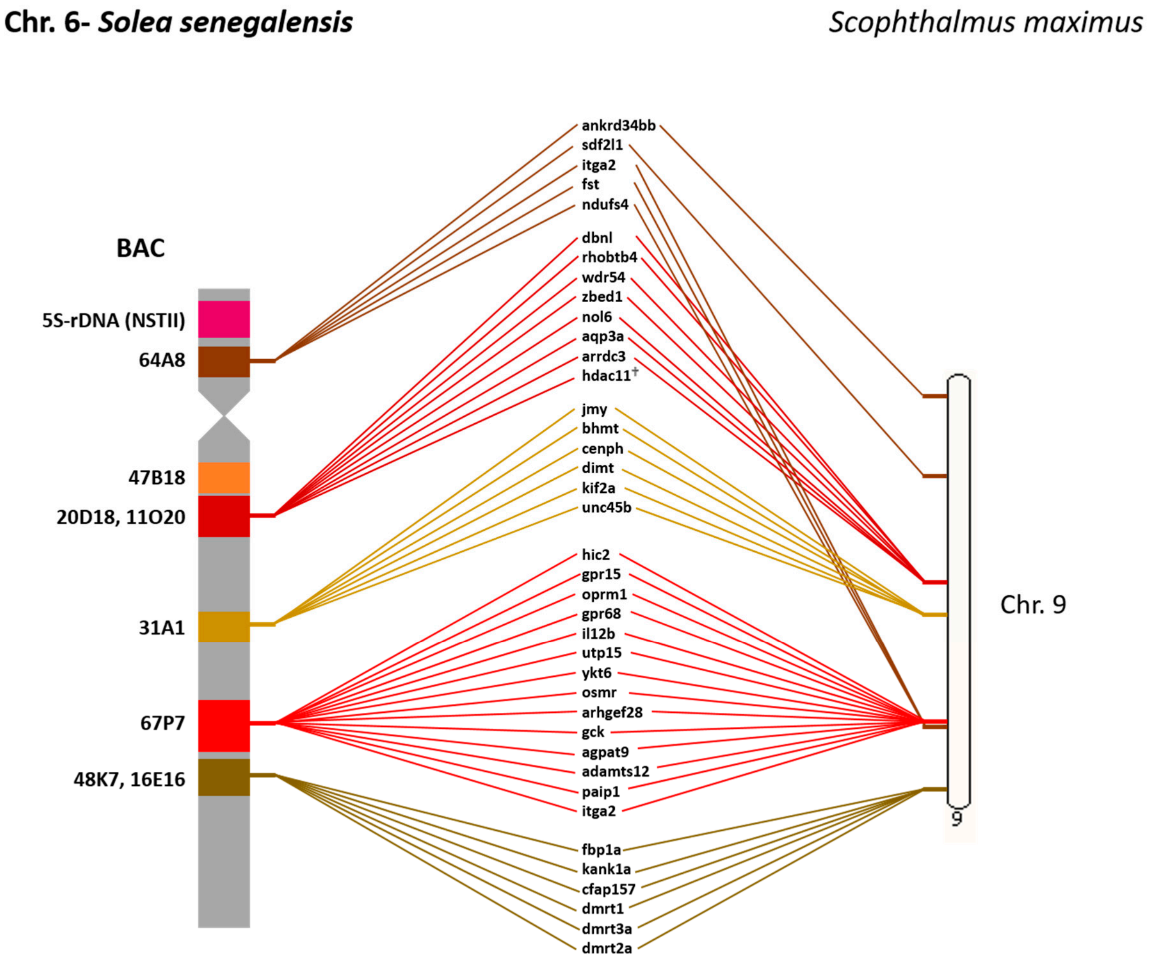

Figure S12. Syntenic map of the subtelocentric chromosome 6 of *Solea senegalensis* with respect to *Scophthalmus maximus*. Legend symbols: †Gene found in chromosome 6 of *S. maximus*.

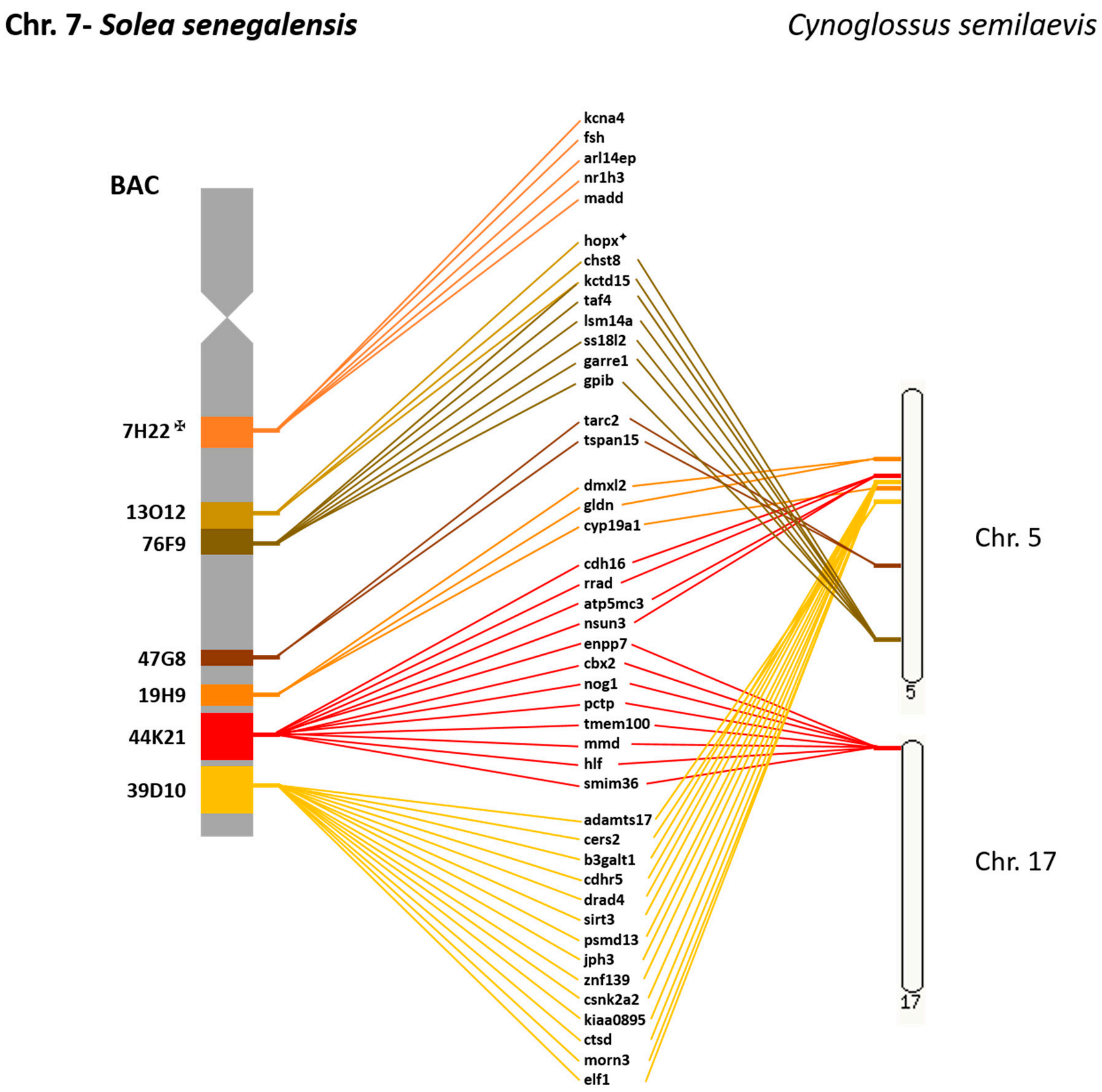

Figure S13. Syntenic map of the subtelocentric chromosome 7 of *Solea senegalensis* with respect to *Cynoglossus semilaevis*. Legend symbols: ✠BAC with sequences found in sacffold of *C. semilaevis*. ✦Gene found in chromosome 15 of *C. semilaevis*.

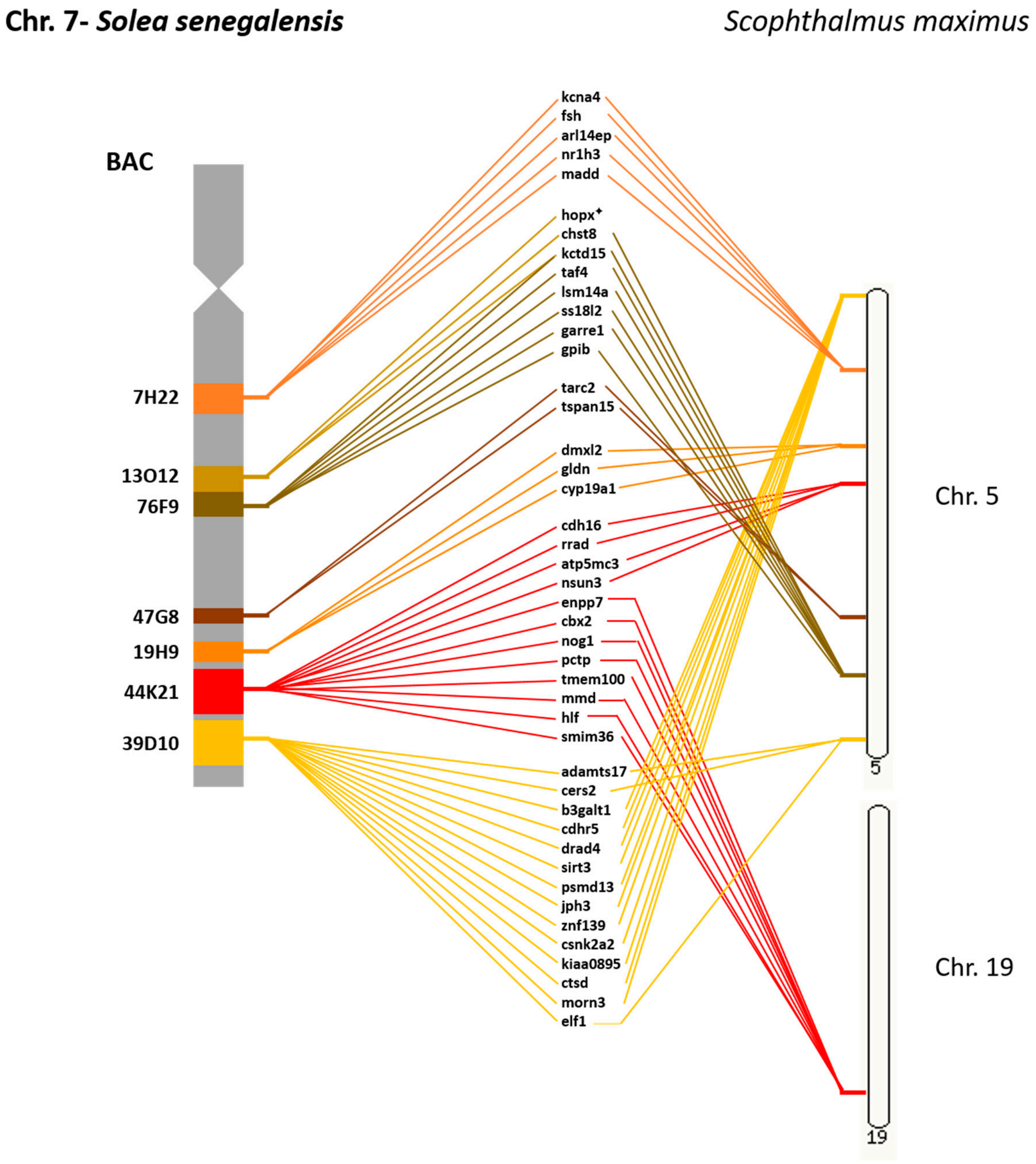

Figure S14. Syntenic map of the subtelocentric chromosome 7 of *Solea senegalensis* with respect to *Scophthalmus maximus*. Legend symbols: ✦Gene found in chromosome 13 of *S. maximus*.

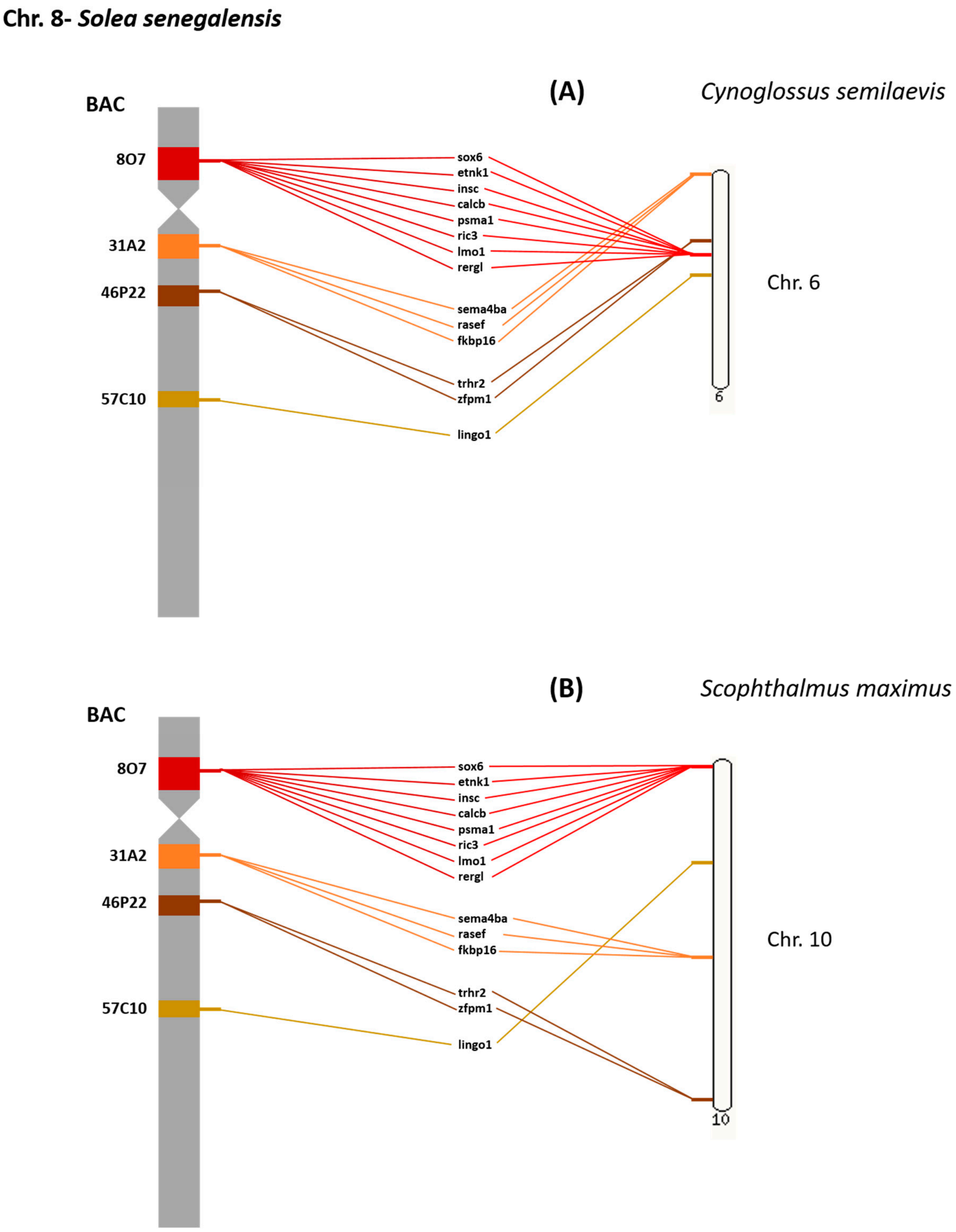

Figure S15. Syntenic map of the subtelocentric chromosome 8 of *Solea senegalensis* with respect to (A) *Cynoglossus semilaevis* and (B) *Scophthalmus maximus*.

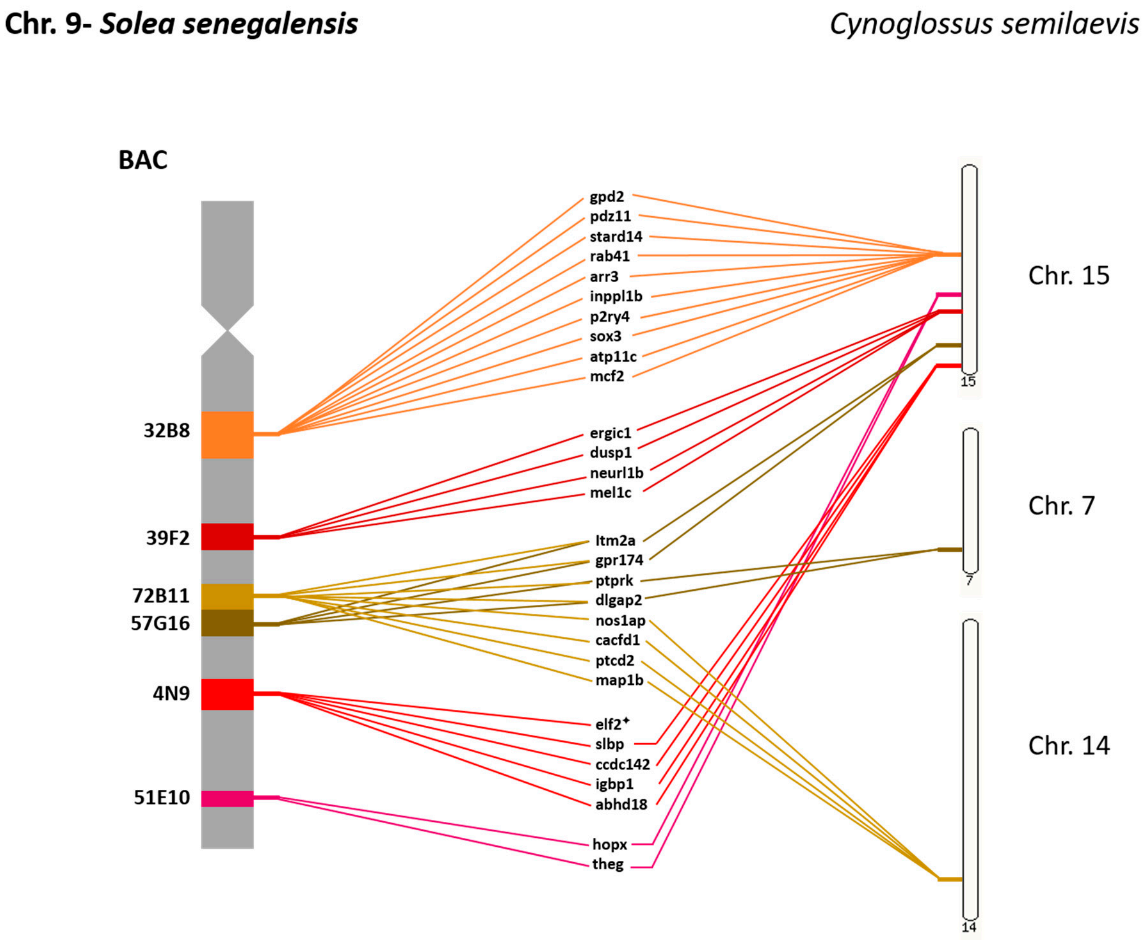

Figure S16. Syntenic map of the subtelocentric chromosome 9 of *Solea senegalensis* with respect to *Cynoglossus semilaevis*. Legend symbols: ✦Gene found in chromosome 9 of *C. semilaevis*.

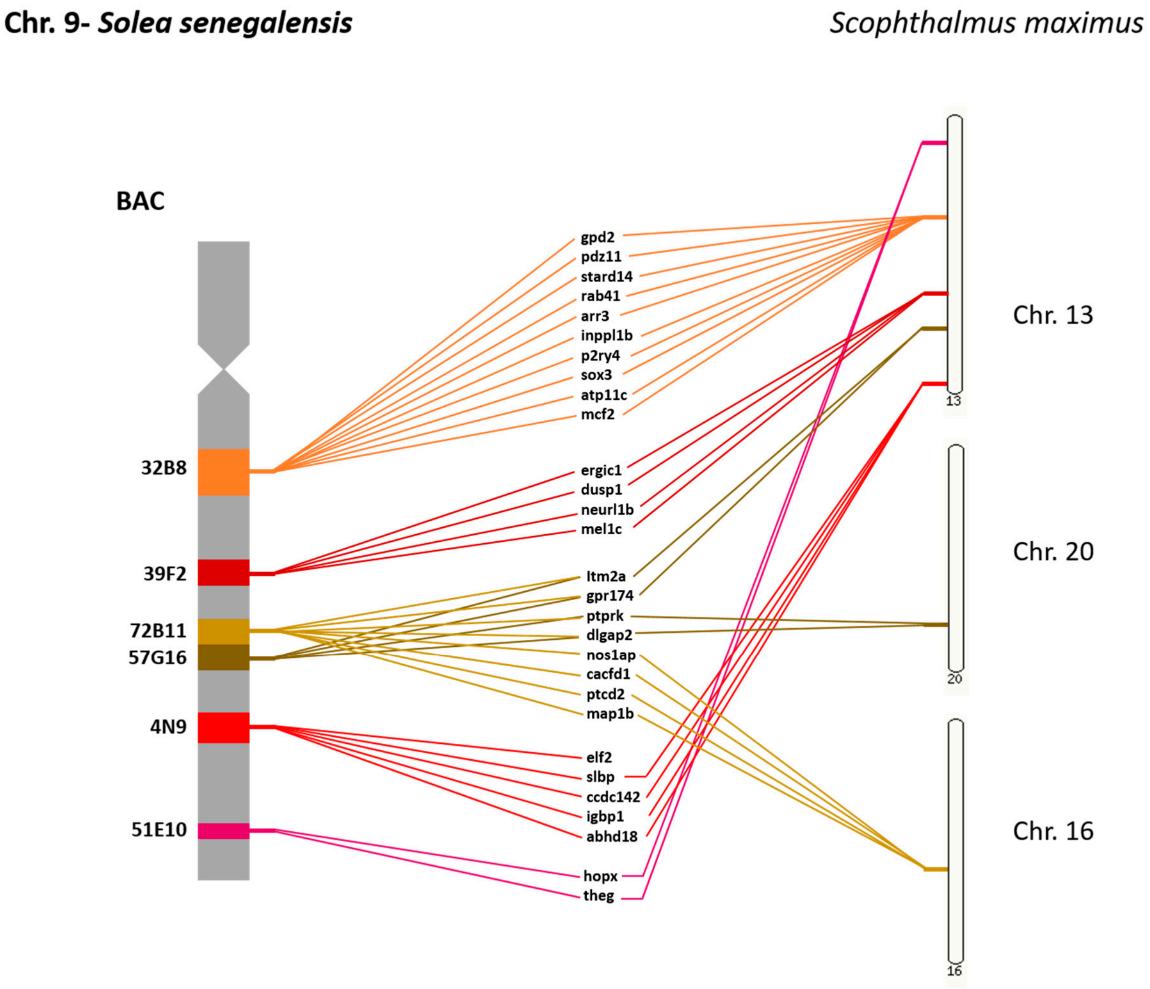

Figure S17. Syntenic map of the subtelocentric chromosome 9 of *Solea senegalensis* with respect to *Scophthalmus maximus*.

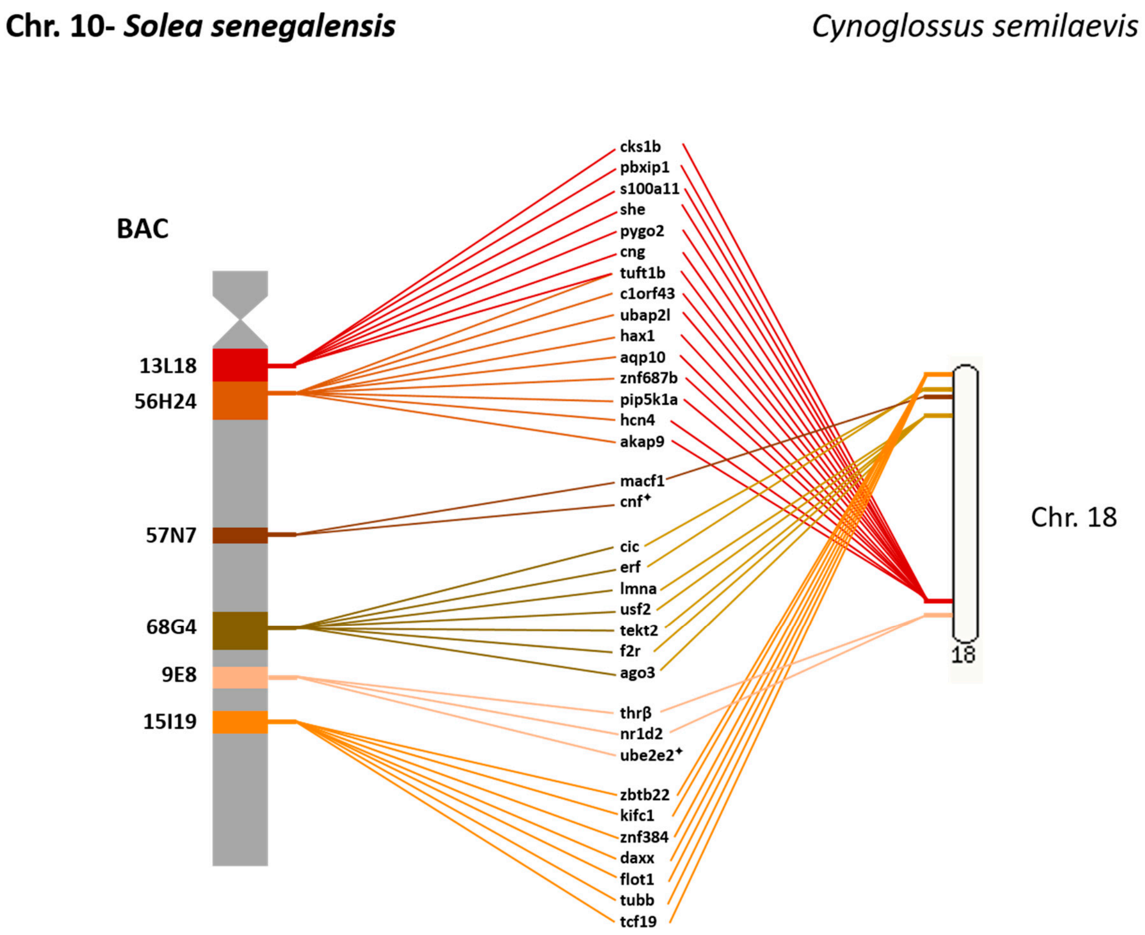

Figure S18. Syntenic map of the acrocentric chromosome 10 of *Solea senegalensis* with respect to *Cynoglossus semilaevis*. Legend symbols: ✦Genes found in chromosome 13 of *C. semilaevis*.

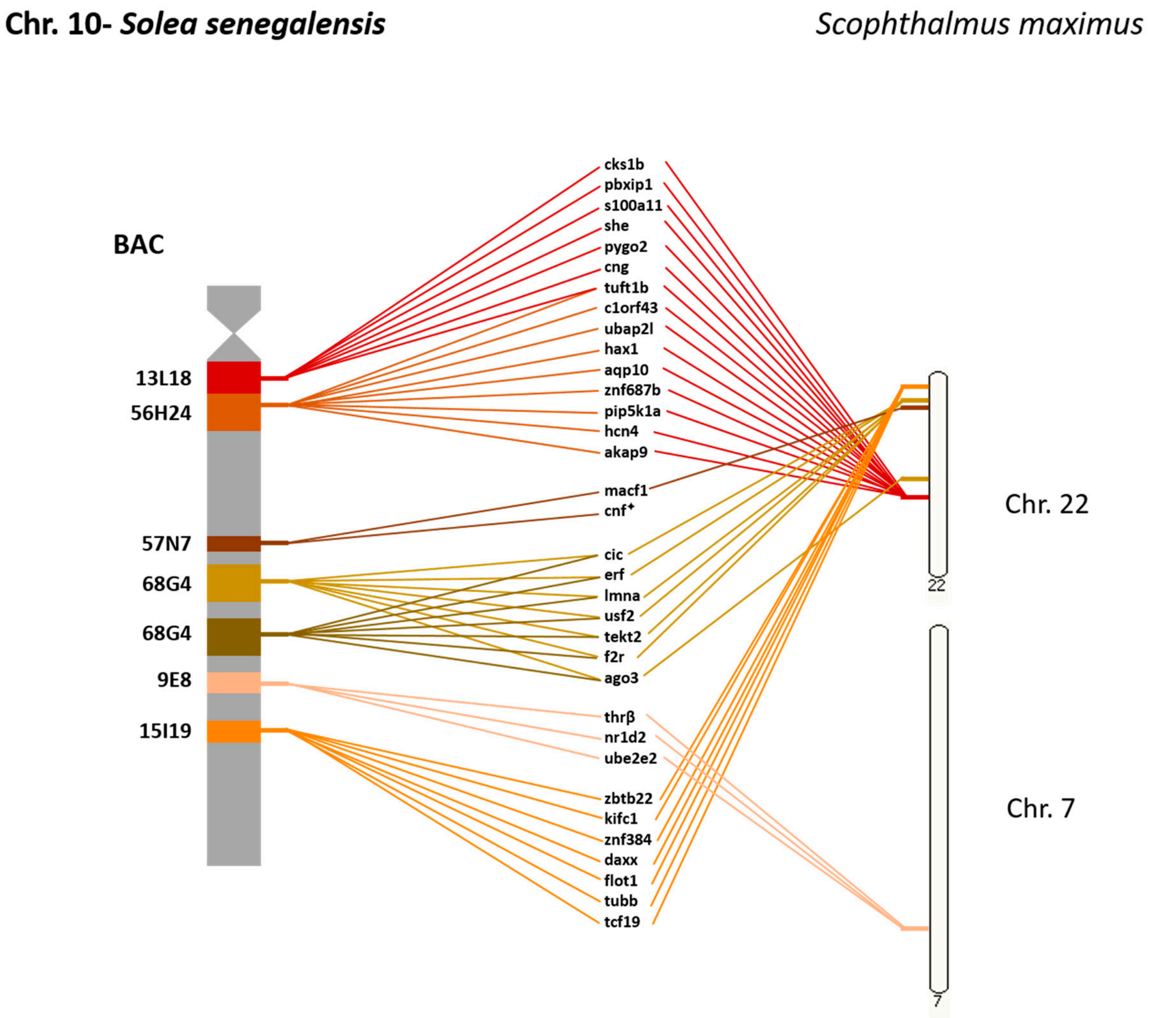

Figure S19. Syntenic map of the acrocentric chromosome 10 of *Solea senegalensis* with respect to *Scophthalmus maximus*. Legend symbols: ✦Gene found in chromosome 1 of *S. maximus.*

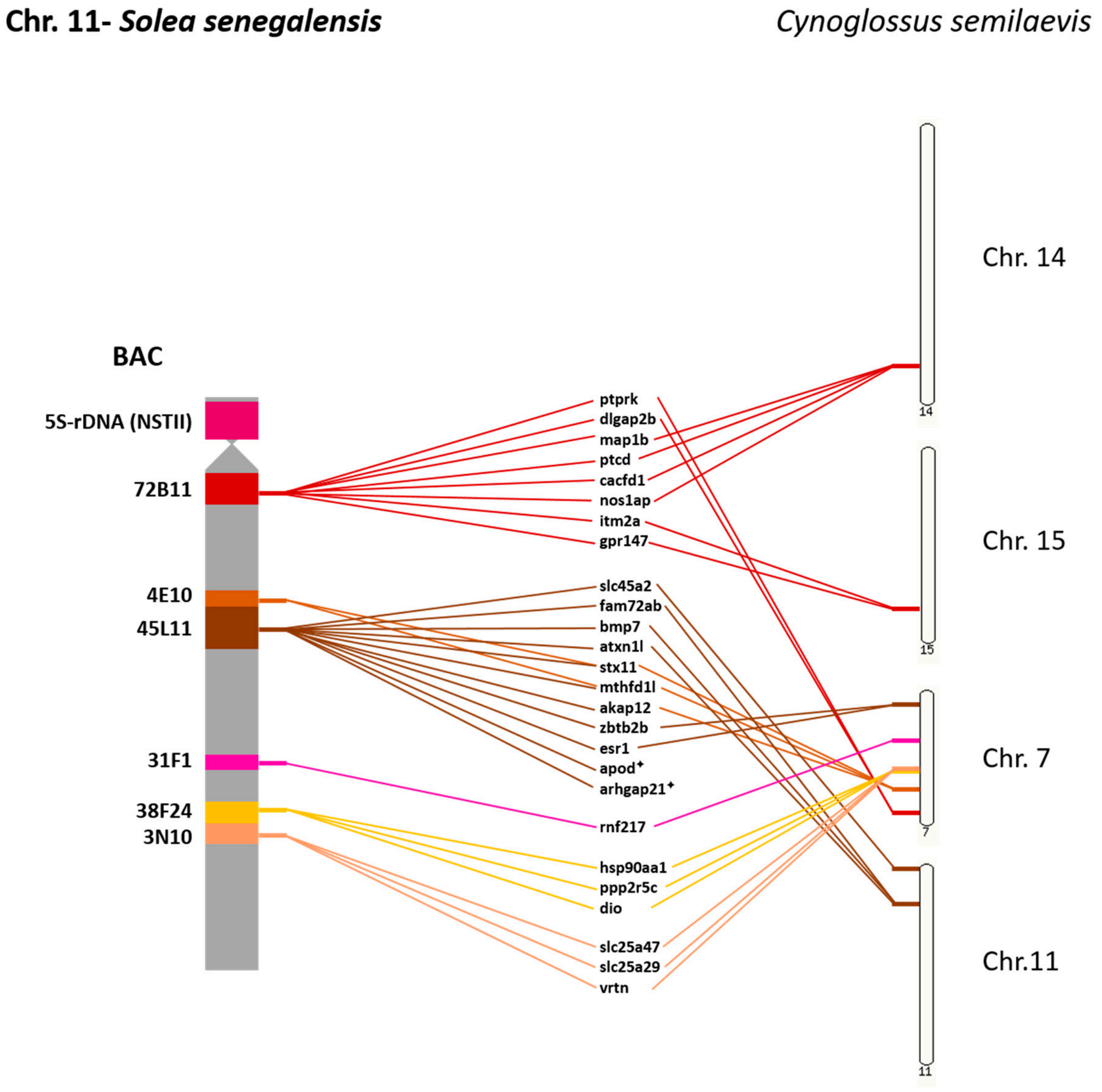

Figure S20. Syntenic map of the acrocentric chromosome 11 of *Solea senegalensis* with respect to *Cynoglossus semilaevis*. Legend symbols: ✦Genes found in chromosome 20 of *C. semilaevis*.

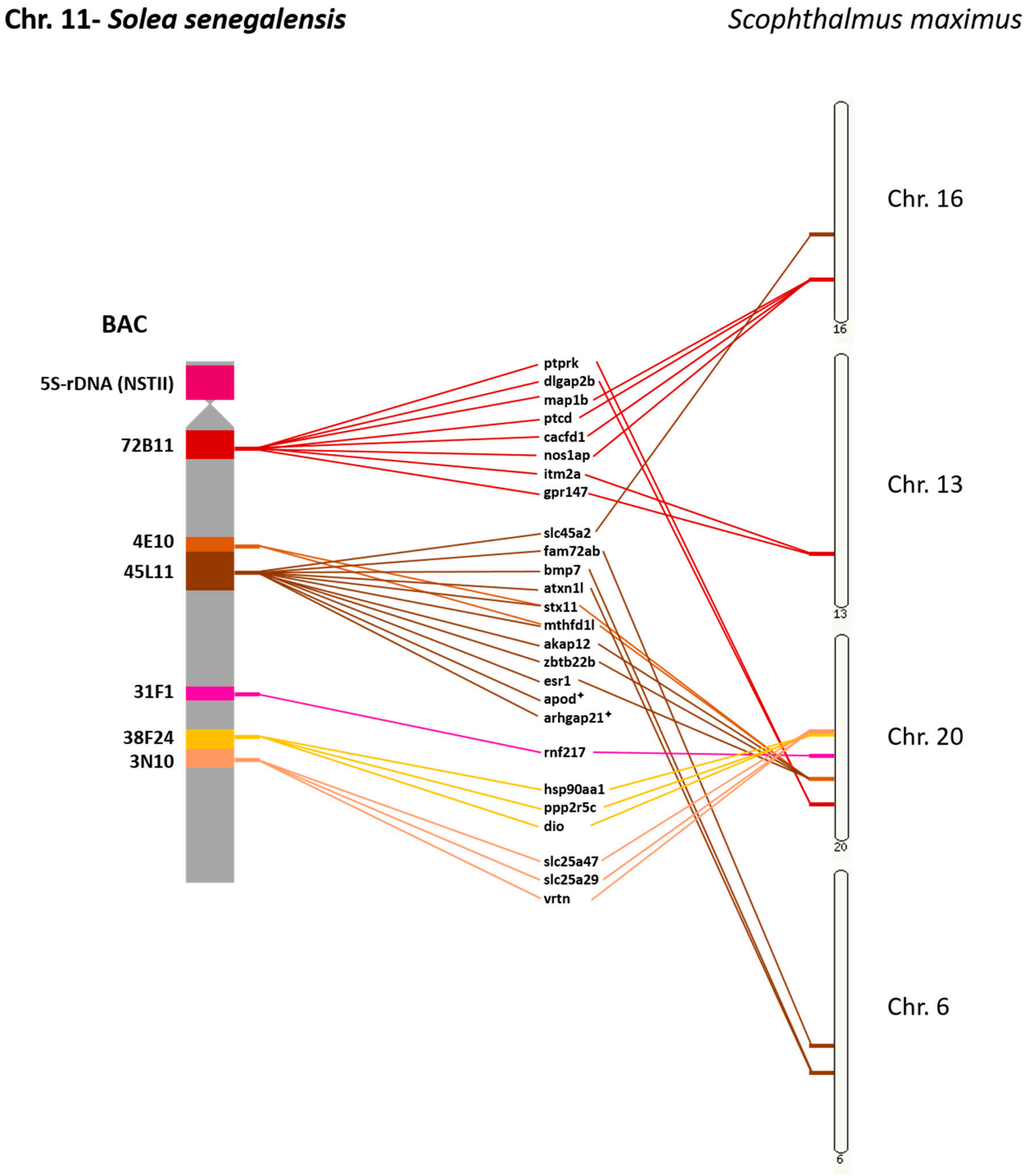

Figure S21. Syntenic map of the acrocentric chromosome 11 of *Solea senegalensis* with respect to *Scophthalmus maximus*. Legend symbols: ✦Genes found in chromosome 21 of *S. maximus*.

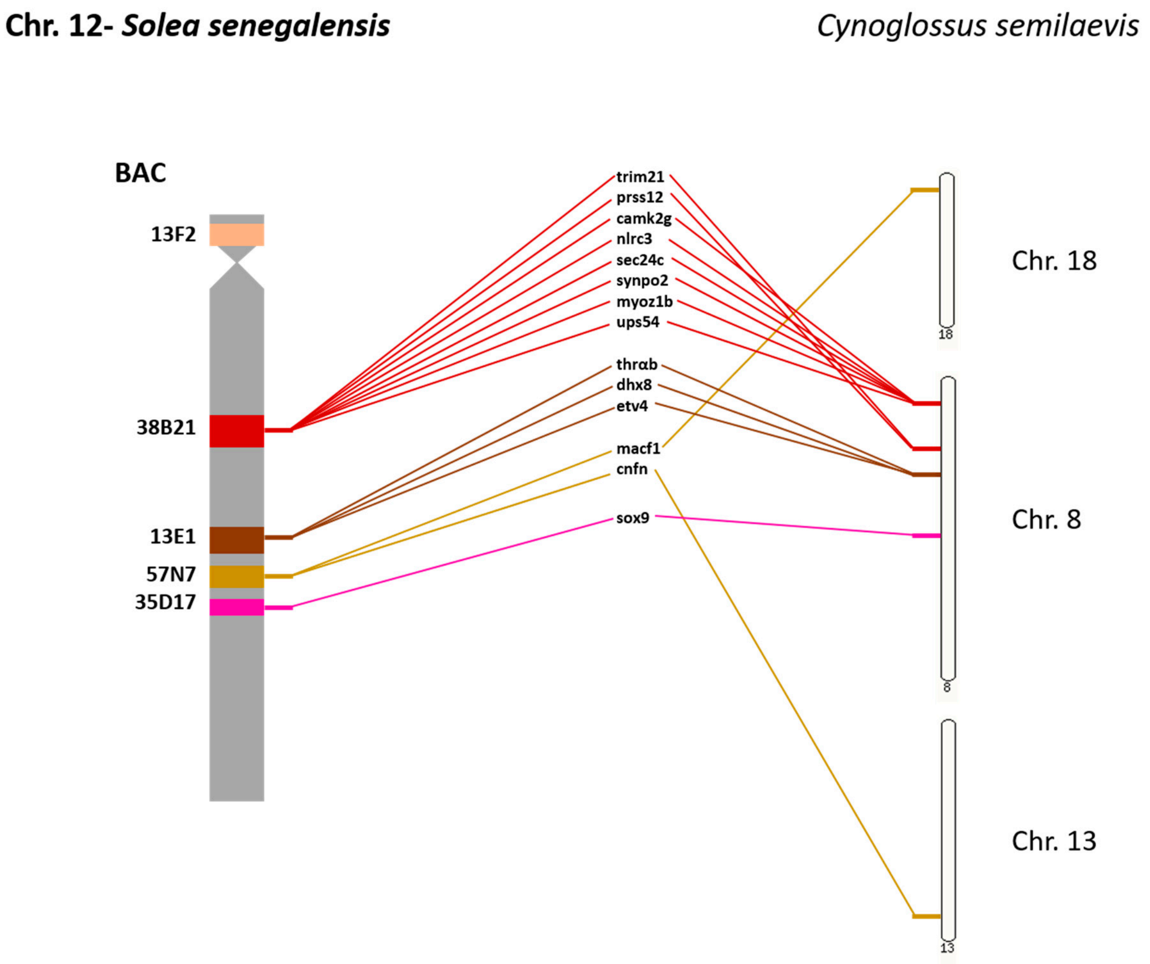

Figure S22. Syntenic map of the acrocentric chromosome 12 of *Solea senegalensis* with respect to *Cynoglossus semilaevis*.

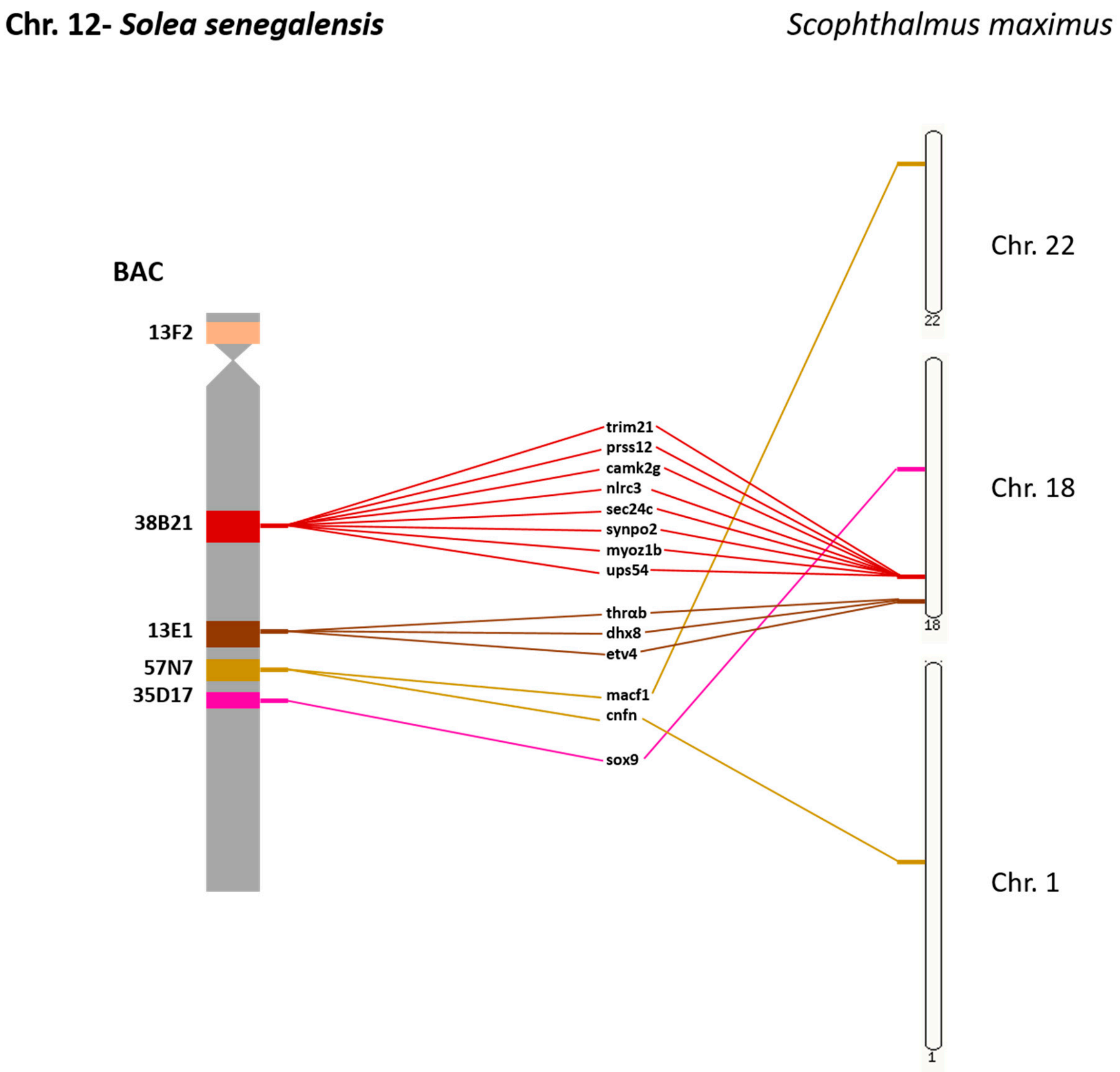

Figure S23. Syntenic map of the acrocentric chromosome 12 of *Solea senegalensis* with respect to *Scophthalmus maximus*.

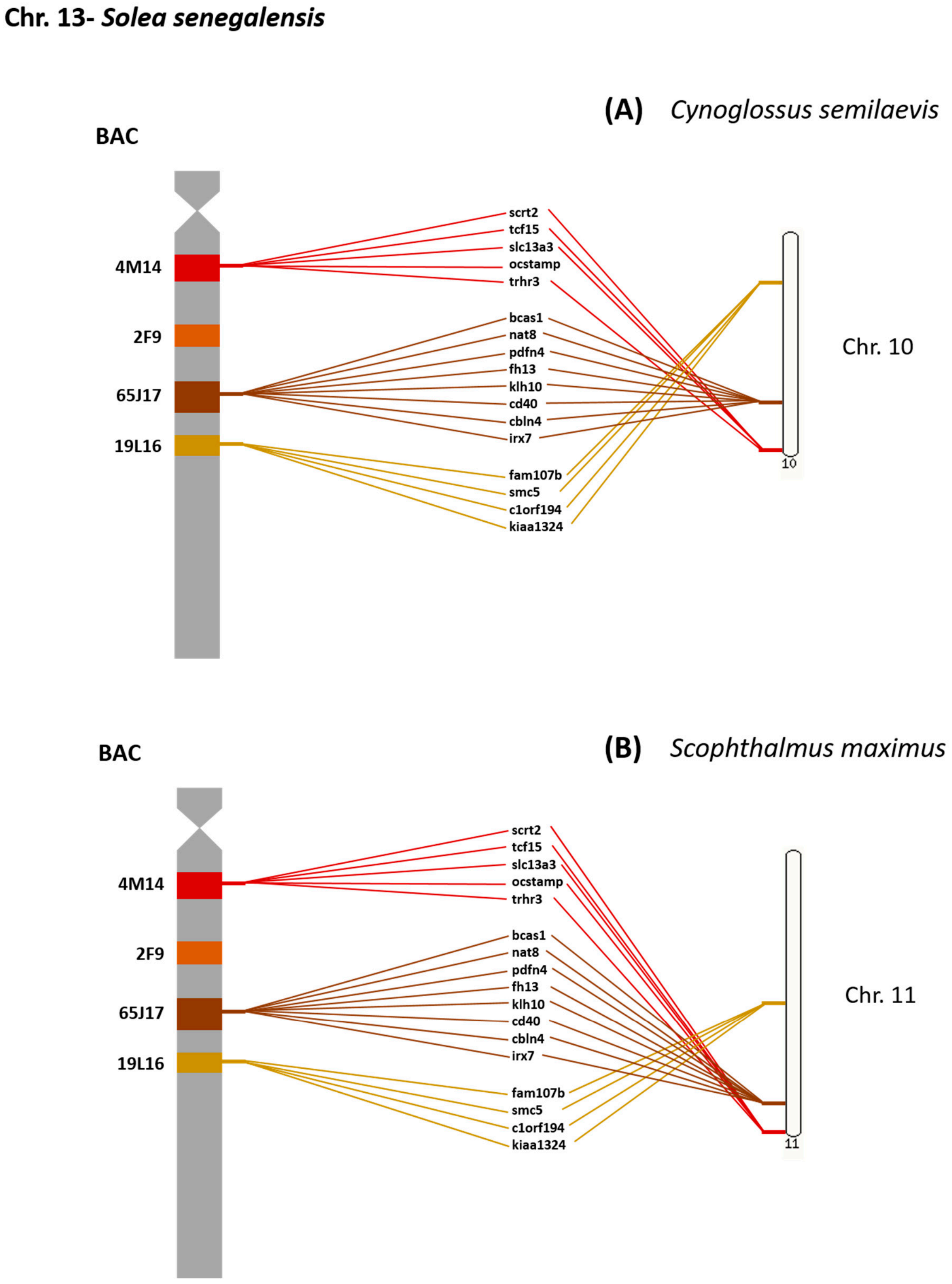

Figure S24. Syntenic map of the acrocentric chromosome 13 of *Solea senegalensis* with respect to (A) *Cynoglossus semilaevis* and (B) *Scophthalmus maximus*.

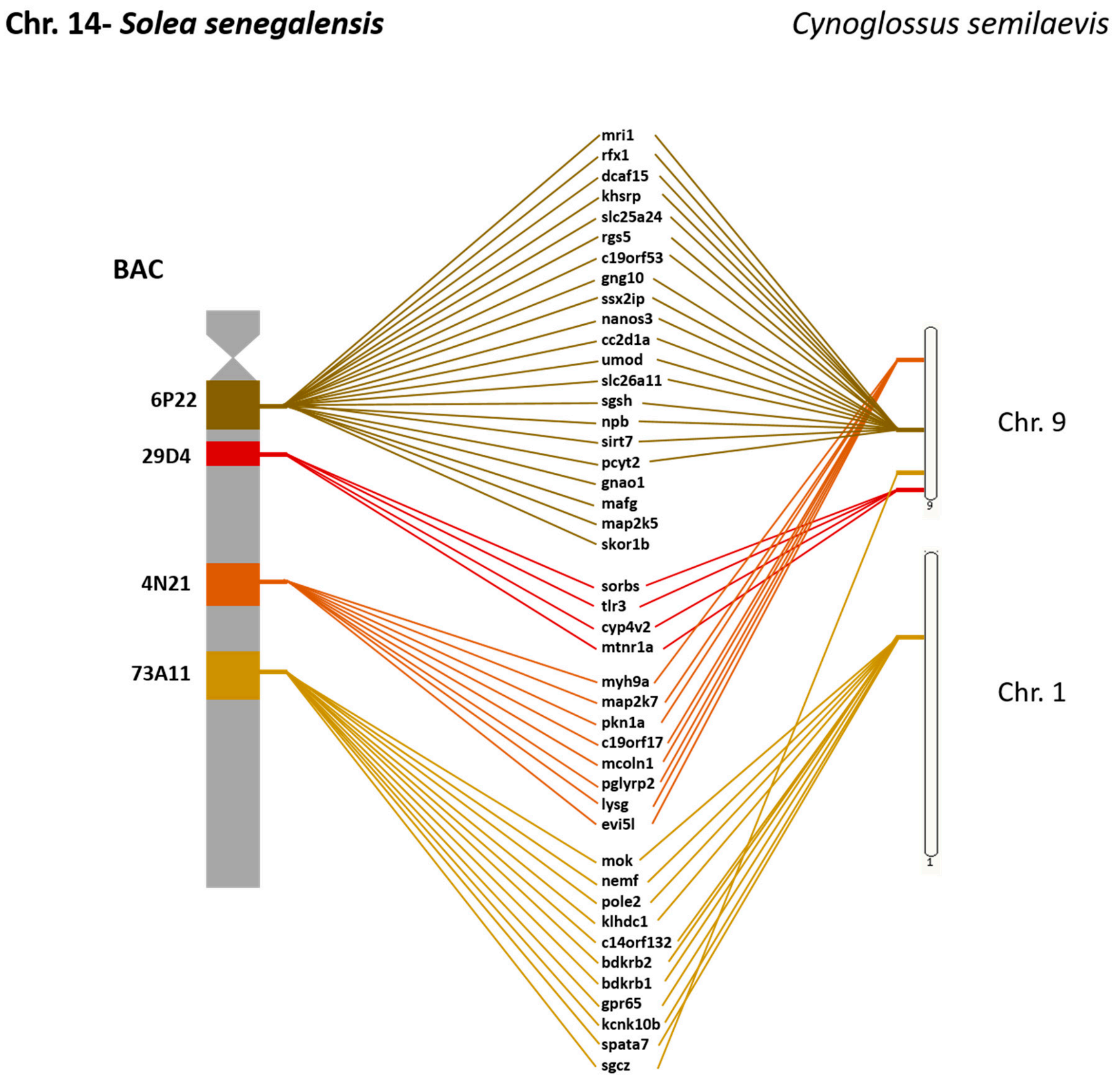

Figure S25. Syntenic map of the acrocentric chromosome 14 of *Solea senegalensis* with respect to *Cynoglossus semilaevis*.

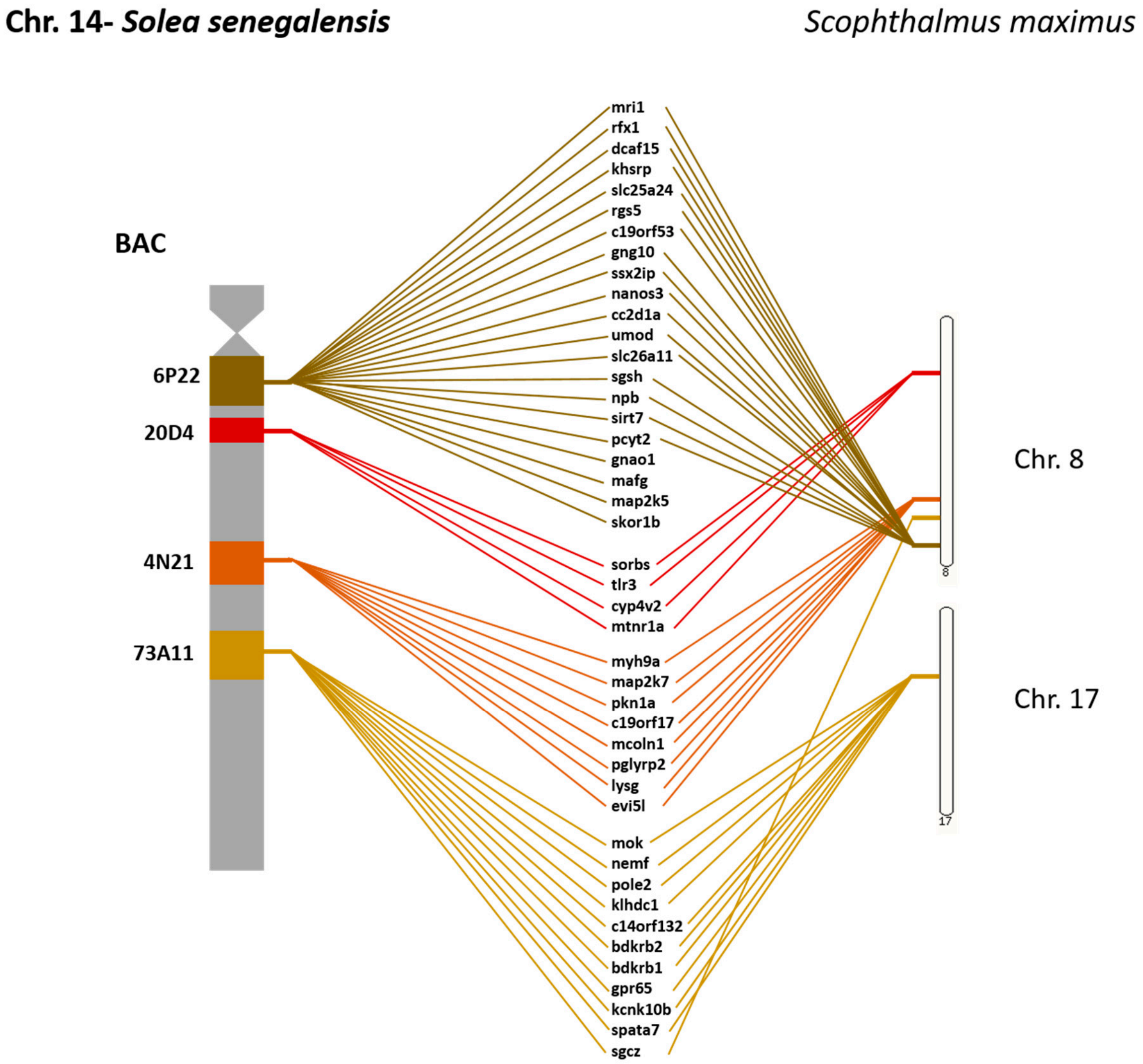

Figure S26. Syntenic map of the acrocentric chromosome 14 of *Solea senegalensis* with respect to *Scophthalmus maximus*.

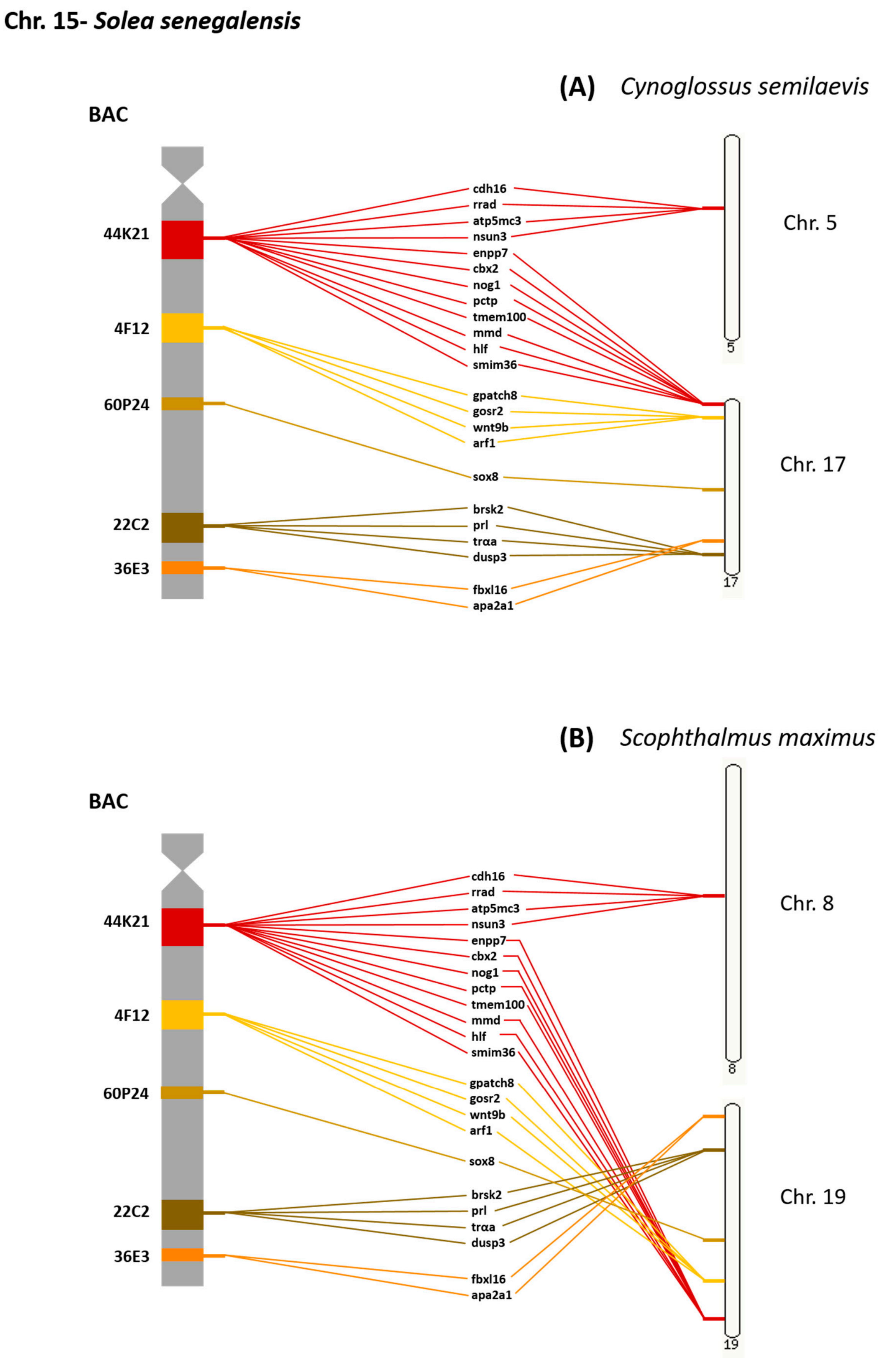

Figure S27. Syntenic map of the acrocentric chromosome 15 of *Solea senegalensis* with respect to (A) *Cynoglossus semilaevis* and (B) *Scophthalmus maximus*.

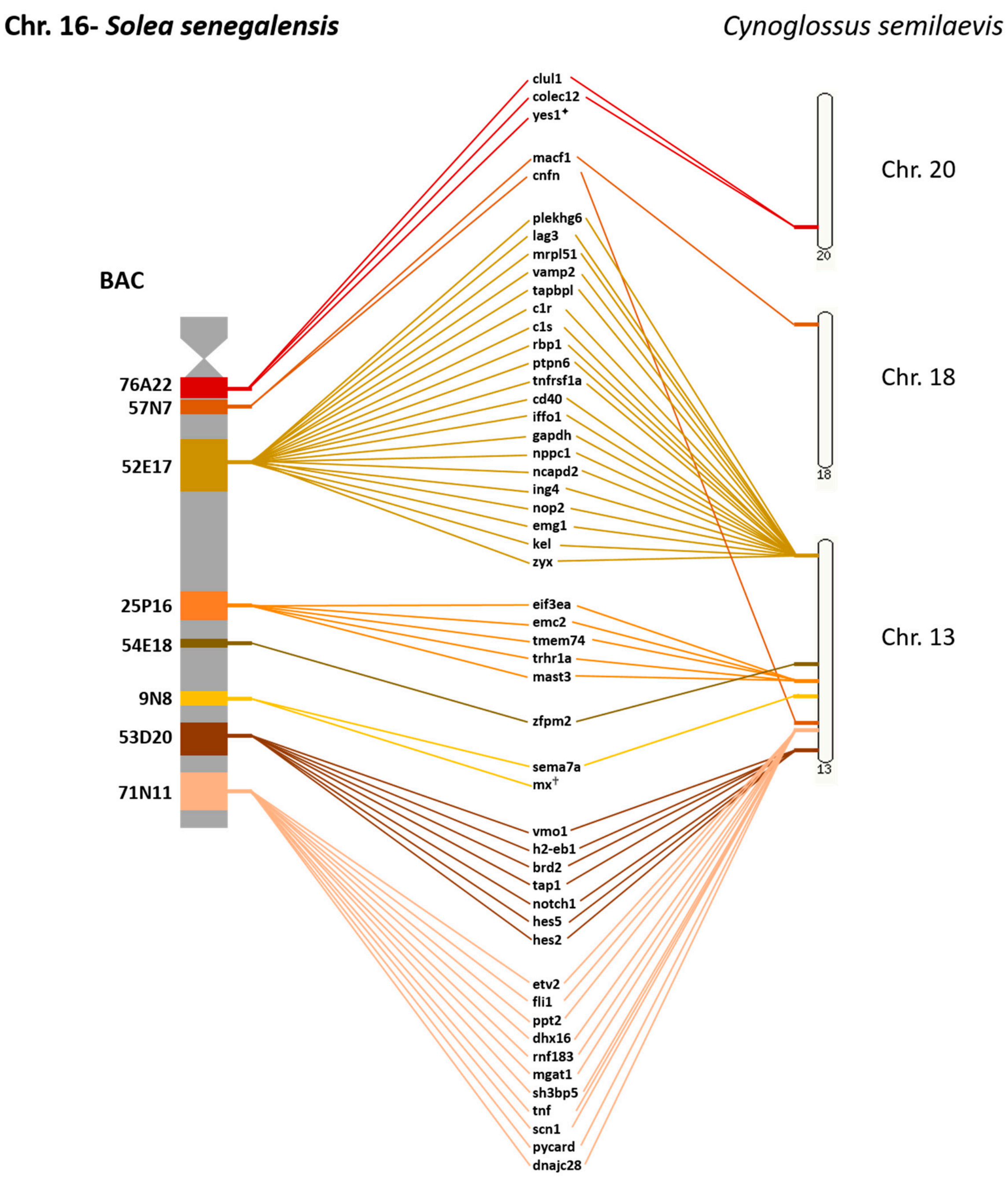

Figure S28. Syntenic map of the acrocentric chromosome 16 of *Solea senegalensis* with respect to *Cynoglossus semilaevis*. Legend symbols: ✦Gene found in chromosome 3 of *C. semilaevis*. †Gene found in chromosome 11 of *C. semilaevis*.

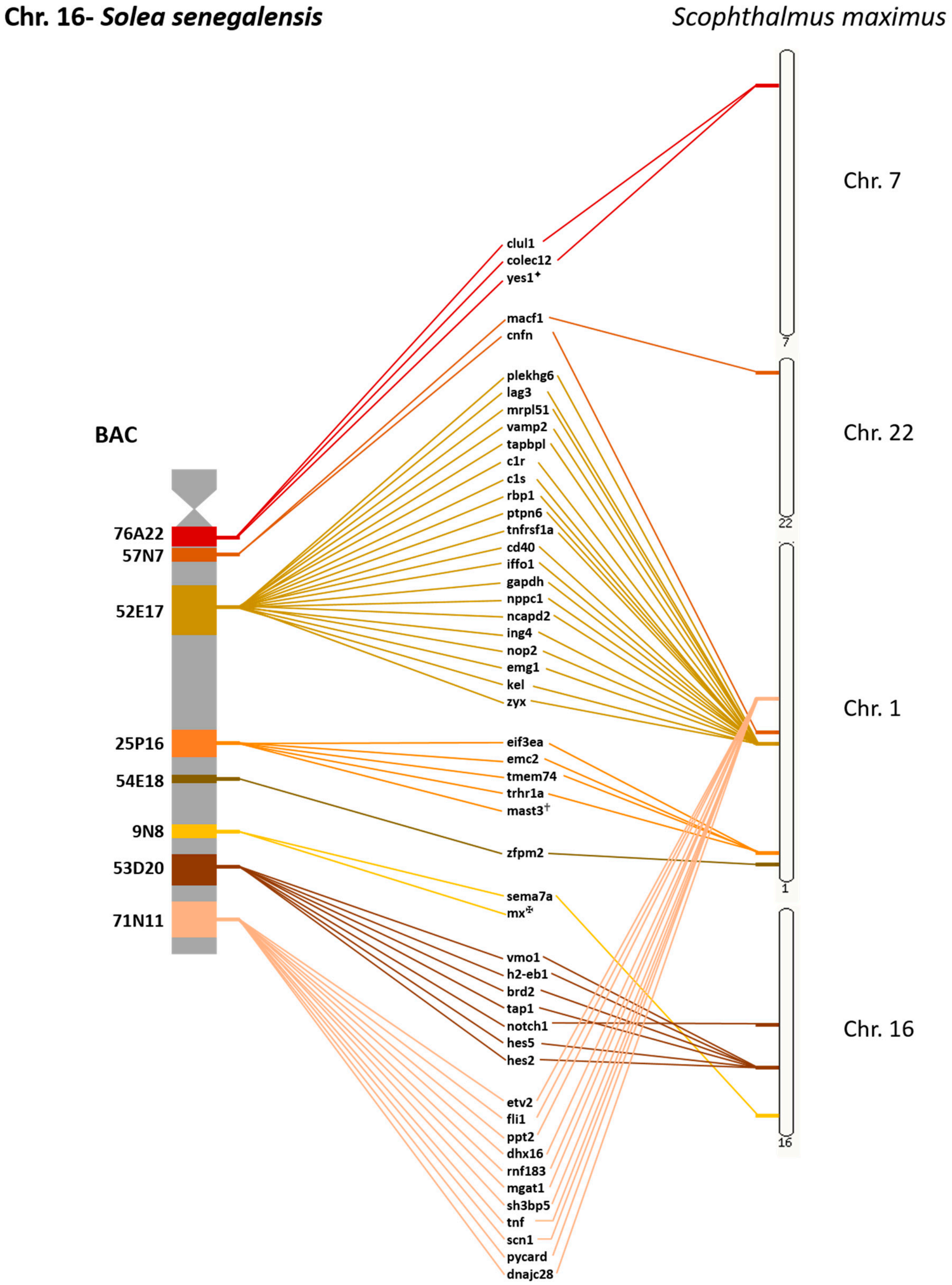

Figure S29. Syntenic map of the acrocentric chromosome 16 of *Solea senegalensis* with respect to *Scophthalmus maximus*. Legend symbols: ✦Genes found in chromosome 18 of *S. maximus*. †Gene found in chromosome 12 of *S. maximus*. ✠Gene found in chromosome 6 of *S. maximus*.

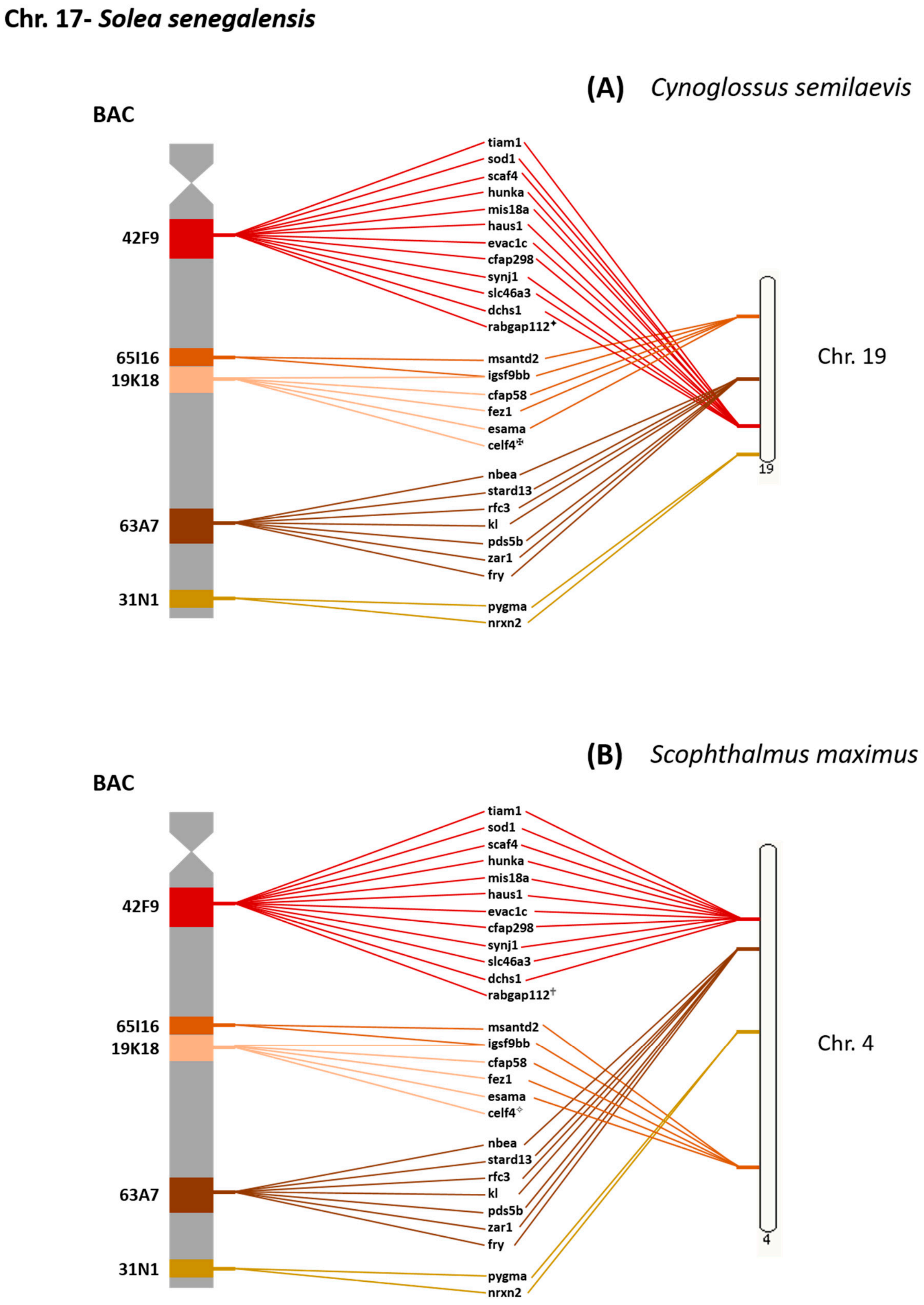

Figure S30. Syntenic map of the acrocentric chromosome 17 of *Solea senegalensis* with respect to (A) *Cynoglossus semilaevis* and (B) *Scophthalmus maximus*. Legend symbols: ✦Genes found in chromosome 1 of *C. semilaevis*. ✠Gene found in chromosome 5 of *C. semilaevis*. †Gene found in chromosome 17 of *S. maximus*. ✧Gene found in chromosome 5 of *S. maximus*.

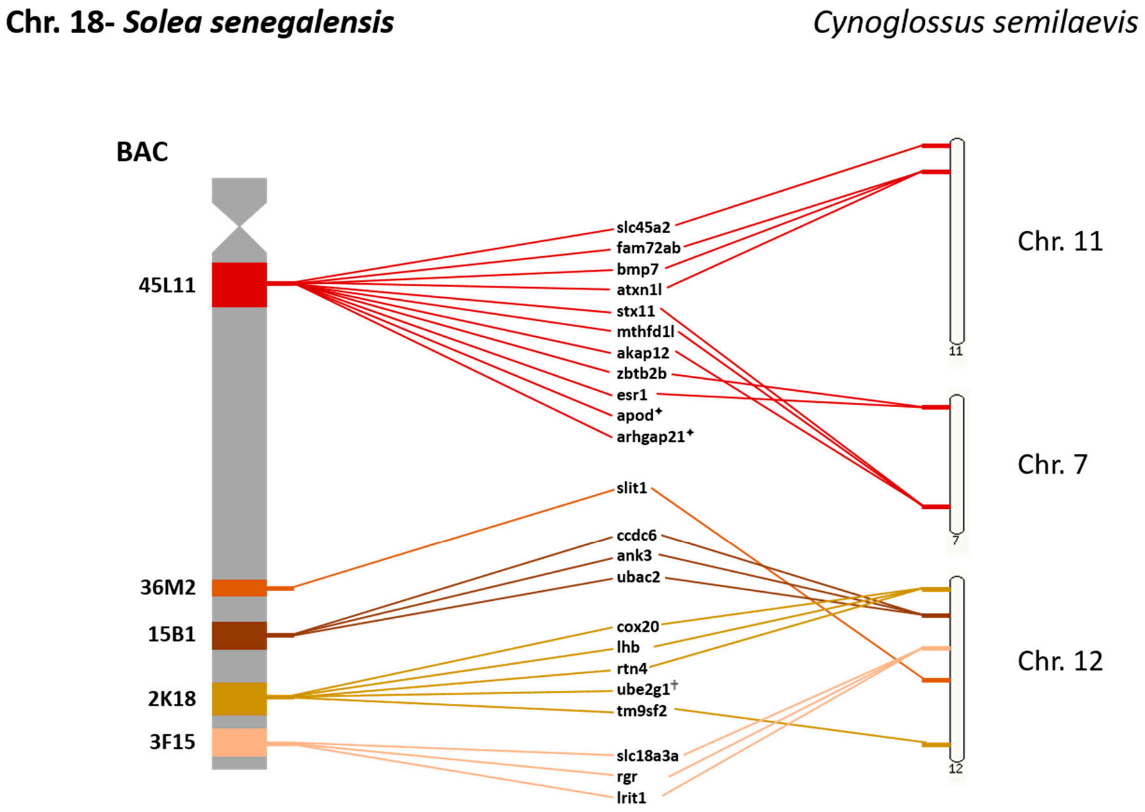

Figure S31. Syntenic map of the acrocentric chromosome 18 of *Solea senegalensis* with respect to *Cynoglossus semilaevis*. Legend symbols: ✦Genes found in chromosome 20 of *C. semilaevis*. †Gene found in chromosome 14 of *C. semilaevis*.

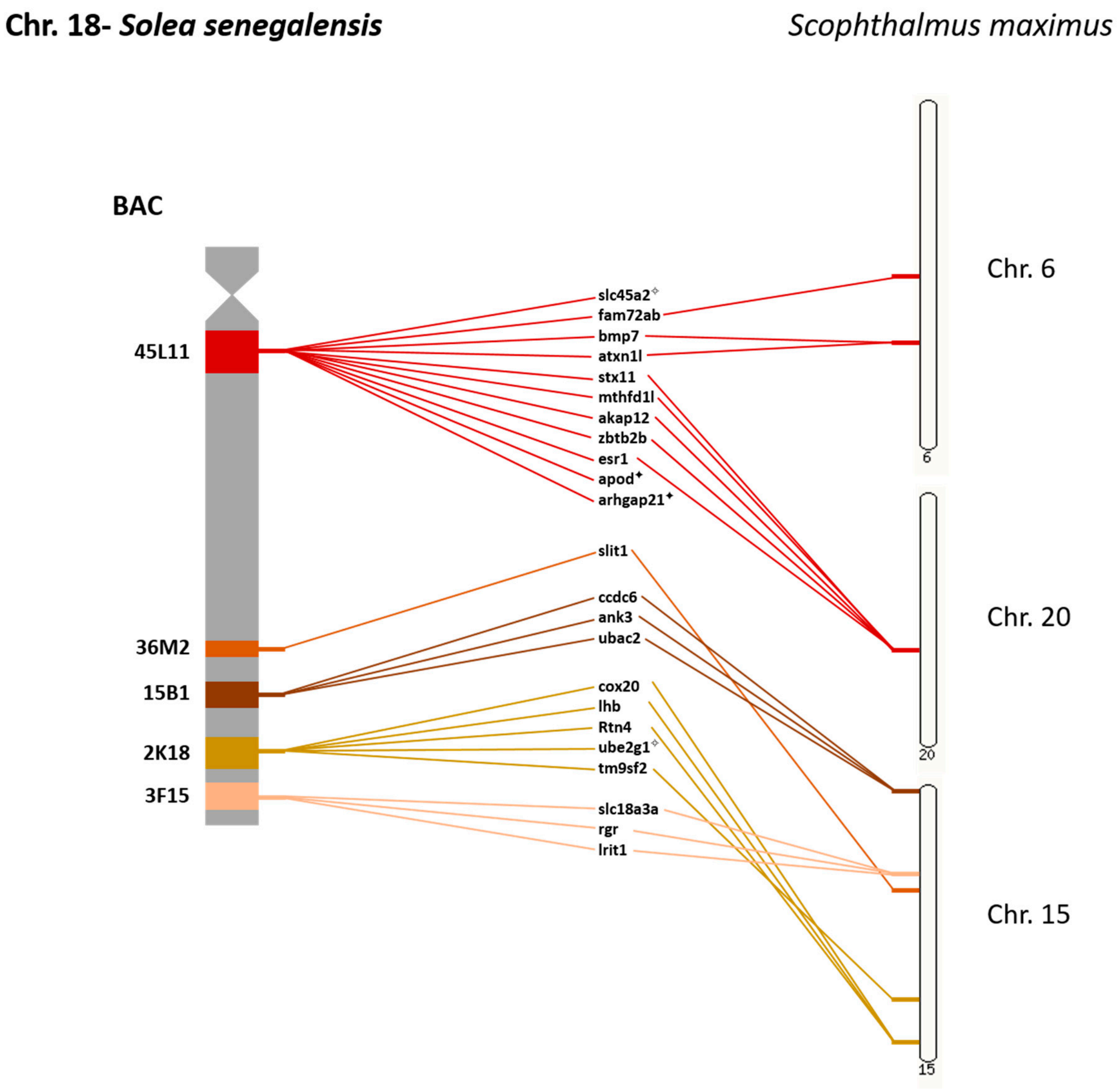

Figure S32. Syntenic map of the acrocentric chromosome 18 of *Solea senegalensis* with respect to *Scophthalmus maximus*. Legend symbols: ✧Genes found in chromosome 16 of *S. maximus*. ✦Genes found in chromosome 21 of *S. maximus*.

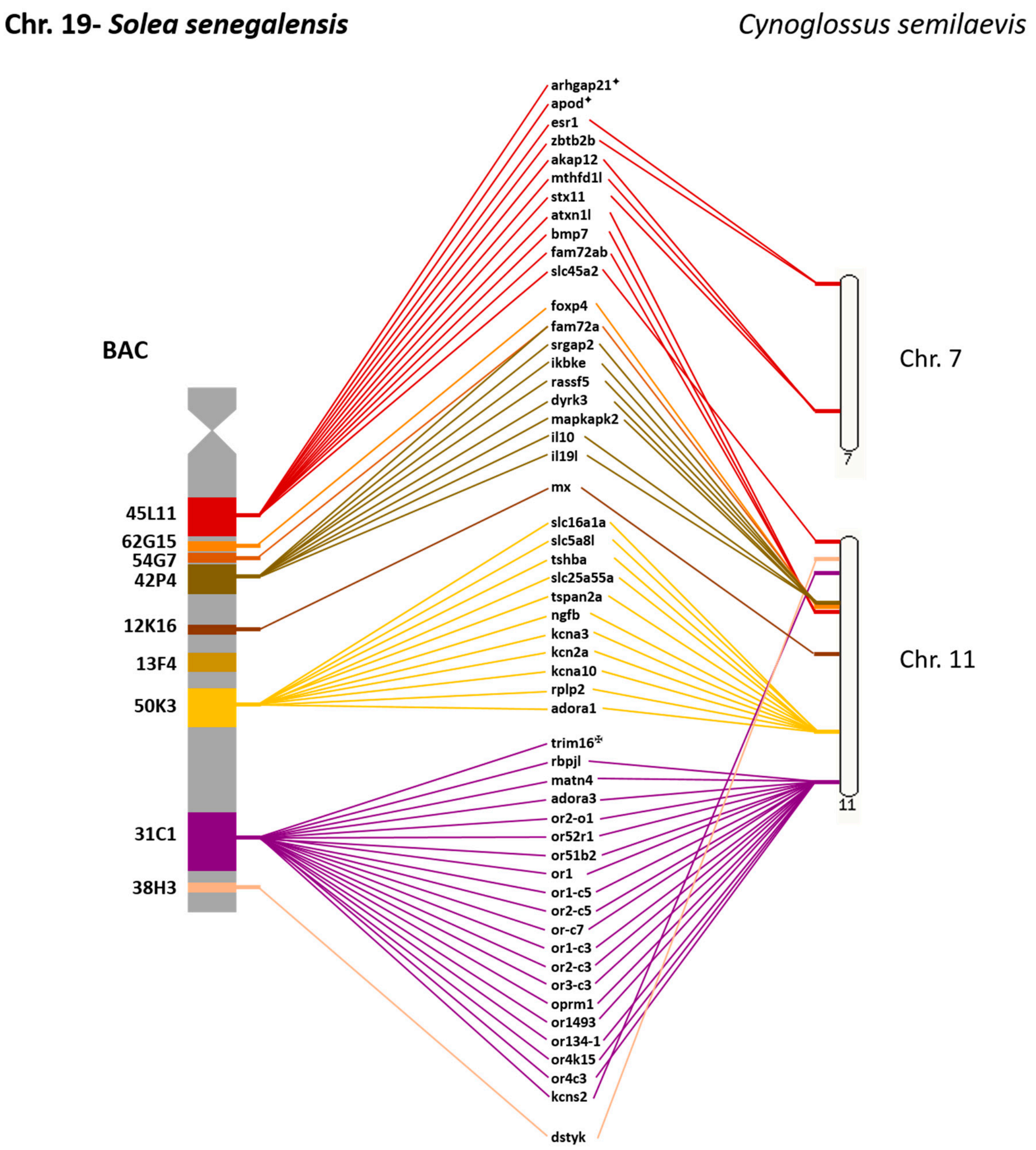

Figure S33. Syntenic map of the acrocentric chromosome 19 of *Solea senegalensis* with respect to *Cynoglossus semilaevis*. Legend symbols: ✦Genes found in chromosome 20 of *C. semilaevis*. ✠Gene found in chromosome 8 of *C. semilaevis*.

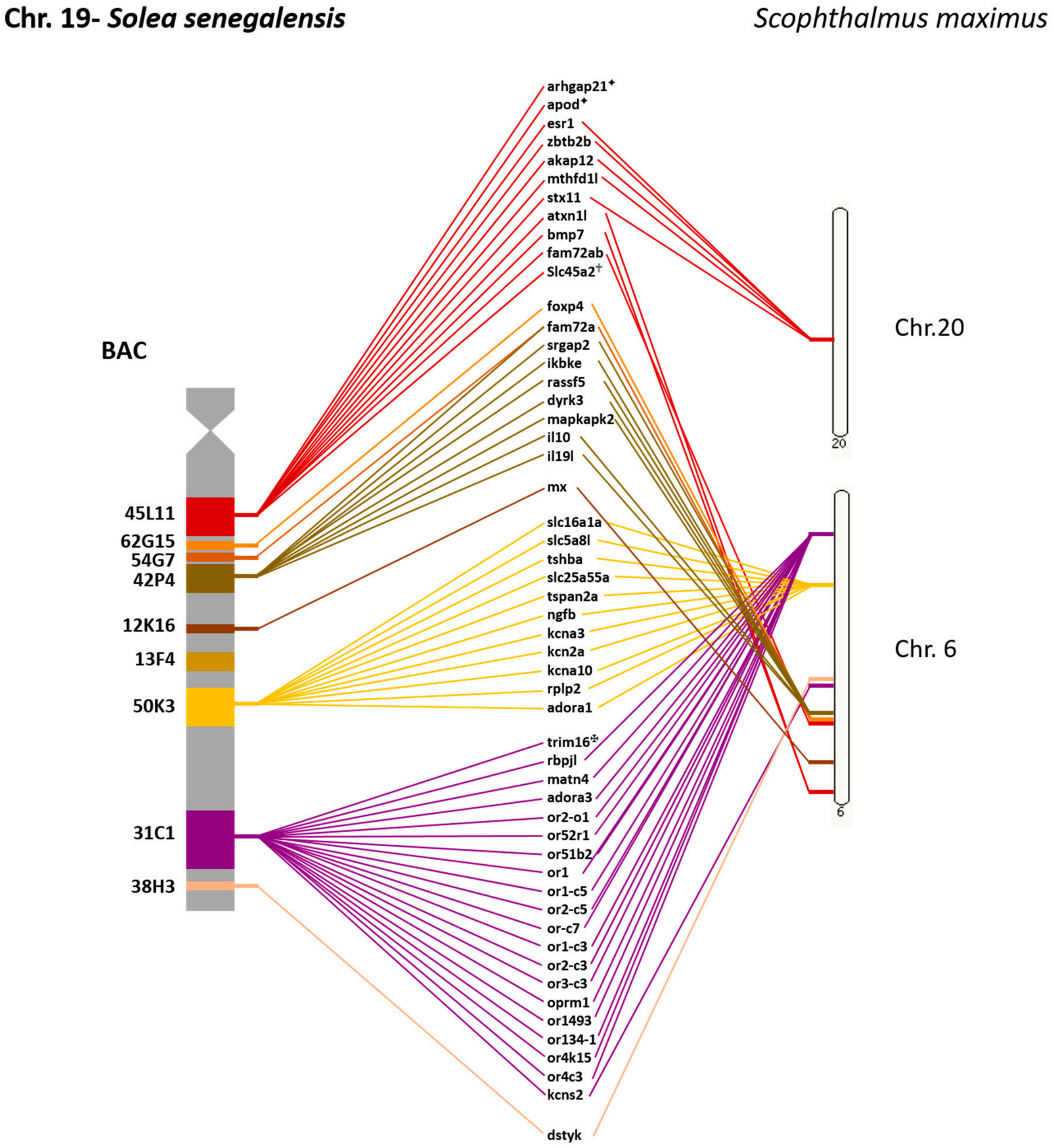

Figure S34. Syntenic map of the acrocentric chromosome 19 of *Solea senegalensis* with respect to *Scophthalmus maximus*. Legend symbols: ✦Genes found in chromosome 21 of *S. maximus*. †Gene found in chromosome 16 of *S. maximus*. ✠Gene found in chromosome 18 of *S. maximus.*

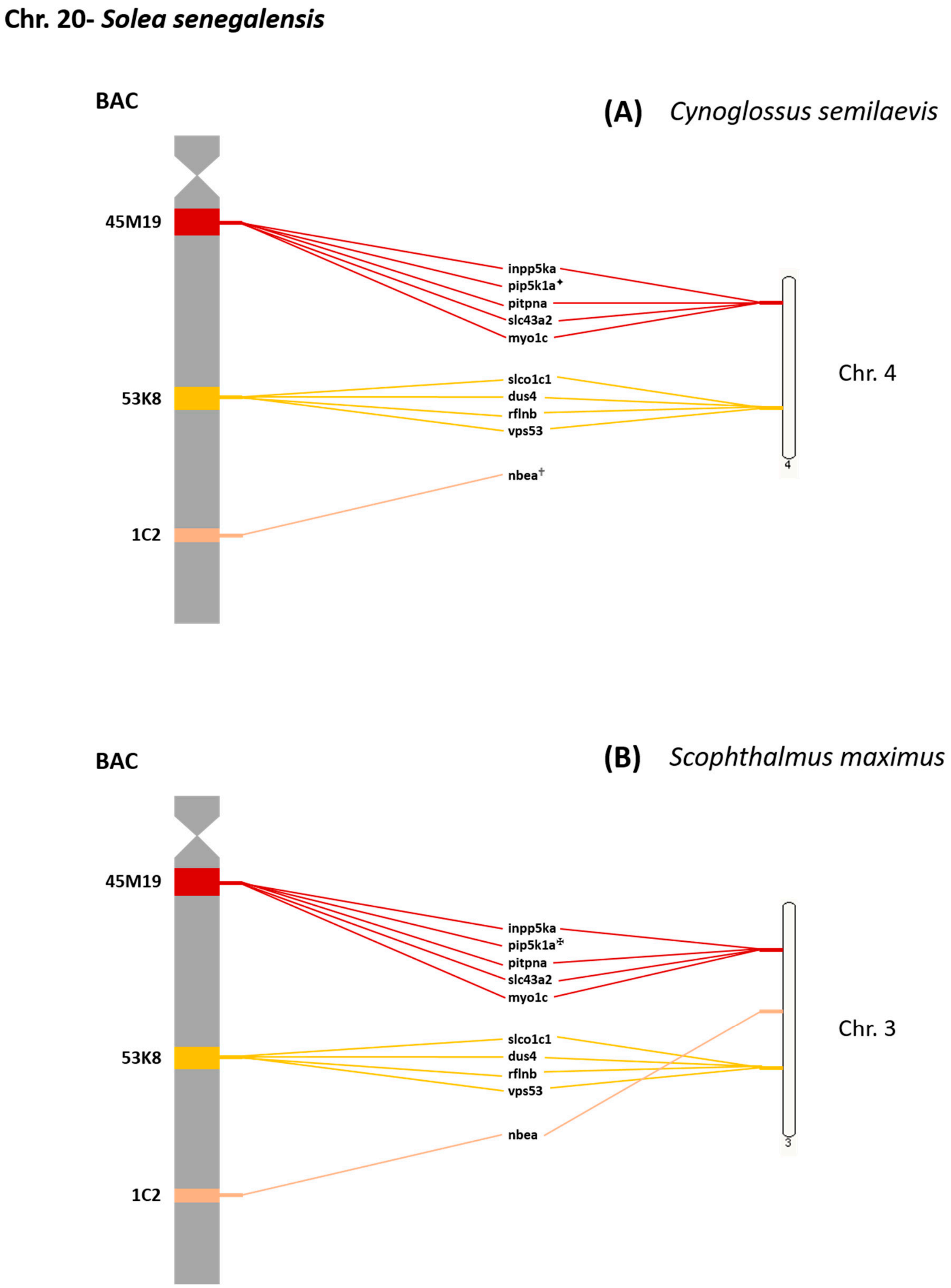

Figure S35. Syntenic map of the acrocentric chromosome 20 of *Solea senegalensis* with respect to (A) *Cynoglossus semilaevis* and (B) *Scophthalmus maximus*. Legend symbols: ✦Genes found in chromosome 18 of *C. semilaevis*. †Gene found in chromosome 19 of *C. semilaevis*. ✠Gene found in chromosome 22 of *S. maximus*.

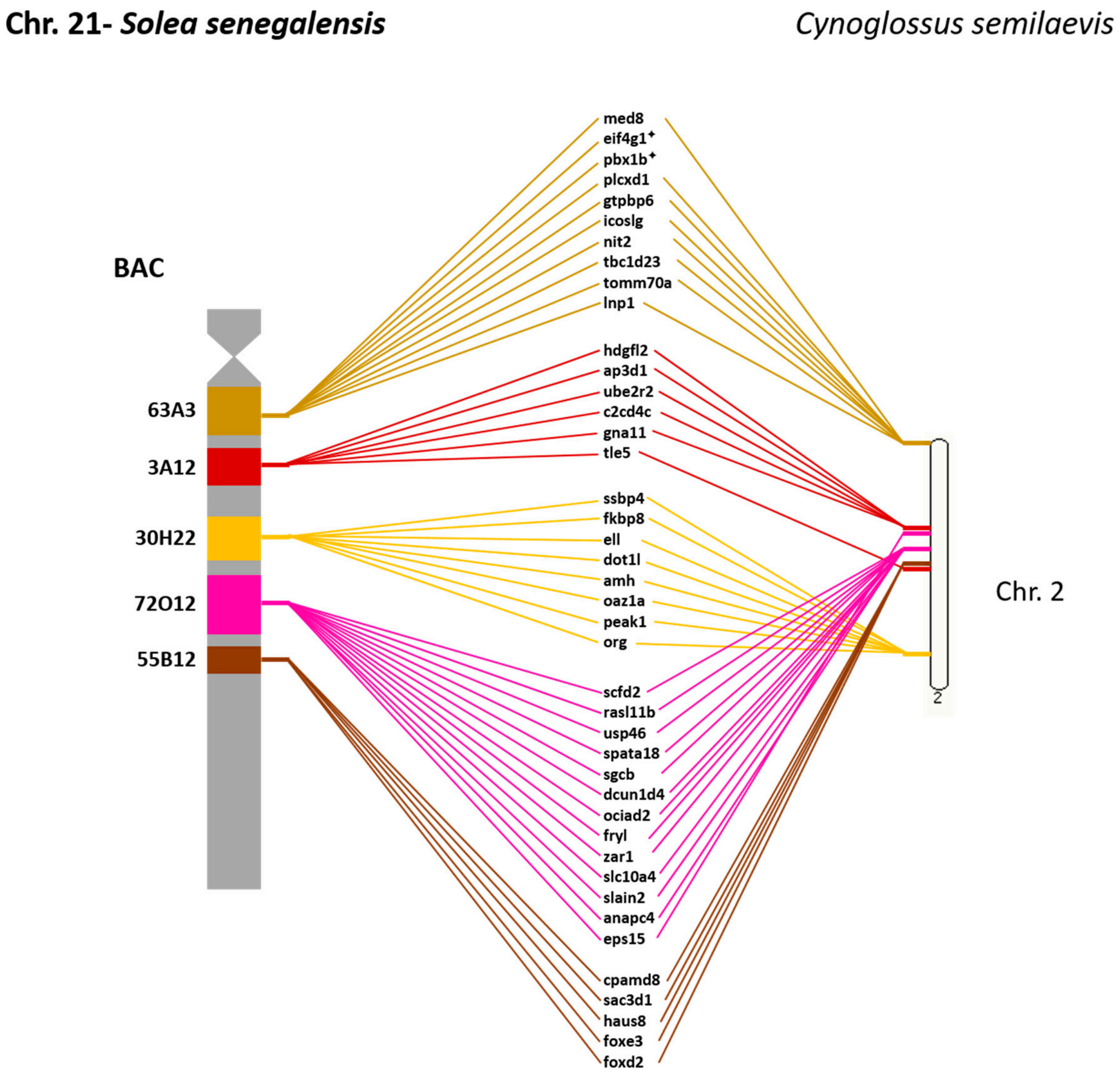

Figure S36. Syntenic map of the acrocentric chromosome 21 of *Solea senegalensis* with respect to *Cynoglossus semilaevis*. Legend symbols: ✦Genes found in chromosome 20 of *C. semilaevis*.

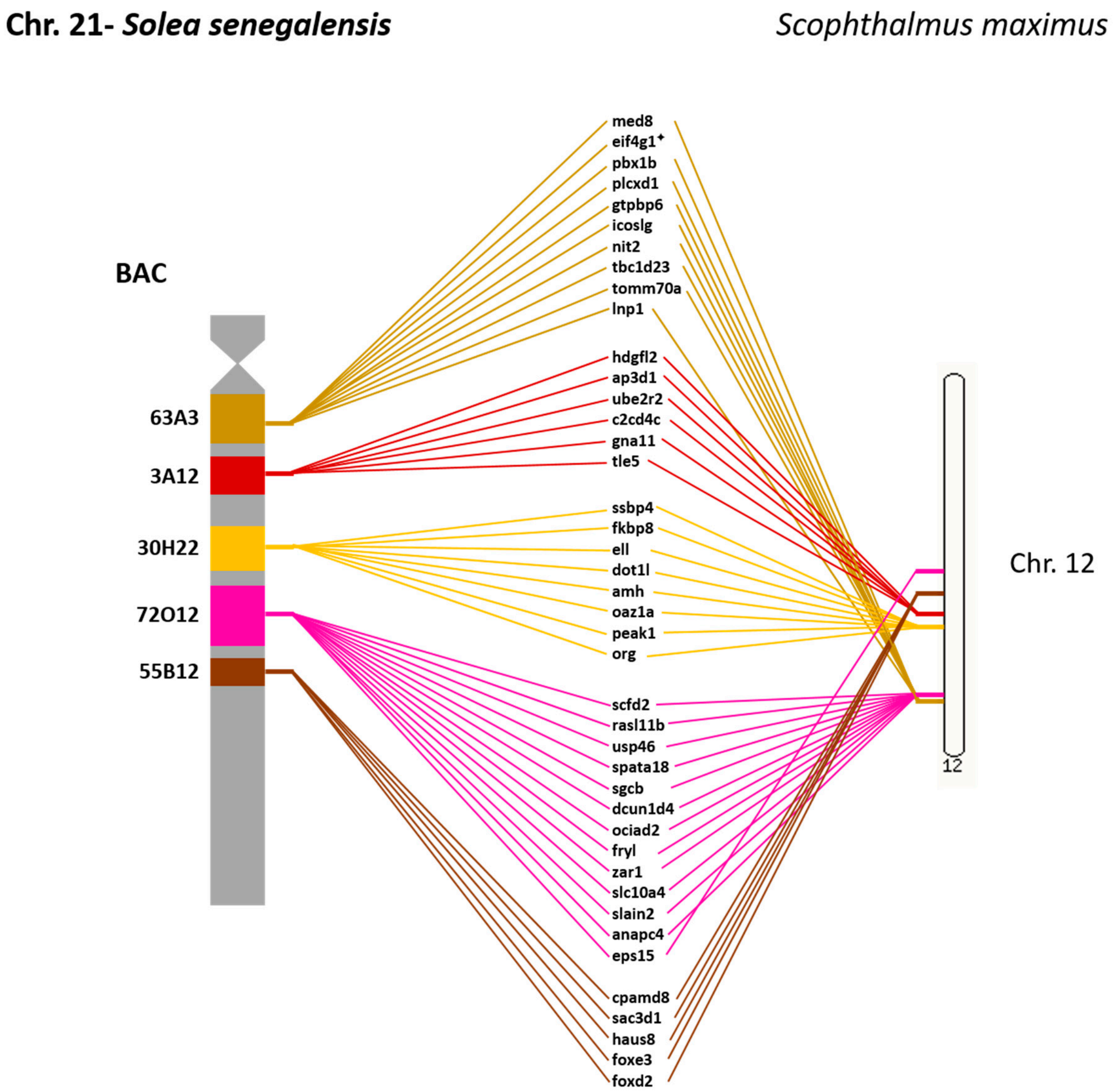

Figure S37. Syntenic map of the acrocentric chromosome 21 of *Solea senegalensis* with respect to *Scophthalmus maximus*. Legend symbols: ✦Gene found in chromosome 7 of *S. maximus*.

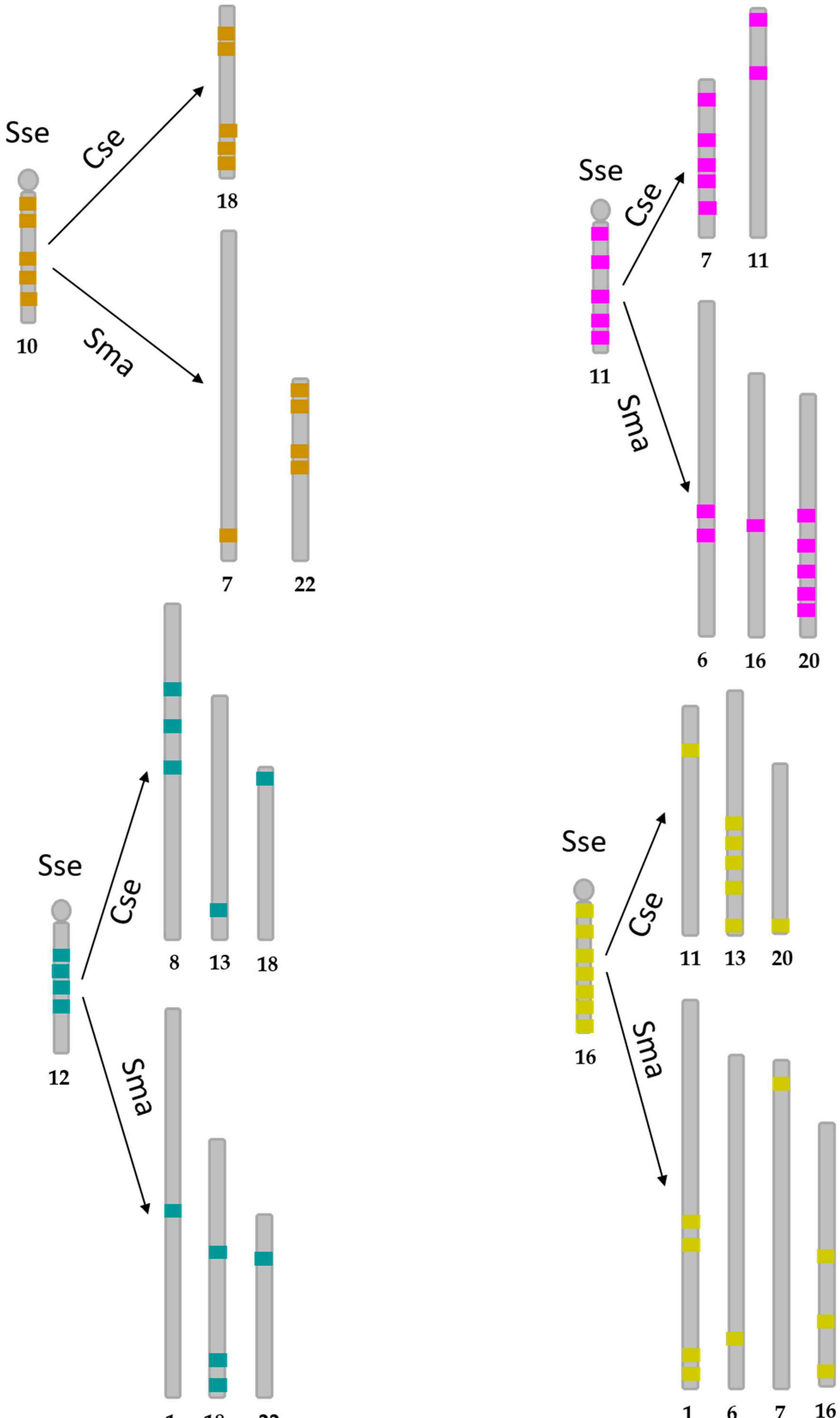
Sse
Cse
Sma
10
18
7
22
Sse
Cse
Sma
11
7
11
6
16
20
Sse
Cse
Sma
12
8
13
18
1
18
22
Sse
Cse
Sma
16
11
13
20
1
6
7
16

Figure S38. Distribution of BAC clones present on telocentric chromosomes 10, 11, 12 and 16 of *Solea senegalensis* throughout the chromosome complement of *Cynoglossus semilaevis* and *Scophthalmus maximus*.

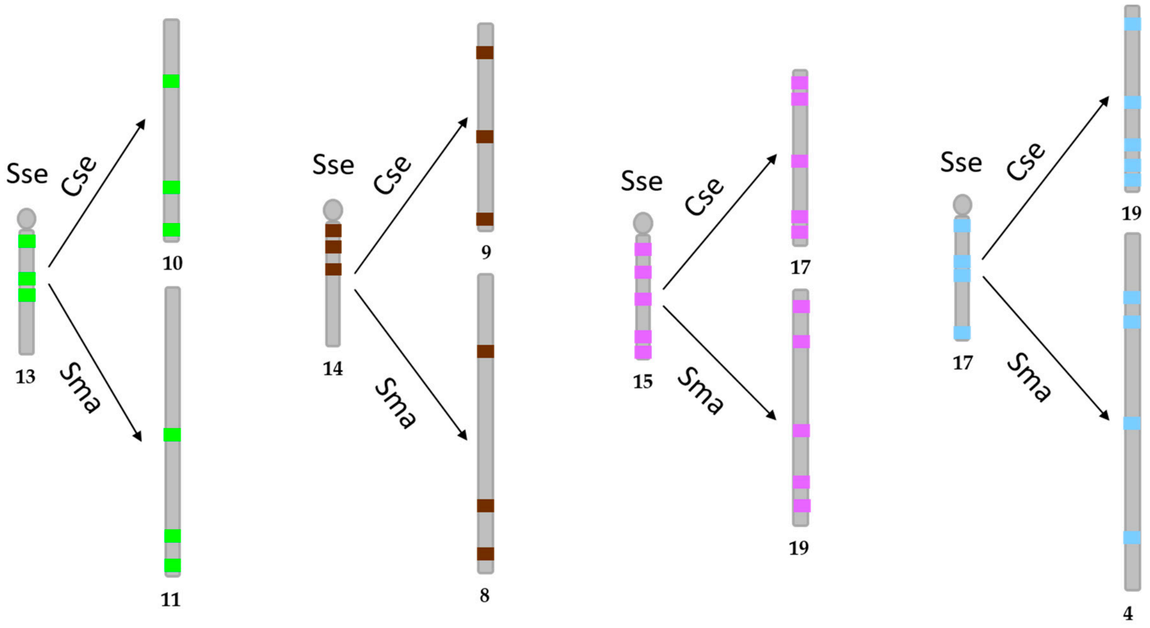

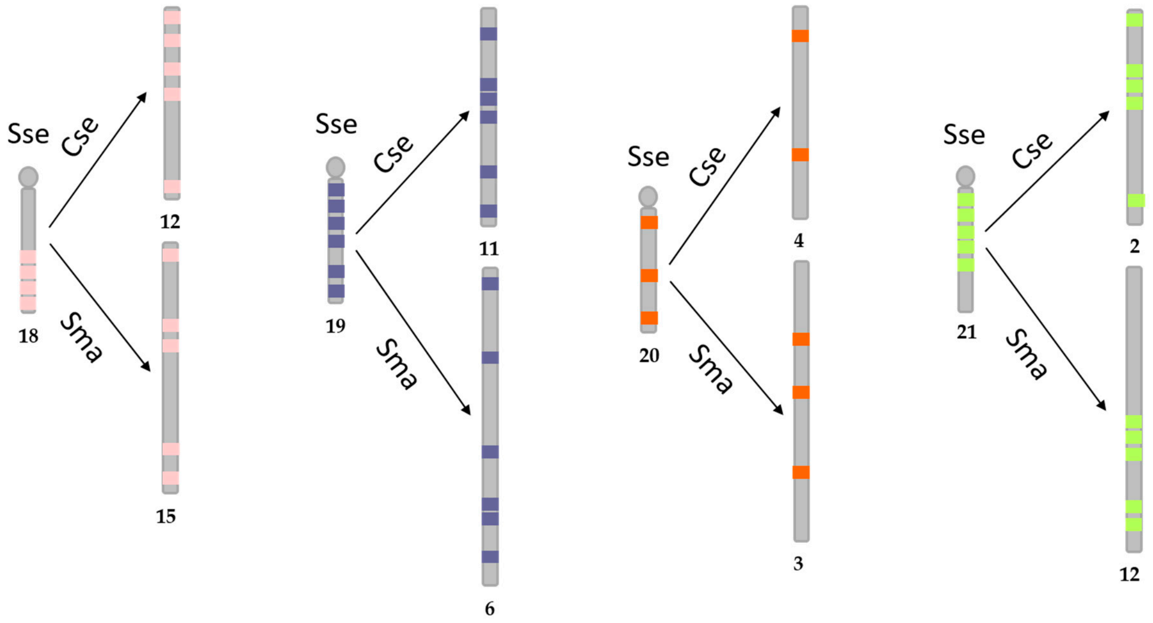

Figure S39. Orthologue chromosomes of *Cynoglossus semilaevis* and *Scophthalmus maximus* of the *Solea senegalensis* telocentric chromosomes 13-15, 17-21.
