# Supplementary material for: Integration of Maps Enables a Cytogenomics Analysis of the Complete Karyotype in Solea senegalensis
Source: Int J Mol Sci. 2022 May 11;23(10):5353. doi: 10.3390/ijms23105353 (PMC9140517; doi:10.3390/ijms23105353)
Supplement: Supplementary file 1 [file ijms-23-05353-s001.zip › Supplementary Materials Legends.pdf]

Supplementary Materials Legends

Table S1. References or NCBI Accession Number of all BAC clones used.
Legend symbol: *If newly reported.

Table S2. Primers used for microsatellite screening (included in Molina-Luzon et al. [29]) in the library of *Solea senegalensis*.
Legend symbol: *Linkage group described by Molina-Luzón et al. [29].

Table S3. BACs that in this work have changed their location in other chromosome, the position within the same chromosome, or have been deleted with respect to their previous location described by Merlo et al. [36].

Table S4. BAC sequencing results.
Legend symbol: *The BAC 31A2 is not included because it was previously described [22].

Table S5. Annotation data of all BAC clones used.

Table S6. *Cynoglossus semilaevis* orthologue localization of each BAC clones analyzed in *Solea senegalensis* and number of genes found within.
Legend numbers: [1]BAC 73B7 contains 3 genes present in BAC 10K23, [2]BAC 10L10 contains 4 genes present in BAC 5K5 and 2 genes shared with BAC 10K23, [3]Five of these genes belong to the histone cluster, [4]BAC 74M4 contains 2 genes present in BAC 72B11, [5]BAC 11020 contains 3 genes present in BAC 20D18, [6]BAC 16E16 contains 2 genes present in BAC 48K7, [7]BAC 13O12 overlaps in one gene with BAC 76F9, [8]BAC 4E10 contains 2 genes present in BAC 45L11, [9]BAC 13L18 overlaps in one gene with BAC 56H24, [10]BAC 54G7 contains 1 gene present in BAC 42P4, [11]BAC 19K18 overlaps in one gene with BAC 65I16.

Table S7. *Scophthalmus maximus* orthologue localization of each BAC clones analyzed in *Solea senegalensis* and number of genes found within.
Legend numbers: [1]BAC 73B7 contains 3 genes present in BAC 10K23, [2]BAC 10L10 contains 4 genes present in BAC 5K5 and 2 genes shared with BAC 10K23, [3]Five of these genes belong to the histone cluster, [4]BAC 74M4 contains 2 genes present in BAC 72B11, [5]BAC 11020 contains 3 genes present in BAC 20D18, [6]BAC 16E16 contains 2 genes present in BAC 48K7, [7]BAC 13O12 overlaps in one gene with BAC 76F9, [8]BAC 4E10 contains 2 genes present in BAC 45L11, [9]BAC 13L18 overlaps in one gene with BAC 56H24, [10]BAC 54G7 contains 1 gene present in BAC 42P4, [11]BAC 19K18 overlaps in one gene with BAC 65I16, [12]BAC 68P5 contains 7 genes present in BAC 72O12.

Table S8. Number of *loci* per Mb (NL/Mb) and coverage (%) of repeat elements in BACs from chromosomes 3,5-9, 11-12 and 16 of *S. senegalensis*. Repeat classes: DNA transposons, retroelements, small RNA, satellites, simple repeats, low complexity.

Table S9. Abundance (measured as NL/Mb and Coverage) of TE families in BACs from chromosomes 3,5-9, 11-12 and 16 of *Solea senegalensis*.

Table S10. Summary of the most relevant results obtained after analysis of repeated sequences carried out in BACs mapped along eight chromosomes in *Solea senegalensis.*

Figure S40. Analysis or repeat elements in BACs mapped in chromosomes 3, 5-9, 11-12 and 16 from the flatfish *Solea senegalensis.*

Figure S40.1. Number of *loci* per Mb (NL/Mb) of repeat elements in BACs from chromosomes 3,5-9, 11, 12 and 16 of *Solea senegalensis*. (**a**) DNA transposons, (**b**) retroelements, (**c**) small RNA, (d) satellites, (**e**) simple repeats, (**f**) low complexity.

Figure S40.2. Coverage (%) of repeat elements in BACs from chromosomes 3,5-9, 11, 12 and 16 of *Solea senegalensis*. (**a**) DNA transposons, (**b**) retroelements, (**c**) small RNA, (d) satellites, (**e**) simple repeats, (**f**) low complexity.

Figure S40.3. Abundance (measured as NL/Mb and Coverage) of TE families in BACs from chromosome 3 of *Solea senegalensis*.

Figure S40.4. Abundance (measured as NL/Mb and Coverage) of TE families in BACs from chromosome 5 of *Solea senegalensis*.

Figure S40.5. Abundance (measured as NL/Mb and Coverage) of TE families in BACs from chromosome 6 of *Solea senegalensis*.

Figure S40.6. Abundance (measured as NL/Mb and Coverage) of TE families in BACs from chromosome 7 of *Solea senegalensis*.

Figure S40.7. Abundance (measured as NL/Mb and Coverage) of TE families in BACs from chromosome 8 of *Solea senegalensis*.

Figure S40.8. Abundance (measured as NL/Mb and Coverage) of TE families in BACs from chromosome 9 of *Solea senegalensis*.

Figure S40.9. Abundance (measured as NL/Mb and Coverage) of TE families in BACs from chromosome 11 of *Solea senegalensis*.

Figure S40.10. Abundance (measured as NL/Mb and Coverage) of TE families in BACs from chromosome 12 of *Solea senegalensis*.

Figure S40.11. Abundance (measured as NL/Mb and Coverage) of TE families in BACs from chromosome 16 of *Solea senegalensis*.

Figure S40.12. Boxplot summarizing the number of *loci* per Mb (NL/Mb) and coverage (%) of repeat elements in chromosomes 3,5-9, 11, 12 and 16 of *Solea senegalensis* inferred from clon BACs analysis.

Figure S40.13. Average abundance (measured as NL/Mb and Coverage) of TE families in chromosomes 3, 5-9, 11-12 and 16 of *Solea senegalensis* inferred from BAC analysis.
