# Supplementary material for: Integration of Maps Enables a Cytogenomics Analysis of the Complete Karyotype in Solea senegalensis
Source: Int J Mol Sci. 2022 May 11;23(10):5353. doi: 10.3390/ijms23105353 (PMC9140517; doi:10.3390/ijms23105353)
Supplement: Supplementary file 1 [file ijms-23-05353-s001.zip › Table S10_New Seq rep.pdf]

Table S10. Summary of the most relevant results obtained after analysis of repeated sequences carried out in BACs mapped along eight chromosomes in *Solea senegalensis*.

Chromosome number	Repetitive element distribution in the chromosome	Figures / Tables
3	- Large number of DNA transposon *loci* in telomeric BACs, specifically the hobo-Activator family.<br>- Large number of simple repeat loci in the BACs mapped in p arm of this metacentric chromosome.<br>- BAC 9C12 (pericentromeric): presents the largest numbers of low complexity *loci* of all the BACs analysed in this work.<br>- Large numbers of repeat elements in telomeric BACs (54H18 and 4B13): mainly DNA transposons<br>- The SSR coverage of the p arm of the ideogram is greater than in the q arm.<br>- High coverage values of LTR retrotransposons in BAC 54H18 as well as the Gypsy/DIR family.	Figure 3<br>Figure S40.1<br>Figure S40.2<br>Figure S40.3<br>Table S8<br>Table S9.
5	- BAC3I18: Large number of *loci* with repeated elements, found in all types of repeats except satellites.<br>- Lower levels of coverage in BAC 74M4 than in BAC 31I18, in main repeat elements, except at the family level, with larger numbers in hobo-Activator in 74M4.	Figure 3<br>Figure S40.1<br>Figure S40.4<br>Table 7<br>Table S9
6	- Globally homogeneous in repeated sequences.<br>- Notably very large numbers of satellites in BAC 64A8 (p arm).<br>- BACs 48K7 and 16E16: many simple repeat *loci* in relation to the rest of the BACs.<br>- BAC 16E16: The number of PiggyBac and Gypsy/DIRS1 transposon DNA elements is very large.<br>- BAC 64A8, mapped together with rDNA 5S, in the p arm: very high values of satellite coverage in relation to the rest of the BACs and also it has very large numbers of LINEs and L2/CR1/Rex type retrotransposons.	Figure 3<br>Figure S40.1<br>Figure S40.5<br>Table S8<br>Table S9

7	- BAC 47G8 globally has the highest repeated elements number.<br>- BACs grouped in subtelomeric positions of the q arm present large numbers of repeated elements.<br>- BACs 44K21, 39D10 and 47G8, are those with the largest NL/Mb numbers of DNA transposon elements.<br>- BAC 19H9 has the largest numbers of simple repeats.<br>- Increasing numbers of hobo-Activator elements are observed closer to the telomere of the q arm.<br>- BAC 19H9 shows very high levels of coverage of simple repeats.<br>- BAC 76F9 region shows the low-complexity sequences category, the largest numbers in this study.<br>- Hobo-Activator presents higher values of coverage closer to the telomere.<br>- BACs 13O12 and 47G8 stand out for their high coverage in LINES and L2/CR1/Rex.	Figure 3<br>Figure S40.1<br>Figure S40.6<br>Figure S40.1<br>Table S8<br>Table S9
8	- BACs 46P22 and 31A2: largest numbers of repeated element *loci* and high coverage by the presence of many retroelements and DNA transposons.<br>- BAC 46P22 stands out for its large number of satellites, SSRs and low complexity.<br>- BAC 57C10: The lowest number of repeated sequences.<br>- BACs 31A2 and 46P22: The most abundant in LINES and L2/CR1/Rex retrotransposons and hobo-Activator DNA transposon elements.<br>- BAC 46P22, and to a lesser extent BAC 31A12, show the highest coverage values for hobo-Activator, LINEs and L2/CR1/Rex elements.	Figure 3<br>Figure S40.1<br>Figure S40.7<br>Table S8<br>Table S9
9	- Homogeneous number of repeated elements (*loci* per Mb) among all the BACs.<br>- BAC 4N9: large numbers of retroelements, DNA transposon and small numbers of SSRs and low complexity sequences.<br>- Subcentromeric BAC 32B8: very large numbers for low complexity elements.<br>- BAC 39F2: The largest numbers of hobo-Activator and LINEs families.<br>- BACs 4N9 and 51E10: highest values at the coverage level. The former in retroelements (LINEs, L2/C1/Rex and Gypsy/DIRS1 elements) and DNA transposons, while the latter, for LTR and satellites.<br>- BAC 32B8: very large numbers of low complexity sequences.	Figure 3<br>Figure S40.2<br>Figure S40.8<br>Table S8<br>Table S9

11	- BAC 31F1, 45L11 and 72B11: the largest numbers of DNA transposons (specifically hobo-Activator).<br>- LTR elements are most abundant in the subtelomeric BAC, close to the 5S rDNA.<br>- The largest numbers of LINEs are found in the most interstitial BACs, such as 31F1 and 45L11.<br>- LINE and L2/CR1/Rex elements are very abundant in BACs 45L11 and 30N10.<br>- BAC 31F1: abundant in LTRs and gypsy/DIRS1 elements	Figure 3<br>Figure S40.9<br>Figure S40.1<br>Table S8<br>Table S9
12	- All BACs analysed in this chromosome have a high global abundance of repeated elements (> 2000 NL/Mb), with many DNA transposons loci in all of them.<br>- Hobo-Activator elements present the largest numbers in BACs 13F2 and 13E1.<br>- LINEs and L2/C1/Rex are more abundant in centromeric BAC 13F2, with the largest number of Tc1-IS630-Pogo elements in BAC 13E1.<br>- Centromeric BAC 13F2: High coverage of retroelements<br>- BACs 38B21, 57N7 and 35D17: large numbers of simple repeats.<br>- The most abundant families are LINEs and L2/CR1/Rex, observed in BAC 13F2.<br>- Hobo-Activators are found in large numbers in all BACs.	Figure 3<br>Figure S40.1<br>Figure S40.10<br>Table S9
16	- BACs 76A22, 57N7, 9N8, 53D20 and 71N11: many repeated elements loci.<br>- BACs 52E17, 25P16 and 54E18: low abundance of repeated elements.<br>- BAC 71N11: SSRs shows the largest abundance (NL/Mb) in this work.<br>- BAC 53D20 has the highest numbers of hobo-activator, followed by BAC 71N11.<br>- LINEs and L2/C1/Rex are not very abundant in the most central BACs, such as 25P16 and 54E18.<br>- The telomeric BAC 76A22 presents the largest numbers in this study, with large numbers of retroelements and DNA transposons, although with low numbers of simple repeats and low complexity elements.<br>- 71N11: The highest SSR coverage values found in this telomeric BAC.<br>- BAC 76A22 shows very high numbers of LINEs and L2/C1/Rex, as well as L1/CIN4 (retrotransposon) elements.	Figure 3<br>Figure S40.1<br>Figure S40.2<br>Figure S40.11<br>Table S8<br>Table S9
