# Supplementary material for: Integration of Maps Enables a Cytogenomics Analysis of the Complete Karyotype in Solea senegalensis
Source: Int J Mol Sci. 2022 May 11;23(10):5353. doi: 10.3390/ijms23105353 (PMC9140517; doi:10.3390/ijms23105353)
Supplement: Supplementary file 1 [file ijms-23-05353-s001.zip › Table S1_modified.pdf]

Table S1. References or NCBI Accession Number of all BAC clones used.

BAC clone	BAC Chromosome location	References / NCBI Accession Number*
1C2	20	[32]
2F9	13	Present work / OK474331
2K18	18	[31]
3A12	21	Present work / OK474325
3C15	4	[34]
3F15	18	[36]
3I18	5	Present work / OK504493
3N10	11	[36]
4B13	3	Present work / OK504495
4D15	2	[32]
4E10	11	[34]
4F12	15	[34]
4M14	13	[38]
4N21	14	[39]
4N9	9	Present work / OK474327
5K5	1	[30]
6P22	14	[31]
7H22	7	[31]
8A23	4	[34]
8O7	8	[31]
9C12	3	Present work / OK474332
9E8	10	[19]
9J4	3	[34]
9N8	16	[19]
10F5	1	Present work / OK474302
10K23	1	[31]
10L10	1	[32]
11O20	6	[31]
12D22	1	[30]
12D24	4	[40]
12K16	19	[19]
12N15	4	[31]
13E1	12	[19]
13F2	12	[34]
13F4	19	[34]
13G1	1	[32]
13L18	10	Present work / OK504496
13O12	7	Present work / OK474328
15B1	18	Present work / OK504494

15I19	1	[34]
16E16	6	[31]
19H9	7	[31]
19J21	2	[31]
19K18	17	[36]
19L16	13	[36]
20D18	6	[31]
21I14	2	Present work / OK474333
21O23	2	[31]
22C2	15	[19]
25P16	16	[38]
29D4	14	[41]
30H22	21	[31]
30P17	4	[34]
31A1	6	[34]
31A2	8	[34]
31C1	19	[41]
31F1	11	[34]
31N1	17	[34]
32B8	9	[31]
35D17	12	[34]
36D3	1	[32]
36E3	15	[34]
36H2	4	[34]
36H3	4	[34]
36I3	2	[34]
36J2	4	[34]
36K1	2	[34]
36M2	18	[34]
38B21	12	Present work / OK474330
38F24	11	[37]
38H3	19	Present work / OK504492
38N10	2	[41]
39D10	7	Present work / OK504487
39F2	9	[38]
39G22	4	Present work / OK474315
42D4	2	Present work / OK337834
42F9	17	Present work / OK504497
42P4	19	[41]
44K21	7, 15	Present work / OK392607
45L11	11, 18, 19	[36]
45M19	20	Present work / OK474314
46B2	4	[34]
46C5	2	[34]

46P22	8	[41]
47B18	6	Present work / OK474329
47G8	7	Present work / OK474334
48K7	6	[32, 33]
48P7	1	[32, 33]
50K3	19	[38]
51E10	9	Present work / OK474335
52C17	1	[32, 33]
52E17	16	[34]
52G10	2	[41]
53D20	16	[41]
53K8	20	Present work / OK474313
54E18	16	Present work / OK474307
54G7	19	Present work / OK474326
54H18	3	Present work / OK504488
55B12	21	[38]
56H24	10	[32]
57C10	8	Present work / OK392608
57G16	9	Present work / OK392609
57N7	10, 12, 16	Present work / OK474308
60P19	2	[38]
60P24	15	[36]
62G15	19	Present work / OK392610
63A3	21	[19, 41]
63A7	17	[19]
64A8	6	[36]
65E23	2	Present work / OK392611
65I16	17	[36]
65J17	13	Present work / OK474306
67K3	4	Present work / OK474312
67N4	1	Present work / OK474311
67P21	1	Present work / OK474310
67P7	6	Present work / OK474304
68G4	10	[36]
68P5	2	Present work / OK504489
71N11	16	[41]
72B11	5, 9, 11	Present work / OK392613
72O12	21	Present work / OK474305
73A11	3, 14	Present work / OK474303
73B7	1	[32, 33]
74M4	5	Present work / OK392614
76A22	16	Present work / OK474309
76F9	7	Present work / OK392615

*If newly reported.
