# Supplementary material for: Integration of Maps Enables a Cytogenomics Analysis of the Complete Karyotype in Solea senegalensis
Source: Int J Mol Sci. 2022 May 11;23(10):5353. doi: 10.3390/ijms23105353 (PMC9140517; doi:10.3390/ijms23105353)
Supplement: Supplementary file 1 [file ijms-23-05353-s001.zip › Table S2.pdf]

Table S2. Primers used for microsatellite screening (included in Molina-Luzon et al. [29]) in the library of *Solea senegalensis*.

BAC clone	Reference	LG of origin*	Primer sequences 5′→ 3′		Annealing Tª (ºC)
			Fw	Rv	
**2F9**	*MSS-162*	LG10	AGTGACGCAGCGGTAATAGC	ACGCACTGTGACCTCAACCT	61
**3A12**	*MSS-056*	LG8	GCTCAGCTGCATGTGCTTT	TGGTCACTTCAGAACTCCACTC	60
**31A2**	*Mss-22*	LG6	CGCCAGGTTGTTCAAACACT	TTTGTCAGTCGTCCTCCAGA	57
**3I18**	*SSENEG123000*		TCCTTCACAGCATTGAGTCG	GCTGCCAAACAGGAGAACAT	58
**4B13**	*SSENEG8782*		AAGCACCTAACGGAATCTGC	GTGTCTGACCGACATTTTGGA	58
**4N9**	*1NO7*	LG13	TGCACATCAGTGAGTTAATATT	TTGTGATGGCGTGAAAAGTTC	56
**9C12**	*EST-60*	LG15	AAGCAAACATTCATCCGTCA	ATTGATTTACCCAACAGCGTCT	56
**13L18**	*SSENEG7074*		TCATCGGCTAATCACATCCA	GTTTCACCGACACTTTGAACCTGA	59
**13O12**	*MSS-137*	LG3	TCATCCATTCAGTTTCCCATT	GGCGTGTGTTGTCTGTATGC	58
**15B1**	*SSENEG3415*		ACCGCTGGGATGTACTGAAG	GCCTGTCCATTGTGAGGAAT	59
**21I14**	*EST-34*	LG7	ATCGGTCAAACGCAGAAGAG	TGGACATATGGCACCCTAAA	57
**38B21**	*EST-39*	LG19	GGGAGAGTGCAGAACTCCAG	TCCCCGCTATTGAGATGTTC	60
**38H3**	*MSS-066*	LG2	ACTCTTTAACAAGTAAACCTGCATTA	CATTTAACATGGATGAAACAGCA	58
**39D10**	*CSSE2H15*	LG3	ACCAAAGTAGCGCAGATTCC	CTTCATCAGCAGCCAAACTG	58
**39G22**	*MSS-037*	LG18	AAAGGCTGAATTAGCTTTGAACA	GCATGACTCTGCCGTGACT	58
**42D4**	*MSS-075*	LG15	CAGGTCAGCGTCTGTTTGAG	TGCACTGAGGGCTTCTCTTT	59
**42F9**	*SSENEG7666*		TGGGCAGGAAGTCAGCTAAA	GTTTCTGTGAACCCAGGTTTCCTT	60
**44K21**	*MSS-108*	LG14	TTGAACCCTGTTCTCGAGGT	GACGCAACAAACCAAAGATG	57
**45M19**	*EST-47*	LG22	TGGGAGAGGTCAGGAGTACG	AGACTTCACACCGGGATCAG	61
**47B18**	*MSS-091*	LG27	GAGTATTTTGAACTGACCAAACCTC	CCAACAGAACACGAAGCAAA	59
**47G8**	*MSS-063*	LG4	ACATTTCATTTGCTGCTGCCACA	GGGACATGTTGGCTGATTCT	60
**51E10**	*MSS-111*	LG1	TCGACCTCTTATGGCTGACTG	CGGTGAAAGCAGAGGGAAAG	61
**53K8**	*MSS-081*	LG24	GCTGGTCTATTTGGAGGGAAG	CTCATTAGCGCATCACAGGA	59
**54E18**	*MSS-043*	LG16	AAATGGGCTTCGCTGCTTA	TCGGAGGTGGGATTAACAAG	56
**54G7**	*MSS-110*	LG2	TCTAATTCACCGTGCAAACTG	TGAAATAGAAGCAGCCGTGA	57
**54H18**	*MSS-80*	LG4	GCTCGCTCTAAATGGCTTTC	TCATTTGACAGAGTGAACTTGG	58
**57C10**	*MSS-001*	LG6	TGTCATTGAAGGGTGCACTAA	AAACAACTTTTGCACGGTGA	55
**57G16**	*EST-12*	LG13	AAGATAACCCCCGTGTTGTG	GACCGTTAAAACTCCCCACAT	59
**57N7**	*MSS-129*	LG19	TTGGCAGACATGAACCAAAA	CCCCAAAGCCACAGAGTCTA	57
**62G15**	*MSS-131*	LG2	AAAAGTTCGCCGTAGGGTTT	GAACGCTGACTTTTGCATCA	56
**65E23**	*MSS-130*	LG12	CAGTGCATCATTATGGGGTTT	AGAGGCACGCAATTCTCTGT	57

**65J17**	*MSS-057*	LG10	ATTTGCCAGGAACACGAATC	AGGAGAACCCTGAATCCACA	57
**67K3**	*MSS-062*	LG25	ATTCATGCCGACGACCTACT	CGGAGACCATGAGCTGAAAT	58
**67N4**	*EST-5*	LG27	ATGCCAATAAACCTGGCACT	GAACCGATCCCAACTGTGTT	57
**67P21**	*MSS-014*	LG21	ACGTGAGAGGAAGTGGTGCT	CGCCTCCAATGTCAGATTTT	58
**67P7**	*MSS-044*	LG4	TTGGCATGATTTGGCAGTT	CAGTTGGGCAACCTATTATTTGA	56
**68P5**	*EST-23*	LG7	CTCAGCCTCTCCTTCATTCC	CAGTTTGCGCTGACAACATAA	59
**72B11**	*MSS-079*	LG11	GGTATCCAGGTGGAAGGTCA	GTGCCTGGAAATGACAGTGA	59
**72O12**	*MSS-050*	LG8	GCTGGGTTCGAGTTACAAGC	GACTTGCTTATCCTTACATTCA	58
**73A11**	*MSS-065*	LG1	TGGGATCAAATGAAGTCAGAAA	TTGTGCAATATCACGAATGGA	56
**74M4**	*MSS-061*	LG9	CAGTGGAACAATAACGTGTGG	GGAGCGGTTATGAACTGACC	59
**76A22**	*EST-15*	LG20	TGCAAAAAGTTGAGGCTCATAA	TCCTGGACTGTTTTTCACTTTG	57
**76F9**	*MSS-121*	LG3	AGTGGCTCAGAAGAAACCACA	CTTCCACGGAGAAGTCGGTA	59

*Linkage group described by Molina-Luzón et al. [29].
