# Supplementary material for: Integration of Maps Enables a Cytogenomics Analysis of the Complete Karyotype in Solea senegalensis
Source: Int J Mol Sci. 2022 May 11;23(10):5353. doi: 10.3390/ijms23105353 (PMC9140517; doi:10.3390/ijms23105353)
Supplement: Supplementary file 1 [file ijms-23-05353-s001.zip › Table S3_New.pdf]

Table S3. BACs that in this work have changed their location in other chromosome, the position within the same chromosome, or have been deleted with respect to their previous location described by Merlo et al. [36].

	Merlo et al. [36]	Present work
**BACs**	**BAC Chromosome location**	**BAC Chromosome location**
48K7	1p proximal/subcentromeric and 6q interstitial	6q distal/subtelomeric
16E16	1p proximal/subcentromeric, 6q proximal/subcentromeric and 15q interstitial	6q distal/subtelomeric
53B20	1p proximal/subcentromeric	Eliminated
54H24	1p subcentromeric	10q subcentromeric
1C2	1q telomeric	20q distal/subtelomeric
6P22	2p distal/subtelomeric	14q proximal/subcentromeric
9E8	2p interstitial and 10q subcentromeric	10q interstitial
19J21	2q proximal/subcentromeric and 15q interstitial	2q distal/subtelomeric
3F15	2q proximal/subcentromeric, 14q and 17q distal/subtelomeric	18q telomeric
19L16	2q distal/subtelocentric and 13q telomeric	13q interstitial
21O23	2q distal/subtelomeric and 14q telomeric	2p proximal/subcentromeric
9J4	3p distal/subtelomeric	3q interstitial
46B2	4p telomeric	4q interstitial
3C15	4p distal/subtelomeric	4q subcentromeric
8A23	4q proximal/subcentromeric	4q interstitial
12N15	4q proximal/subcentromeric	4p interstitial
30J4	4q proximal/subcentromeric	Eliminated from 4q proximal/subcentromeric and 12q telomeric
46P22	4q and 8q proximal/subcentromeric	8q interstitial
36H2	It was not included	4q telomeric
64A8	5q proximal/subcentromeric	6p subcentromeric
20D18	6q telomeric	6q proximal/subcentromeric
11O20	6q telomeric	6q proximal/subcentromeric
8O7	7q proxmal/subcentromeric	8p proximal/subcentromeric
31A2	8q distal/subtelomeric	8q proximal/subcentromeric
31A1	8q telomeric	6q interstitial
39F2	9p distal/subtelomeric	9q proximal/subcentromeric
19K18	9q proximal/subcentromeric, 16q and 17q interstitial	17q interstitial
15I19	10q proximal/subcentromeric	10q interstitial
68G4	10q proximal/subcentromeric	10q interstitial
38F24	11q interstitial	11q distal subtelomeric
45L11	11q interstitial	Also in 18q and 19q proximal/subcentromeric
13F2	12q proximal/subcentromeric	12 centromeric
35D17	12q telomeric	12q distal/subtelomeric

29D4	13q proximal/sucentromeric	14q proximal/subcentromeric
4N21	13q interstitial	14q interstitial
8O7	13q distal/subtelocentric	8p proximal subcentromeric
36E3	15q proximal/subcentromeric	15q distal/subtelomeric
9N8	15q and 16q distal/subtelomeric	16q distal/subtelomeric
22C2	15q telomeric	15q distal/subtelomeric
30P17	16q interstitial	4q proximal/subcentromeric
71N11	16q distal/subtelomeric	16q telomeric
53D20	16q telomeric	16q distal/subtelomeric
25P16	17q and 21q distal/subtelomeric	16q interstitial
12K16	19q proximal/subcentromeric	19q interstitial
13F4	19q proximal/subcentromeric	19q interstitial
50K3	19q interstitial	19q distal/subtelomeric
4M14	20q interstitial	13q proximal/subcentromeric
30H22	20q distal/subtelomeric	20q interstitial
