# Supplementary material for: Integration of Maps Enables a Cytogenomics Analysis of the Complete Karyotype in Solea senegalensis
Source: Int J Mol Sci. 2022 May 11;23(10):5353. doi: 10.3390/ijms23105353 (PMC9140517; doi:10.3390/ijms23105353)
Supplement: Supplementary file 1 [file ijms-23-05353-s001.zip › Table S4 .pdf]

Table S4. BAC sequencing results

BAC clone*	# Contigs	Largest contig (bp)	Total length (bp)	GC%	N50	L50
2F9	19	6,078	24,313	39.36	2,377	3
3A12	14	61,030	195,531	40.14	43,846	2
3I18	110	81,365	259,398	42.19	55,036	2
4B13	144	47,559	248,656	43.25	2,803	9
4N9	34	74,972	181,836	42.45	37,413	2
9C12	28	73,042	212,503	41.49	41,686	2
10F5	18	48,926	125,169	41.05	43,523	2
13L18	121	81,495	289,228	42.87	73,197	2
13O12	24	76,814	221,096	41.38	38,912	2
15B1	47	104,065	219,074	42.13	45,534	2
21I14	4	51,469	80,020	40.51	51,469	1
38B21	31	54,145	189,219	43.07	43,804	2
38H3	125	21,157	117,580	45.17	905	31
39D10	73	124,351	238,145	43.62	124,351	1
39G22	27	45,682	222,432	42.16	32,435	3
42D4	99	14,167	173,087	43.58	2,658	16
42F9	83	38,805	247,618	43.60	27,803	4
44K21	117	35,170	274,794	42.79	3,270	20
45M19	6	99,211	103,934	41.13	99,211	1
47B18	14	6,466	21,855	47.26	6,025	2
47G8	14	37,061	47,250	41.71	37,061	1
51E10	4	16,002	25,326	39.50	16,002	1
53K8	13	12,634	196,986	40.97	126,340	1
54E18	20	55,816	135,720	43.60	26,956	2
54G7	22	50,258	232,384	42.25	29,982	4
54H18	189	96,886	369,972	42.59	14,550	5
57C10	24	70,833	203,648	40.27	51,504	2
57G16	44	23,599	57,838	45.12	1,347	5
57N7	32	75,599	20,289	42.83	13,819	3
62G15	16	35,357	67,435	45.42	35,357	1
65E23	67	24,084	73,262	44.92	1,057	11
65J17	33	44,965	159,366	40.49	42,044	2
67K3	15	64,058	85,663	41.40	64,058	1
67N4	20	57,464	246,438	41.09	31,652	3
67P21	21	71,496	215,946	41.16	47,803	2
67P7	29	76,210	236,533	42.17	59,922	2
68P5	79	3,183	61,464	44.47	737	28

72B11	51	30,428	205,219	41.53	7,286	6
72O12	17	53,994	219,313	40.86	27,508	3
73A11	27	55,264	285,024	41.35	28,235	4
74M4	25	47,812	113,449	43.00	18,012	2
76A22	21	17,593	39,500	43.51	7,918	2
76F9	16	80,658	164,699	42.20	55,865	2

*The BAC 31A2 is not included because it was previously described [22].
