# Supplementary material for: Integration of Maps Enables a Cytogenomics Analysis of the Complete Karyotype in Solea senegalensis
Source: Int J Mol Sci. 2022 May 11;23(10):5353. doi: 10.3390/ijms23105353 (PMC9140517; doi:10.3390/ijms23105353)
Supplement: Supplementary file 1 [file ijms-23-05353-s001.zip › Table S5.pdf]

Table S5. Annotation data of all BAC clones used.

BAC clone	Chromosome location	Annotated genes
**2F9**	**13**	No genes annotated
**3A12**	**21**	*Guanine nucleotide-binding protein subunit alpha-11 (**gna11**), Ubiquitin-conjugating enzyme E2 R2 (**ube2r2**), C2 calcium-dependent domain-containing protein 4C (**c2cd4c**), AP-3 complex subunit delta-1 (**ap3d1**), Hepatoma-derived growth factor-related protein 2 (**hdgfl2**), TLE family member 5 (**tle5**)*
**3I18**	**5**	*Interleukin-11 receptor subunit alpha (**il11ra1**), Cystatin-S (**cst4**), High choriolytic enzyme 1 (**hcea**), Ras association domain-containing protein 6 (**rassf6**), Muscle skeletal receptor tyrosine-protein kinase (**musk**), Ciliary neurotrophic factor receptor subunit alpha (**cntfr**)*
**4B13**	**3**	*Nardilysin (**nrdc**), Protein fam163a (**fam163a**), SLIT and NTRK-like protein 3 (**slitrk3**), Outer dense fiber protein 3-like protein 2 (**odf3l2**), Interleukin-27 subunit beta (**ebi3**), Rab GTPase-activating protein 1 (**rabgap1**)*
**4N9**	**9**	*Immunoglobulin-binding protein 1 (**igbp1**), Uncharacterized protein ccdc142 (**ccdc142**), Histone RNA hairpin-binding protein (**slbp**), ETS-related transcription factor Elf-2 (**elf2**), Transposon TX1 uncharacterized 149 kDa protein ORF 2, Protein ABHD18 (**abhd18**)*
**9C12**	**3**	*Homeobox protein Nkx-2.1 (**nkx2-1**), Homeobox protein Nkx-2.8 (**nkx2-8**), Paired box protein Pax-9 (**pax9**), Mitochondrial 2-oxodicarboxylate carrier (**slc25a21**), Ribosomal protein S6 kinase alpha-5 (**rps6ka5**), Trifunctional enzyme subunit alpha, mitochondrial (**hadha**), Kinesin KIF3B (**kif3b**), MAP3K12-binding inhibitory protein 1 (**mbip**), Doublesex-and mab-3-related transcription factor 2 (**dmrt2**)*
**10F5**	**1**	*Complement component C8 beta chain (**c8b**), Complement component C8 alpha chain (**c8a**), FYN-binding protein 2 (**fyb2**), Phospholipid phosphatase 3 (**plpp3**), Leucine rich adaptor protein 1 (**lurap1**), Post-GPI attachment to proteins factor 6 (**pgap6**), Regulator of G-protein signaling 11 (**rgs11**), Transferrin receptor protein (**trf10**)*
**13L18**	**10**	*Cyclin-dependent kinases regulatory subunit 1 (**cks1b**), Pre-B-cell leukemia transcription factor-interacting protein 1 (**pbxip1**), Tuftelin 1b (**tuft1b**), Cingulin (**cgn**), Pygopus homolog 2 (**pygo2**), SH2 domain-containing adapter protein E (**she**), S100 calcium binding protein S (**s100a11**)*
**13O12**	**7**	*BTB/POZ domain-containing protein kctd15 (**kctd15**), Carbohydrate sulfotransferase 8 (**chst8**), Homeodomain-only protein (**hopx**)*
**15B1**	**18**	*Ankyrin-3 (**ank3**), Ubiquitin-associated domain-containing protein 2 (**ubac2**), Coiled-coil domain-containing protein 6 (**ccdc6**)*
**21I14**	**2**	*Sentrin-specific protease 3 (**senp3**), Peroxisomal acyl-coenzyme A oxidase 3 (**acox3**), RNA-directed DNA polymerase from mobile element jockey, Eukaryotic initiation factor 4A-I (**eif4a1**)*
**38B21**	**12**	*Protein transport protein Sec24C (**sec24c**), NLR family CARD domain-containing protein 3 (**nlrc3**), Calcium/calmodulin-dependent protein kinase type II subunit gamma (**camk2g**), Inactive ubiquitin carboxyl-terminal hydrolase 54 (**ups54**), Myozenin-1b (**myoz1b**), Synaptopodin 2 (**synpo2**), Neurotrypsin (**prss12**), E3 ubiquitin-protein ligase TRIM21 (**trim21**)*
**38H3**	**19**	*Dual serine/threonine and tyrosine protein kinase (**dstyk**)*
**39D10**	**7**	*A disintegrin and metalloproteinase with thrombospondin motifs 17 (**adamts17**), Ceramide synthase 2 (**cers2**), Beta-1,3-galactosyltransferase 1 (**b3galt1**), Cadherin-related family member 5 (**cdhr5**), D(4) dopamine receptor (**drad4**), NAD-dependent protein deacetylase sirtuin-3, mitochondrial (**sirt3**), 26S proteasome non-ATPase regulatory subunit 13 (**psmd13**), Junctophilin-3 (**jph3**), Elongation factor-like GTPase 1 (**elf1**), Cathepsin D (**ctsd**), Uncharacterized protein (**kiaa0895**), Casein kinase II subunit alpha (**csnk2a2**), Zinc finger protein 319 (**znf319**), MORN repeat-containing protein 3 (**morn3**)*
**39G22**	**4**	*Receptor-type tyrosine-protein phosphatase-like N (**ptprn**), Non-homologous end-joining factor 1 (**nhej1**),*

		*Indian hedgehog protein (**ihh**), Myc box-dependent-interacting protein 1 (**bin**), Tubulin alpha-1B chain (**tuba1b**), X-linked retinitis pigmentosa GTPase regulator (**rpgr**), Sodium/potassium-transporting ATPase subunit alpha-1 (**atp1a1**), Aspartyl aminopeptidase (**dnpep**), Desmin (**des**), Transmembrane protein 198 (**tmem198**), Sterol 26-hydroxylase, mitochondrial (**cyp27a**), Mitogen-activated protein kinase kinase kinase 2 (**map3k2**)*
**42D4**	**2**	*Synaptotagmin-1 (**syt1**), NADH-cytochrome b5 reductase 3 (**cyb5r3**), Liprin-beta-1 (**ppfibp1**), Translation initiation factor eIF-2B (**eif2b2**), Glutathione transferase (**fosa**), Jun dimerization protein 2 (**jdp2**), YLP motif-containing protein 1 (**ylpm1**), Sphingosine-1-phosphate phosphatase 1 (**sgpp1**), Potassium voltage-gated channel subfamily H member 5 (**kcnh5**), Argininosuccinate synthase (**ass2**)*
**42F9**	**17**	*T-lymphoma invasion and metastasis-inducing protein 1; rho guanine nucleotide exchange factor TIAM1 (**tiam1**), Superoxide dismutase [Cu-Zn] (**sod1**), Hormonally up-regulated neu tumor-associated kinase homolog A (**hunka**), Solute carrier family 46 member 3 (**slc46a3**), Synaptojanin-1 (**synj1**), Protein Mis18-alpha (**mis18a**), HAUS augmin-like complex subunit 1 (**haus1**), Protein eva-1 homolog C (**eva1c**), Cilia- and flagella-associated protein 298 (**cfap298**), Protocadherin-16 (**dchs1**), SR-related and CTD-associated factor 4 (**scaf4**), Rab GTPase-activating protein (**rabgap1l2**)*
**44K21**	**7/15**	*GTP-binding protein RAD (**rrad**), Cadherin-16 (**cdh16**), ATP synthase F(0) complex subunit C3, mitochondrial (**atp5mc3**), tRNA (cytosine(34)-C(5))-methyltransferase, mitochondrial (**nsun3**), Transmembrane protein 100 (**tmem100**), Ectonucleotide pyrophosphatase/ phosphodiesterase family member 7 (**enpp7**), Chromobox protein homolog 2 (**cbx2**), Phosphatidylcholine transfer protein (**pctp**), Small integral membrane protein 36 (**smim36**), Monocyte to macrophage differentiation factor (**mmd**), Hepatic leukemia factor/HLF transcription factor, PAR bZIP family member a (**hlf**), Noggin-1 (**nog1**)*
**45M19**	**20**	*Inositol polyphosphate 5-phosphatase Ka (**inpp5ka**), Phosphatidylinositol 4-phosphate 5-kinase type-1 alpha (**pip5k1a**), Phosphatidylinositol transfer protein alpha (**pitpna**), Large neutral amino acids transporter small subunit 4 (**slc43a2**), Unconventional myosin-Ic (**myo1c**)*
**47B18**	**6**	*No genes annotated*
**47G8**	**7**	*Substance-K receptor (**tacr2**), Tetraspanin-15 (**tspan15**)*
**51E10**	**9**	*Homeodomain-only protein (**hopx**), Testicular haploid expressed gene protein (**theg**)*
**53K8**	**20**	*Vacuolar protein sorting-associated protein 53 (**vps53**), refilin-B (**rflnb**), tRNA-dihydrouridine (20a20b) synthase [NAD(P)+] (**dus4**), Solute carrier organic anion transporter family member 1C1 (**slco1c1**)*
**54E18**	**16**	*Zinc finger protein (**zfpm2**)*
**54G7**	**19**	*Protein FAM72A (**fam72a**)*
**54H18**	**3**	*MAP/microtubule affinity-regulating kinase 3a (**mark3a**), Eukaryotic translation initiation factor 5 (**eif5**), Vacuolar protein sorting-associated protein 29 (**vps29**), Tumor necrosis factor alpha-induced protein 2 (**tnfaip2**), Exocyst complex component 3-like protein 4 (**exoc3l4**), Dr1-associated corepressor (**drap1**), Activator of 90 kDa heat shock protein ATPase homolog 1 (**ahsa1**), Cell division cycle-associated protein 4 (**cdca4**), MAP/microtubule affinity-regulating kinase 3 (**mark3**), Creatine kinase B (**ckb**), tRNA (adenine(58)-N(1))-methyltransferase catalytic subunit TRMT61A (**trmt61a**)*
**57C10**	**8**	*Leucine-rich repeat and immunoglobulin-like domain-containing nogo receptor-interacting protein 1 (**lingo1**)*
**57G16**	**9**	*Integral membrane protein 2A (**itm2a**), Probable G-protein coupled receptor 174 (**gpr174**), Disks large-associated protein 2 (**dlgap2**), Rreceptor-type tyrosine-protein phosphatase kappa (**ptprk**)*
**57N7**	**10/12/16**	*Microtubule-actin cross-linking factor 1 (**macf1**), Cornifelin (**cnfn**)*
**62G15**	**19**	*Forkhead box protein P4 (**foxp4**)*

**65E23**	**2**	*39S ribosomal protein L52, mitochondrial (**mrpl52**), LIM domain-containing protein ajuba (**ajuba**), Apoptotic chromatin condensation inducer 1b (**acin1b**), Brorin (**vwc2**), DNA-binding protein Ikaros (**ikzf1**), Cytochrome P450 1B1 (**cyp1b1**), DNA-directed RNA polymerase I subunit RPA2 (**polr1b**), DENN domain-containing protein 10 (**dennd10**), Eukaryotic translation initiation factor 3 subunit A (**eif3a**), DNA-directed RNA polymerase I subunit RPA2 (**polr1b**), N-acylneuraminate-9-phosphatase (**nanp**), Transmembrane 9 superfamily member 3 (**tm9sf3**), Fidgetin-like protein 1 (**fignl1**), Tubulin--tyrosine ligase (**ttll12**)*
**65J17**	**13**	*Kelch-like protein 10 (**klhl10**), CD40 ligand (**cd40**), Formin-like protein 13 (**fh13**) en Oryzias, en humanos no viene la proteina 13, Prefoldin subunit 4 (**pfdn4**), Breast carcinoma-amplified sequence 1 (**bcas1**), N-acetyltransferase 8 (**nat8**), Cerebellin-4 (**cbln4**), iroquois homeobox 7 (**irx7**)*
**67K3**	**4**	*Protein Wnt-10a (**wnt10a**), Protein Wnt-6 (**wnt6**), Mitocondrial FAST kinase domain-containing protein 3 (**fastkd3**), Cilia- and flagella-associated protein 65 (**cfap65**)*
**67N4**	**1**	*Kelch-like protein 24 (**klhl24**), Probable G-protein coupled receptor 148 (**gpr148**), Mitocondrial transcription termination factor 4 (**mterf4**), PAS domain-containing serine/threonine-protein kinase (**pask**), Voltage-gated potassium channel subunit beta-1 (**kcnab1**), Muscleblind-like protein 1 (**mbnl1**), Cullin-3 (**cul3**), Dehydrogenase/reductase SDR family member 12 (**dhrs12**), AP-1 complex subunit sigma-3 (**ap1s3**), Secretogranin-2 (**sgc2**), Fatty acid CoA ligase (**acsl3**), Paired box protein Pax-3 (**pax3**), Zonadhesin (**zan**), Complement C1q-like protein 2 (**c1ql2**)*
**67P21**	**1**	*Adcyap1b protein (**adcyap1b**), Tyrosine-protein kinase Yes (**yes1**), Clusterin-like protein 1 (**clul1**), Collectin-12 (**colec12**), Signal transducing adapter molecule 1 (**stam**), Transmembrane protein 236 (**tmem236**), Voltage-dependent L-type calcium channel subunit beta-2 (**cacnb2b**), Heterogeneous nuclear ribonucleoprotein A0 (**hnrnpa0**), Macrophage mannose receptor 1 (**mrc1**), MARVEL domain-containing protein 1 (**mald1**)*
**67P7**	**6**	*Integrin alpha-2 (**itga2**), Polyadenylate-binding protein-interacting protein 1 (**paip1**), A disintegrin and metalloproteinase with thrombospondin motifs 12 (**adamts12**), Glycerol-3-phosphate acyltransferase 3 (**agpat9**), Hexokinase-4 (**gck**), Beta-2 adrenergic receptor (adrb2), hypermethylated in cancer 2 protein (**hic2**), G-protein coupled receptor 15 (**grp15**), Mu-type opioid receptor (**oprm1**), Ovarian cancer G-protein coupled receptor 1 (**gpr68**), Interleukin-12 subunit beta (**il12b**), U3 small nucleolar RNA-associated protein 15 (**utp15**), Synaptobrevin homolog YKT6 (**ykt6**), Oncostatin-M-specific receptor subunit beta (**osmr**), Rho guanine nucleotide exchange factor 28 (**arhgef28**)*
**68P5**	**2**	*Phosphate carrier protein, mitochondrial (**slc25a3**), Ubiquitin carboxyl-terminal hydrolase 46 (**usp46**), Solute carrier family 25 member 3a (**slc25a3a**), SLAIN motif-containing protein 2 (**slain2**), Protein furry homolog (**fryl**), Ras-like protein family member 11B (**rasl11b**), Sodium/bile acid cotransporter 4 (**slc10a4**), Beta-sarcoglycan (**sgcb**), Itochondria-eating protein (**spata18**)*
**72B11**	**5/9/11**	*Disks large-associated protein 2 (**dlgap2**), Receptor-type tyrosine-protein phosphatase kappa (**ptprk**), Microtubule-associated protein 1B (**map1b**), Probable G-protein coupled receptor 174 (**gpr174**), Integral membrane protein 2A (**itm2a**), Carboxyl-terminal PDZ ligand of neuronal nitric oxide synthase protein (**nos1ap**), Pentatricopeptide repeat-containing protein 2 (**ptcd2**), Microtubule-associated protein 1B (**map1b**), Calcium channel flower homolog (**cacfd1**)*
**72O12**	**21**	*Protein furry homolog (**fryl**), SLAIN motif-containing protein 2 (**slain2**), Sec1 family domain-containing protein 2 (**scfd2**), Ubiquitin carboxyl-terminal hydrolase 46 (**usp46**), Ras-like protein family member 11B (**rasl11b**), Spermatogenesis-associated protein 18 (**spata18**), Beta-sarcoglycan (**sgcb**), Epidermal growth factor receptor substrate 15 (**eps15**), Anaphase-promoting complex subunit 4 (**anapc4**),*

		*SLAIN motif-containing protein 2 (**slain2**), Zygote arrest protein 1 (**zar1**), Sodium/bile acid cotransporter 4 (**slc10a4**), DCN1-like protein 4 (**dcun1d4**), OCIA domain-containing protein 2 (**ociad2**)*
**73A11**	**3/14**	*MAPK/MAK/MRK overlapping kinase (**mok**), Nuclear export mediator factor (**nemf**), DNA polymerase epsilon subunit 2 (**pole2**), Kelch domain-containing protein 1 (**klhdc1**), Charged multivesicular body protein 4a (**c14orf123**), B2 bradykinin receptor (**bdkrb2**), B1 bradykinin receptor (**bdkrb1**), Psychosine receptor (**gpr65**), K2P10.1 potassium channel subunit (**kcnk10b**), Spermatogenesis-associated protein 7 (**spata7**), Zeta-sarcoglycan (**sgcz**)*
**74M4**	**5**	*Carboxyl-terminal PDZ ligand of neuronal nitric oxide synthase protein (**nos1ap**), Microtubule-associated protein 1B (**map1b**), A disintegrin and metalloproteinase with thrombospondin motifs 12 (**adamts12**)*
**76A22**	**16**	*Clusterin (**clul1**), Collectin-12 (**colec12**), Tyrosine-protein kinase Yes (**yes1**)*
**76F9**	**7**	*BTB/POZ domain-containing protein kctd15 (**kctd15**), Transcription initiation factor TFIID subunit 4 (**taf4**), LSM14A mRNA processing body assembly factor b (**lsm14a**), SS18-like protein 2 (**ss18l2**), Granule associated Rac and RHOG effector protein 1 (**garre1**), Glucose-6-phosphate isomerase (**gpi**)*
