# Supplementary material for: Integration of Maps Enables a Cytogenomics Analysis of the Complete Karyotype in Solea senegalensis
Source: Int J Mol Sci. 2022 May 11;23(10):5353. doi: 10.3390/ijms23105353 (PMC9140517; doi:10.3390/ijms23105353)
Supplement: Supplementary file 1 [file ijms-23-05353-s001.zip › Table S7_modified.pdf]

Table S7. *Scophthalmus maximus* orthologue localization of each BAC clones analyzed in *Solea senegalensis* and number of genes found within.

Chromosome of *S. maximus*	Chromosome of *S. senegalensis*	BAC	Nº genes within the BAC	Nº genes found in *S. maximus*
1	4	12N15	12	5
	2	21I14	3	3
	4	12N15	12	7
	4	39G22	12	12
	16	71N11	11	11
	12	57N7	2	1
	16	52E17	20	20
	16	25P16	5	4
	16	54E18	1	1
2	2	46C5	13	13
	2	60P19	9	7
	2	4D15	5	2
	2	4D15	5	3
	2	36I3	2	2
	2	65E23	13	3
	2	21O23	3	2
	2	52G10	14	4
	2	42D4	10	3
	2	52G10	14	8
3	20	45M19	5	4
	20	1C2	1	1
	20	53K8	4	4
4	17	42F9	12	11
	17	63A7	7	7
	17	31N1	2	2
	2	36K1	1	1
	17	19K18[11]	5	4
	17	65I16[11]	2	2
	2	38N10	9	9
5	7	39D10	14	11
	7	7H22	5	5
	7	19H9	3	3
	15	44K21	12	4
	7	47G8	2	2
	7	13O12[7]	3	2
	7	76F9[7]	6	6
	7	39D10	14	3
6	19	31C1	19	17
	19	50K3	11	11
	19	38H3	1	1
	19	31C1	19	1
	19	42P4[10]	8	8
	19	62G15	1	1
	11	45L11[8]	11	1

	19	12K16	1	1
	16	9N8	2	1
	11	45L11[8]	11	2
7	1	5K5[2]	14[3]	9
	16	76A22	3	2
	1	67P21	10	10
	1	48P7	8	8
	1	67N4	14	14
	1	10F5	8	8
	1	13G1	1	1
	1	12D22	7[3]	2
	10	9E8	3	3
8	14	29D4	4	4
	14	4N21	8	8
	14	6P22	21	17
9	6	64A8	5	1
	6	64A8	5	1
	6	20D18[5]	8	7
	6	31A1	6	6
	6	67P7	14	14
	6	64A8	5	3
	6	48K7[6]	6	6
10	8	8O7	8	8
	8	57C10	1	1
	8	31A2	3	3
	2	19J21	2	1
	8	46P22	2	2
11	13	19L16	4	4
	13	65J17	8	8
	13	4M14	5	5
12	21	72O12	13	1
	21	55B12	5	5
	21	3A12	6	6
	21	30H22	8	8
	21	72O12[12]	13	12
	21	63A3	10	9
13	9	51E10	2	2
	9	32B8	10	10
	9	39F2	4	4
	5	72B11	9	2
	9	4N9	5	4
14	4	8A23	10	10
	4	67K3	4	4
	4	3C15	3	3
	4	46B2	7	7
	4	12D24	12	12
	4	30P17	3	3
	4	36J2	1	1
	4	36H3	3	1

15	18	15B1	3	3
	18	3F15	3	3
	18	36M2	1	1
	2	65E23	13	9
	18	2K18	5	1
	18	2K18	5	3
16	5	3I18	6	6
	11	45L11	11	1
	16	53D20	7	1
	16	53D20	7	6
	5	72B11[4]	9	4
	16	9N8	2	1
17	3	54H18	11	9
	3	9C12	9	8
	3	4B13	6	6
	3	73A11	11	10
	3	9J4	12	12
18	1	52C17	12	12
	12	35D17	1	1
	12	38B21	8	8
	12	13E1	3	3
19	15	36E3	2	2
	15	22C2	4	4
	15	60P24	1	1
	15	4F12	4	4
	15	44K21	12	8
20	11	3N10	3	3
	11	38F24	3	3
	11	31F1	1	1
	2	42D4	10	7
	11	45L11[8]	11	5
	5	72B11	9	2
21	1	10K23[1]	6	4
	1	36D3	1	1
22	10	15I19	7	7
	10	68G4	7	6
	12	57N7	2	1
	10	68G4	7	1
	10	13L18[9]	7	7
	10	56H24[9]	9	9

[1] BAC 73B7 contains 3 genes present in BAC 10K23.

[2] BAC 10L10 contains 4 genes present in BAC 5K5 and 2 genes shared with BAC 10K23.

[3] Five of these genes belong to the histone cluster.

[4] BAC 74M4 contains 2 genes present in BAC 72B11

[5] BAC 11020 contains 3 genes present in BAC 20D18.

[6] BAC 16E16 contains 2 genes present in BAC 48K7.

[7] BAC 13O12 overlaps in one gene with BAC 76F9.

[8] BAC 4E10 contains 2 genes present in BAC 45L11.

[9] BAC 13L18 overlaps in one gene with BAC 56H24.

[10] BAC 54G7 contains 1 gene present in BAC 42P4.
[11] BAC 19K18 overlaps in one gene with BAC 65I16.
[12] BAC 68P5 contains 7 genes present in BAC 72O12.
